# First Clinical Experience of Intra-Operative High Intensity Focused Ultrasound in Patients with Colorectal Liver Metastases: A Phase I-IIa Study

**DOI:** 10.1371/journal.pone.0118212

**Published:** 2015-02-26

**Authors:** Aurélien Dupré, David Melodelima, David Pérol, Yao Chen, Jérémy Vincenot, Jean-Yves Chapelon, Michel Rivoire

**Affiliations:** 1 Department of Surgical Oncology, Centre Léon Bérard, Lyon, France; 2 LabTau, U1032, Inserm, Université de Lyon, Lyon, France; 3 Biostatistics and Treatment Evaluation Unit, Centre Léon Bérard, Lyon, France; The Chinese University of Hong Kong, HONG KONG

## Abstract

**Background:**

Surgery is the only curative treatment in patients with colorectal liver metastases (CLM), but only 10–20% of patients are eligible. High Intensity Focused Ultrasound (HIFU) technology is of proven value in several indications, notably prostate cancer. Its intra-operative use in patients with CLM has not previously been studied. Preclinical work suggested the safety and feasibility of a new HIFU device capable of ablating volumes of up to 2cm x 2cm in a few seconds.

**Methods:**

We conducted a prospective, single-centre phase I-IIa trial. HIFU was delivered immediately before scheduled hepatectomy. To demonstrate the safety and efficacy of rapidly ablating liver parenchyma, ablations were performed on healthy tissue within the areas scheduled for resection.

**Results:**

In total, 30 ablations were carried out in 15 patients. These ablations were all generated within 40 seconds and on average measured 27.5mm x 21.0mm. The phase I study (n = 6) showed that use of the HIFU device was feasible and safe and did not damage neighbouring tissue. The phase IIa study (n = 9) showed both that the area of ablation could be precisely targeted on a previously implanted metallic mark (used to represent a major anatomical structure) and that ablations could be undertaken deliberately to avoid such a mark. Ablations were achieved with a precision of 1–2 mm.

**Conclusion:**

HIFU was feasible, safe and effective in ablating areas of liver scheduled for resection. The next stage is a phase IIb study which will attempt ablation of small metastases with a 5 mm margin, again prior to planned resection.

**Trial Registration:**

ClinicalTrials.govNCT01489787

## Introduction

Managing colorectal liver metastases (CLM) is a major clinical challenge, and surgery remains the only potentially curative treatment. Five-year survival rates of up to 51% have recently been reported [1]. The development of complex surgical techniques such as two-stage hepatectomy [2], in association with the use of neoadjuvant chemotherapy and targeted agents, has increased the resectability rate [3]. However, despite this progress, only 10–20% of patients are eligible for surgery, which is often precluded by the number, size and/or location of metastases, or because the necessary resection will leave insufficient volume of functional liver [4].

Techniques involving focal destruction, such as cryotherapy or radiofrequency ablation (RFA), which were initially developed for percutaneous treatment, have been used in association with surgery as a tool to expand the number of patients who may be candidates for liver-directed therapy. However, conventional techniques of focal destruction have several limitations. There is a risk of inadequate treatment due to the heat sink effect of blood flow, they do not allow reliable real-time monitoring, and they require intra-parenchymal introduction of a probe. Moreover, only small hepatic volumes can be targeted. These limitations could explain the high rates of local recurrence seen after percutaneous RFA. A pooled analysis found a local recurrence rate of 24% after percutaneous RFA for solitary CLM [5], and two prospective studies of RFA for unresectable CLM reported local recurrence in 4% and 16.1% of patients [6,7].

High intensity focused ultrasound (HIFU) is a recent technology that uses ultrasound energy to destroy targeted tissue. The tissue temperature at the focal point rises to 70–90°C in a few seconds, resulting in irreversible destruction by coagulative necrosis. Because this is achieved so quickly, there is little cooling due to perfusion of the target area or adjacent tissue [8]. HIFU has been proven effective in a wide range of clinical applications, especially prostate cancer [9,10].

The ablation achieved by conventional HIFU is small and ellipsoidal. The dimensions vary according to transducer characteristics but are typically 1–3 mm (transverse) and 8–15 mm (along beam axis) [11]. In clinical practice, hundreds of superimposed ablations are required; and the procedure may take up to two hours [12]. Even so, HIFU has several potential advantages in the treatment of liver tumours: there is no need to puncture the parenchyma, the extent of the thermal lesions achieved is not reduced by hepatic perfusion, and it is possible to monitor the effects of therapy in real time [13]. However, extra-corporeal treatment of the liver is difficult because presence of the ribcage may stop propagation of ultrasound waves and respiratory motion may cause targeting problems.

HIFU treatment of CLM needs to be improved, and reducing the duration of surgical intervention by increasing the size of ablated fields is particularly important. A new and powerful HIFU device enabling destruction of larger liver volumes has been developed based on toroidal transducers [14]. Preliminary *in vitro* and preclinical work demonstrated the potential, feasibility and safety of such HIFU ablations [8,15,16]. During laparotomy in a porcine model we demonstrated that this HIFU device achieves reproducible ablations with an average volume of 7 cm^3^ (with 20 mm diameter and 25 mm long axis) in 40 seconds [13]. Such preclinical work has to be translated into clinical practice through controlled trials, and the aim of this study was to assess the feasibility and safety of HIFU ablation in patients undergoing hepatectomy for CLM, as well to collect efficacy and accuracy data. This study is registered with ClinicalTrials.gov (NCT01489787).

## Patients, Materials and Methods

The protocol for this trial and supporting TREND checklist are available as supporting information; see S1 Checklist and S1 Protocol.

**Fig 1:**
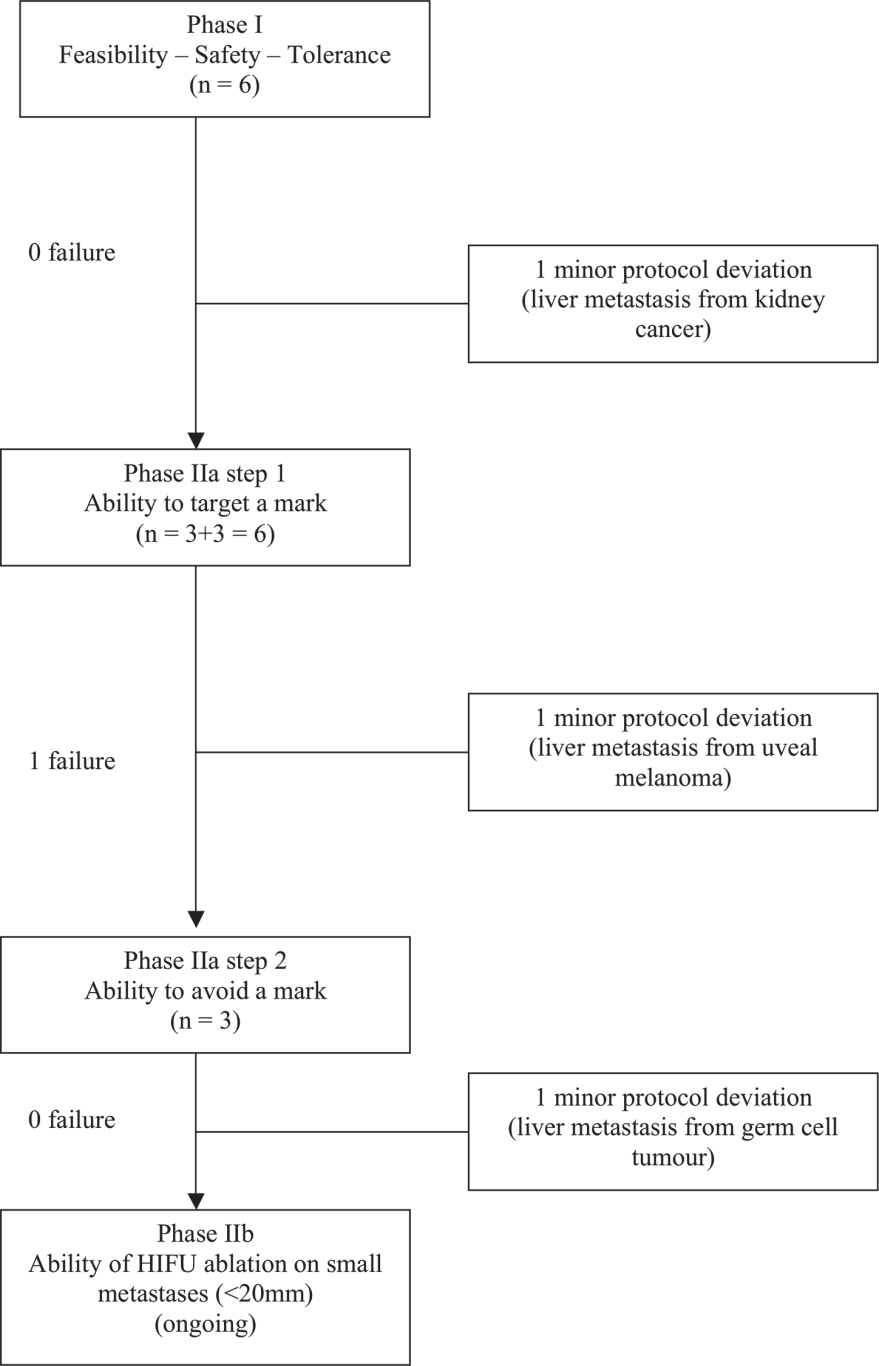
Flowchart.

We conducted a prospective, single-centre phase I/II study evaluating the feasibility, safety and accuracy of HIFU during surgery in patients with CLM. The protocol was reviewed and validated by a national ethics committee (CPP Sud-Est IV) according to French and European directives. Ethics approval was obtained on 24 September 2009. Name of the IRB: Comité de Protection des Personnes (CPP) SUD-EST IV; N° IRB: 09/070; N° of the approval number: A 09–205. Patients gave written informed consent. The study was designed to have three phases (I, IIa and IIb). Only phases I and IIa are described in this paper (Fig. 1). Since this study was the first use in man of intra-operative hepatic HIFU, ablations were made only in areas of liver scheduled for resection. This allowed real-time evaluation of HIFU ablation while protecting participating patients from any adverse effects related to this new technique. During phases I and IIa, designed to demonstrate the efficacy of the HIFU device in ablating large areas, portions of healthy liver adjacent to the metastases were ablated. Only areas included within the planned resection were treated with HIFU. At this stage, we did not attempt to ablate metastases themselves.

As this is a proof-of-concept study, it follows the IDEAL recommendations [17] and corresponds to stage I (innovation) and IIa (development). It was prospectively registered and we conformed to standards for protocol amendment when making changes between registry entry and final reporting [18]. We used the Transparent Reporting of Evaluations with Non-randomised Designs (TREND) guidelines [19] when preparing this paper for publication. The study was registered after the enrolment of participants due to an administrative mistake. The authors confirm that all ongoing and related trials for this medical device are registered.

### Patients

Patients with resectable CLM between January 2010 and November 2011 were evaluated for this study. To be eligible, patients were at least 18 years old, in good clinical condition (Eastern Co-operative Oncology Group ECOG/World Health Organization WHO performance status 0–1) and not pregnant. Patients gave written informed consent. Patients were not eligible if they had had previous liver, biliary or major abdominal surgery. Those who could not be followed for the duration of the study were also excluded.

### Study procedure

**Fig 2:**
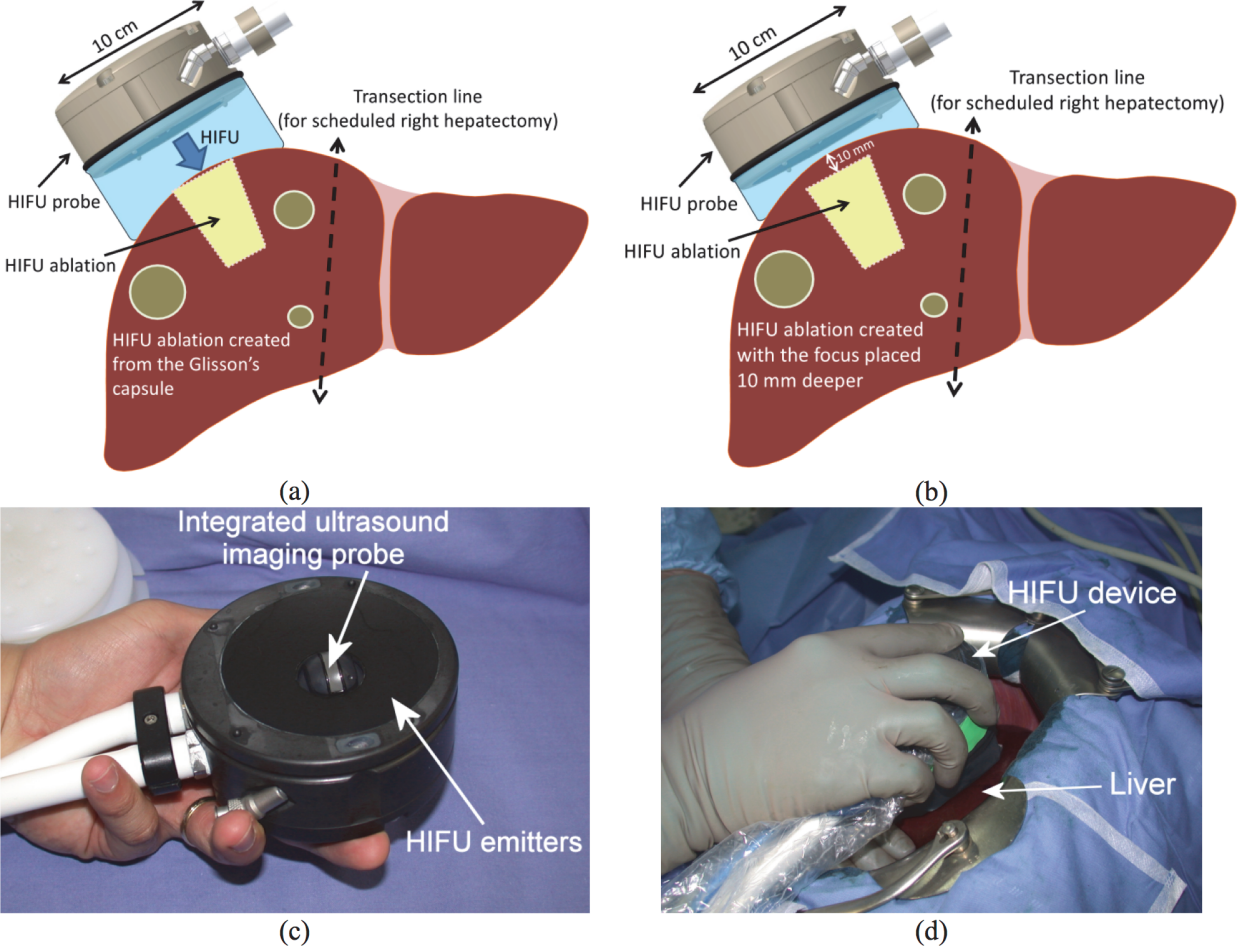
HIFU Probe and schematic views of the ablated area. (a) Schematic diagram of a HIFU treatment with the HIFU focus placed in the liver such as HIFU ablations extent from the Glisson’s capsule. (b) Using less water in the cooling envelope the focus can be placed deeper in tissues (10 mm) such as HIFU ablations can be created deeper in the liver. (c) Photograph of the HIFU device. (d) Intraoperative view of the HIFU device.

A trained operating room nurse prepared the HIFU probe. Ultrasound imaging was monitored and recorded for subsequent analysis. The procedure was identical to that patients would have undergone if not included in the study except that—to demonstrate the efficacy of our device in ablating large areas—portions of healthy liver adjacent to metastases were ablated. However, this was done only when these areas were included within the planned resection. For example, ablation could include a healthy segment VI in a patient requiring a right hemi-hepatectomy for oncologic and technical reasons (Fig. 2).

After initial preparation and liberation of the right liver from its ligaments, protective gauzes were put under the targeted area to inhibit ultrasound propagation and unintended HIFU damage to adjacent organs. The HIFU probe was brought into contact with the liver surface through an ultrasound coupling liquid contained in a sterile envelope covering the sterilized HIFU probe (Fig. 2). The user interface displayed the position of the HIFU treated region superimposed on the sonogram obtained with the help of the Integrated UltraSound imaging Probe (IUSP). We could therefore locate the ablation and visualize in real time the treated zone created during ultrasound exposures. The target of treatment was defined as previously described [13].

### HIFU equipment

The HIFU device was similar to that previously reported in animal studies [13]. The ultrasound (US) fields were generated with eight US emitters (each divided into 32 transducers) operating at a frequency of 3 MHz and distributed according to a toroidal geometry. The radius of curvature of the transducer was 7 cm and the diameter was 7 cm. Compared with conventional HIFU devices, this toroidal geometry produces two focal zones allowing the precise ablation of a large volume of liver tissue (diameter of 2 cm with a major axis of 2.5 cm) in 40 seconds [20]. As shown in Fig. 2, a 7.5 MHz ultrasound IUSP (Vermon, Tours, France) was placed in the centre of the device and connected to a BK HAWK 2102 EXL scanner (B-K Medical, Herlev, Denmark). The ultrasound imaging plane was aligned with the HIFU acoustic axis in order to position the region to be treated and visualize the ablation with the same device. The HIFU probe was brought into contact with the liver using a sterile ultrasound cooling and coupling liquid (Ablasonic, Edap- Technomed, Vaulx en Velin, France) contained in a sterile envelope (CIV-Flex Transducer Cover; CIVCO, Kalona, IA, USA) covering the HIFU probe. The focal ring created by the toroidal transducer was located at 7 cm. A secondary focal zone corresponding to the intersection of the ultrasound beams emitted by the toroidal transducer was located at 8.6 cm. The location of the focal zones could be changed by electronic focusing or by adjusting the quantity of liquid between the device and the tissue allowing to ablate a deep-seated area. The user interface allowed sonogram visualization of the region to be coagulated by HIFU. A target representing this region was superimposed on the ultrasound image and served as a guide for the surgeon.

### Objectives

The primary endpoints of the phase I study were the feasibility, safety and tolerability of HIFU ablation. Feasibility was assessed by the ability to use MFOCUS in aseptic conditions and to carry out two HIFU ablations (one superficial and one with the focal zone placed at least 1cm deeper) in each patient without prolonging the surgical procedure by more than 30 minutes. A depth of 1cm from the capsule was chosen to prove the possibility of ablating deep-seated areas without risking to ablate tissues behind the liver as the right lobe of the liver was mobilized. The safety of the device was assessed by absence of injury to non-target tissues: the abdominal cavity was explored at the end of the liver resection to detect possible adverse effects, especially burns, involving organs adjacent to the liver. Tolerability was assessed by the stability of vital signs during and for five minutes after the HIFU ablations.

Secondary endpoints were ease of use of the probe and reliability of the IUSP. Ease of use was assessed by the ability to adjust the probe position to cover all eight liver segments irrespective of their conformation in the individual patient. The ultimate objective was to be able to generate an HIFU ablation in at least 80% of the whole liver, represented by 15 anatomical structures. The reliability of the IUSP was assessed by the feasibility of visualizing and targeting.

The primary endpoint of the phase IIa study was to establish the accuracy of HIFU in relation to a metallic O-twist-marker (BIP GmbH, Türkenfeld, Germany) with a diameter of 5 mm implanted in the liver parenchyma to mimic a major anatomical structure. Our first objective was to ablate liver parenchyma all around this metallic mark (step 1), and then to destroy liver parenchyma at a fixed distance from this mark while avoiding it (step 2). The secondary endpoint was safety, assessed by the same criteria as during phase I.

Common secondary endpoints were the feasibility of real time monitoring of the HIFU ablation and pathological description. Measures obtained by ultrasonography of the ablations (with the IUSP) and from gross pathological examination of the surgical specimen were assessed and correlated. Ablated and surrounding tissues were also examined histologically.

### Follow-up

The study population consisted of all enrolled patients who were eligible for hepatectomy. All were followed for thirty days according to our institution’s guidelines for patients operated on for CLM (i.e. between March 2010 and December 2011).

### Sample size

The two-step phase I design allowed early termination of enrolment if an unacceptable global failure rate was observed following Lee’s sequential criteria [21]. According to these criteria, the enrolment was considered to be closed if a failure was observed in at least 2/2 and 3/6 patients in the first and the second step, respectively. Failure was defined as the occurrence of at least one of the following: a longer than 30 minute period required for the two HIFU ablations; the inability to perform ablations with the focal zone placed at the right distance from Glisson’s capsule; failure to maintain asepsis while using the device; accidental injury to adjacent organs; and lack of tolerability (defined by >10% change from baseline in vital signs). Cohorts of 3 to 6 evaluable patients were enrolled in both steps of phase IIa which had to include at least six patients. This part of the study aimed to determine the accuracy of HIFU ablation in terms of its success both in targeting a mark (step 1) and in avoiding damage to tissues at a given distance from this mark (step 2). During step 2, success was defined by achieving a distance between the metallic mark and the closest ablation edge of 7.5 mm. The tolerated range around the ideal distance of 7.5 mm was 1–15 mm. Based on binomial probabilities, in 3 and 6 patients, respectively, there was a 90% probability of observing one or more patient with a failure, if that failure occurred in at least 54% and 32% of the population, respectively.

At the end of each phase, results were evaluated by an independent steering committee which decided whether to proceed to the next stage according to the rules outlined.

### Histopathological examination

All excised specimens were evaluated by gross and histological examination. Areas of HIFU ablation and areas of non-affected CLM and normal liver tissue were separately analysed. Ablations were cut along the exposure axis. Dimensions of the ablated area were measured macroscopically with a ruler and also on images using Image J software (Wayne Rasband, National Institutes of Health, Bethesda, MD, USA). Each ablation was then sliced into 5 mm sections and macroscopic homogeneity was determined. Histopathological and immunohistological examinations were done after ablated areas were fixed in 10% phosphate-buffered formalin (pH = 7), embedded in paraffin, and stained with haematoxylin and eosin.

For immunohistological analyses, the 4 μm liver sections were deparaffinised and endogenous peroxidase activity blocked with H2O2. The sections were then incubated with purified primary monoclonal antibody against hepatocyte antigen, glutamine synthetase and cytokeratin 18 as markers of hepatocytes, cytokeratin 7 as a marker of biliary cells, CD68 as a marker of Küppfer cells, CD31 as a marker of endothelial cells, (Ventana Medical Systems, Tucson, AZ, USA) at 37°C for 32 minutes and with secondary biotinylated anti-IgG (BA 2001; Vectorlabs, Burlingame, CA, USA). Streptavidin was used as chromogen substrate. Analyses were undertaken by a single pathologist.

### Data analysis

Demographic and baseline characteristics, safety and accuracy observations and measurements were summarized using descriptive statistics (quantitative data) and contingency tables (qualitative data). The association between variables was tested using Spearman’s rank correlation coefficient. All analyses were undertaken using SAS version 9.1.

## Results

Between March 2010 and November 2011, 15 patients were enrolled, six during phase I and six and three during step 1 and step 2 of phase IIa, respectively (Fig. 1). All patients had a right or extended right hepatectomy, thirteen for CLM, one for liver metastases from kidney cancer, one for metastases from uveal melanoma and one for metastases from tumour germ cells (considered as 3 minor study protocol deviations). Characteristics of the patients and primary tumours are shown in Table 1.

**Table 1:** Patient demographics and tumour characteristics.

	Total (n = 15)
Age (years)	57 (41–74)
Sex ratio M/F	6/9
BMI	24.6 (18.8–31.5)
ASA status	
I	2
II	13
Primary tumour site	
Right colon	3
Left colon	7
Rectum	2
Other*	3
Liver resection planned	
Right hepatectomy	9
Right hepatectomy + segment IV	5
Right hepatectomy + segment I	1

Continuous variables are described as median (min-max).

BMI indicates body mass index; ASA, american society of anesthesiologists.

* Liver metastases from kidney cancer, uveal melanoma and germ cell tumour.

### Phase I

**Fig 3:**
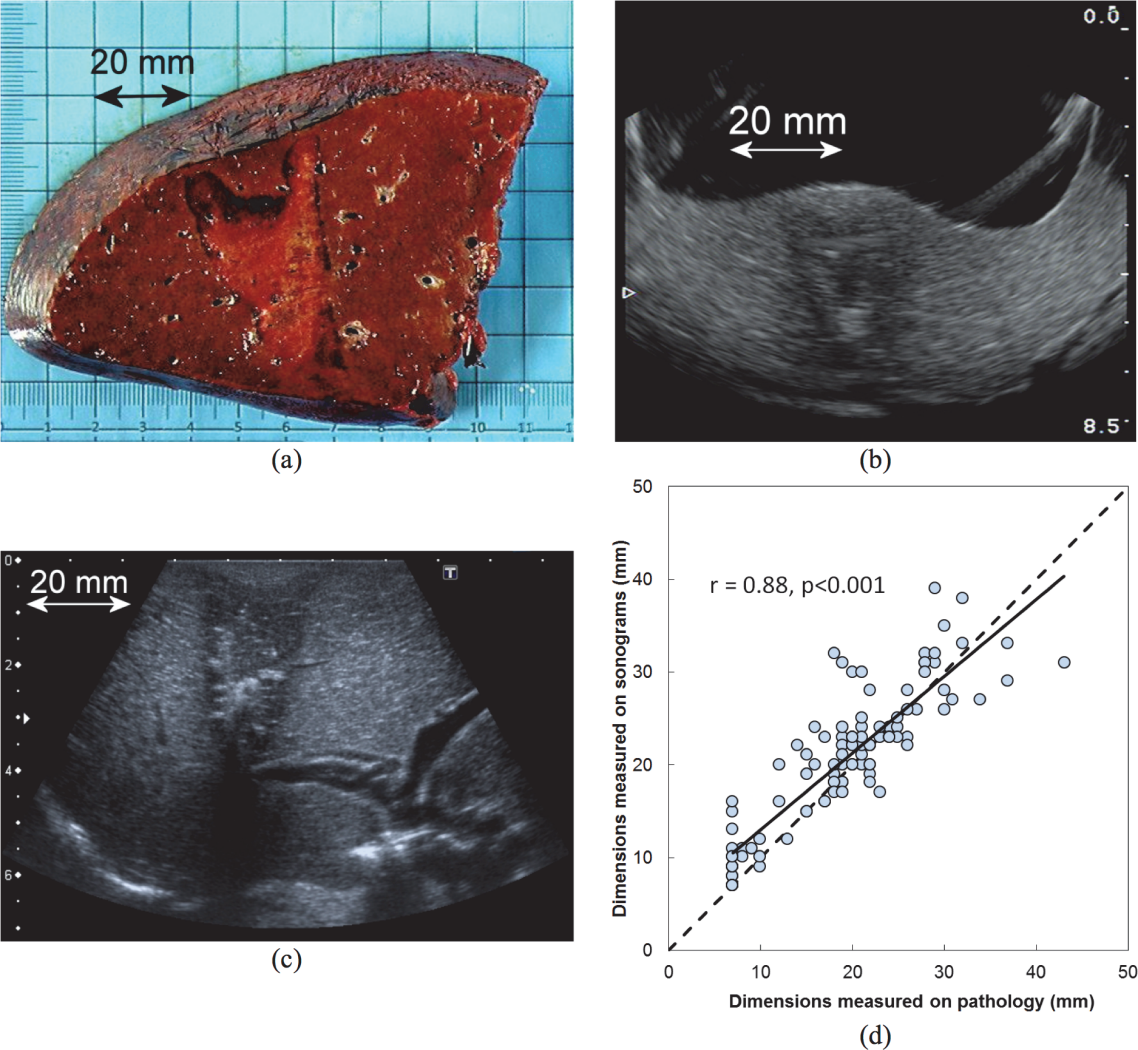
Macroscopic and echographic visualzation of HIFU ablated area. HIFU ablations were clearly visible in ultrasound images. Dimensions measured on sonograms were correlated to the dimensions measured on gross pathology. (a) Typical HIFU ablation observed on gross pathology, (b) on sonograms obtained with the integrated ultrasound imaging probe (IUSP) of the HIFU device and (c) on sonograms obtained with a higher resolution ultrasound imaging probe. (d) Correlation between dimensions measured on sonograms using IUSP and gross pathology.

Each of the six phase I patients had successful superficial and deep HIFU ablations. For the deepest HIFU ablation, the focus was placed at a mean depth of 12 ± 2 (10–20) mm, resulting in a mean distance between Glisson’s capsule and the top of the ablated area of 7 ± 4 (2–13) mm. An example of deep-seated ablation, close to a main glissonian pedicle, is represented in Fig. 3. These ablations extended the time of hepatectomy by 15 ± 7 (range 9–27) minutes. This time included the probe position, the two ablations performed in 40s, clinical and ultrasound evaluation of the ablated areas.

Preparation and utilization of the device was safe and asepsis was not compromised. No damage occurred to neighbouring tissues during HIFU ablation. Two patients had a post-operative complication (pneumonia with pleural effusion associated with an anastomotic leakage, and a urinary tract infection with fever and mental confusion) not related to the ablations.

The procedure was well tolerated: there were no substantive changes in hemodynamic and respiratory parameters. The only change observed was in cardiac index and this was attributed to liver mobilization (including liberation from the inferior vena cava) and so unrelated to HIFU ablation.

On average, 88% (95% CI, 67 to100%) of the predefined areas (15 representative intra hepatic anatomical structures) were visualized with the IUSP. Our objective—of establishing that we would have been able to generate HIFU ablations in at least 80% of the liver volume—was met in five of six patients. In the two patients with previous portal vein embolization, right portal branches were difficult to visualize with the IUSP, but neither were they visualised with the ultrasound probe habitually used for intra-operative liver ultrasonography (Xario ultrasound system, SSA-660A model, Toshiba).

Given the achievement of the phase I objectives without HIFU-related adverse events, the study proceeded to phase IIa.

### Phase IIa

**Fig 4:**
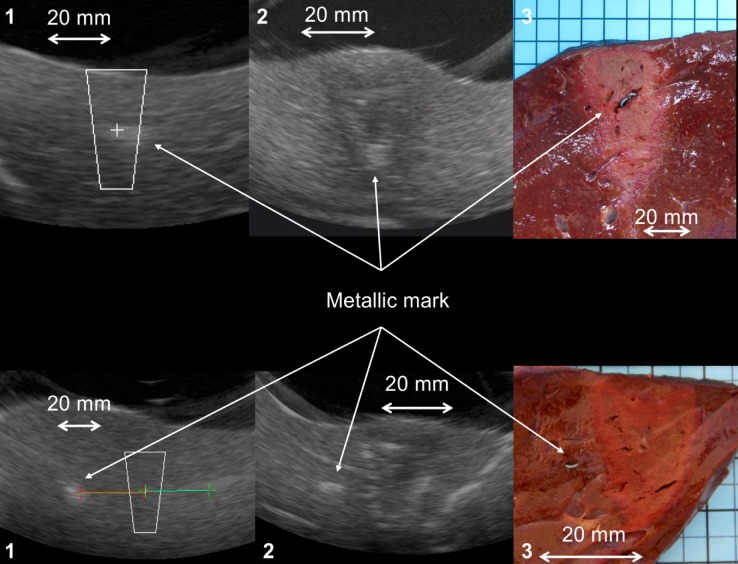
Proceedings of HIFU ablation. Example of HIFU ablations all around (top) or at a predefined distance (bottom) to a metallic mark in several steps: first target (1) then ablate (in 40 seconds) and visualize the ablated area with the integrated ultrasound imaging probe (2) and macroscopically (3).

During step 1, two metallic marks were implanted in each patient, one superficial and one deep. The intention was that the area targeted in a single HIFU ablation would include the mark. An example of ultrasound imaging and its correlation with macroscopic appearance is shown as Fig. 4 (top row of images). In the first three patients, gross pathological examination showed that in one of six ablations the mark had not been included. After reviewing the record of the procedure, we established that the metallic mark had been confused with a small Glissonian pedicle. Three more patients were then included (as required by the study design) and correctly targeted ablations were achieved around all six marks. Meeting the primary objective of step 1 allowed the steering committee to approve continuation of the study.

During step 2, HIFU ablations were made by positioning the closest theoretical ablation edge at a distance of 1 to 15 mm from the implanted metallic mark border, for an ideal distance of 7.5 mm. These values were defined taking into account the size of the HIFU ablations (20 mm in diameter), the size of the metallic mark (5 mm) and the field of view of IUSP. Two marks (one superficial and one deep) were implanted in each of three patients and two HIFU ablations were performed. The mean distance between the edge of the ablation and the mark was 7 ± 2.3 (4.3–9.8) mm. We therefore achieved our objective of demonstrating that we could ablate an area of tissue adjacent to an area that we wanted to preserve from damage. An example of ultrasonography imaging and its macroscopic correlate is shown in Fig. 4 (lower row of images).

The safety of our HIFU device was confirmed during Phase IIa. Manipulation of the device did not compromise asepsis and no adjacent organs were damaged during ablations. Two of 18 ablations discoloured but did not rupture Glisson’s capsule.

### Common secondary outcomes of Phase I and IIa

**Fig 5:**
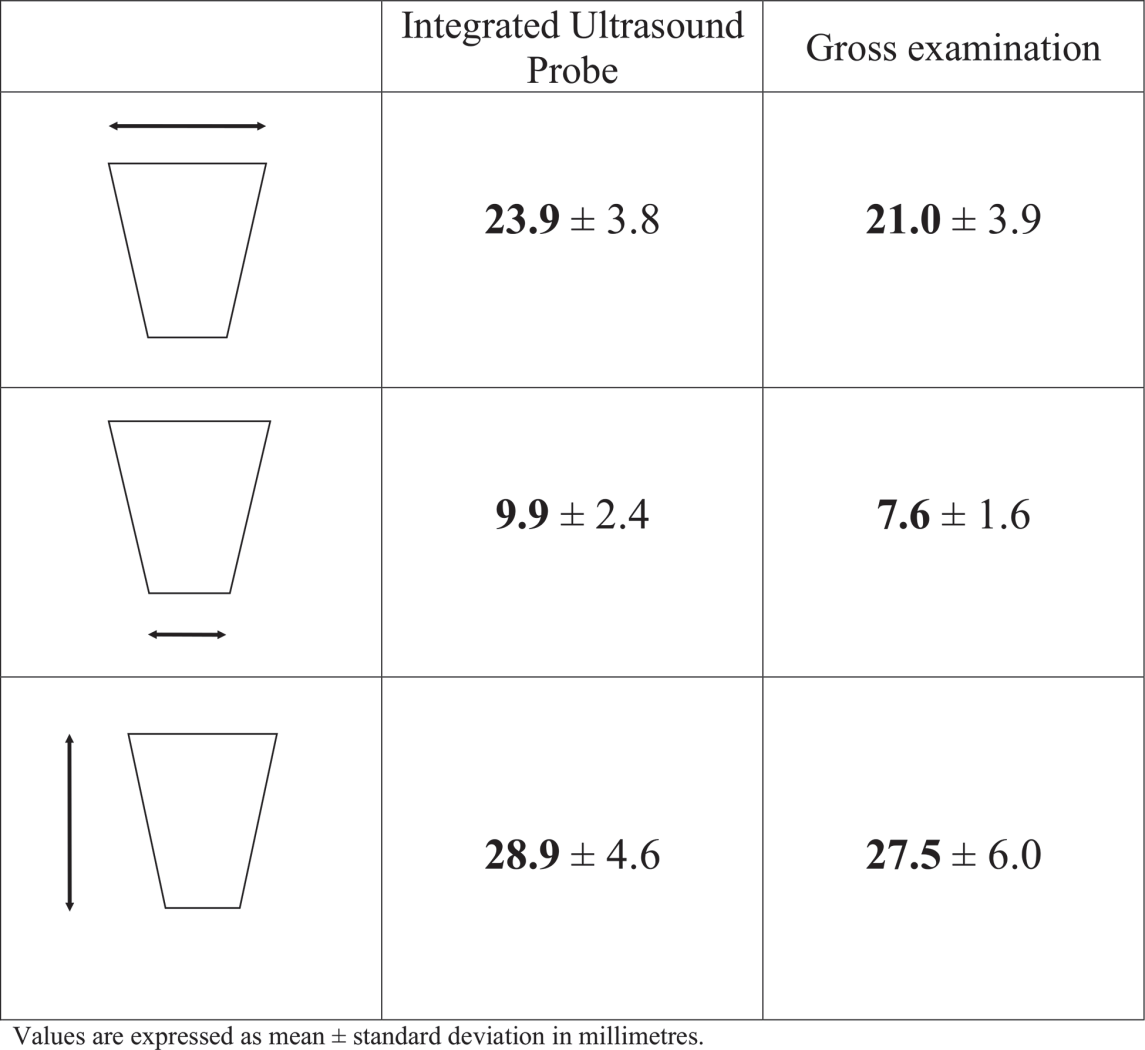
Measures of HIFU ablations obtained with the integrated ultrasound imaging probe and at gross pathological examination.

In total, thirty HIFU ablations were achieved in 15 patients. Our objective was to compare the dimensions measured on ultrasound (using the IUSP) with the dimensions observed macroscopically immediately after liver resection. On gross examination, all HIFU ablations were slightly cone-shaped with a mean length of 27.5 mm and a mean superior width of 21 mm. Fig. 5 summarizes the measures obtained on ultrasound images and on liver specimens. The rank correlation coefficient between macroscopic measurements and those based on intra-operative IUSP was 0.88, p<0.001 (95% CI, 0.82 to 0.91), which indicates a close and highly statistically significant correlation between these parameters (Fig. 3).

**Fig 6:**
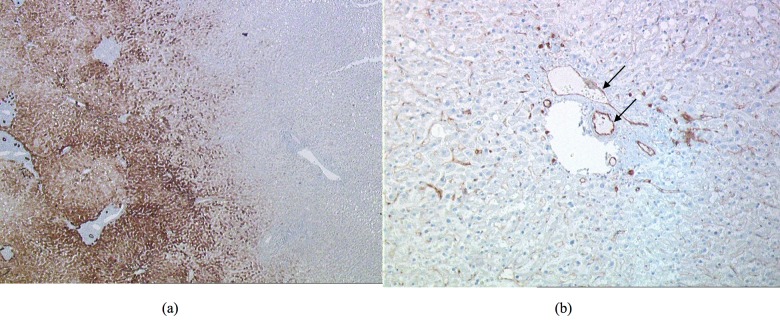
Histopathological examination after HIFU ablation. (a) Representative cytokeratin 18 staining. A loss of cytokeratin 18 postiviely stained hepatocytes can be seen in the ablated area, revealing a cellular alteration. Magnification X200. (b) Representative endothelial CD31 staining. A maintained CD31 positively stained endothelial cells (arrows) confirmed the preservation of the endothelial wall of vascular structures in the ablated area. Magnification X20.

As illustrated in Figs. 3 and 6, there was a sharp delimitation between the edges of the HIFU ablation and normal liver at both gross pathological and microscopic examination. All HIFU ablations were well delimited and surrounded by a halo of congestive tissues. The pathological examination confirmed homogeneous necrosis with all HIFU ablations, and this was particularly notable around vessels. Because of the limited time between HIFU ablation and hepatectomy, we were not able to observe evidence of apoptosis. However, the histological appearance of all HIFU ablations showed pronounced alterations in hepatocytes with decreased cytokeratin 18 and glutamine synthetase immunostaining and partially conserved expression of hepatocyte antigen (Fig. 6). Cytokeratin 7 immunostaining showed bile ducts marked out by altered cells. Küppfer cells, labelled by CD68-antibody, were reduced in number and had a retractile aspect. Oedema was found in extracellular matrix and vascular structures. Immunoreactivity for CD31 confirmed conservation of the endothelial barrier of portal vascular structures (Fig. 6). Expression of CD31 was diminished along the hepatic sinusoids.

## Discussion

The aim of the study was to confirm in a clinical context results previously obtained in a pig model regarding the feasibility, safety and efficacy of HIFU liver ablation. We report here the first use of intra-operative HIFU in patients with CLM. We aimed to perform at least two HIFU ablations in each patient, with a superficial ablation and a deeper ablation with the focal zone placed 1 cm deeper. In 15 patients, we safely produced a total of 30 HIFU ablations. Theses ablations were all generated within 40 seconds and on average measured 27.5 x 21 mm^2^.

In contrast to other modes of ablation using radiofrequency, cryotherapy or microwaves to achieve focal destruction, the HIFU technique does not require that a probe is implanted in the parenchyma. The study we report demonstrated that our HIFU device can be used in the operating room without compromising asepsis and that ablations can be achieved without damage to neighbouring organs and tissues.

The use of a completely extracorporeal HIFU device is clinically feasible, as demonstrated by the treatment of hepatocellular carcinoma [22,23]. However, only a small part of the liver (around 30%) is accessible using a non-invasive device, unless a partial rib resection is performed [24]. Moreover, the extracorporeal approach is associated with attenuation and complications due to phase aberration and liver movements which can produce skin burns and gastric lesions [25] [26]. Reducing the risk of these potentially important complications was among the reasons we chose the intra-operative approach. An open procedure seems also more appropriate than an extracorporeal technique for the treatment of CLM. It allows a better staging of malignancies since additional metastasis (hepatic or peritoneal) are discovered intraoperatively in 15–20% of patients, despite preoperative imaging [27,28]. To demonstrate the efficacy and safety of rapidly ablating large areas of liver, ablations were performed on healthy tissue. However, to ensure patient safety, we always took care to produce ablations close to metastases and in areas intended for resection. This preliminary work was regarded as essential before considering the routine use of our new intra-operative HIFU probe to treat CLM.

Use of integrated sonography gives us confidence in our ability to target HIFU precisely at the focal point intended for ablation. Large volume lesions were achieved with accuracy and in a short time. This is possible thanks to the geometry of the transducer. The large size of the device (10 cm diameter) does not limit ease of use. In 5 out of 6 cases, we were able to visualize about 90% of the entire liver and all major anatomical structures with the IUSP, even when the procedure was performed through a limited midline laparotomy.

Ablation dimensions on ultrasound were not identical to those on pathological examination. The observed differences were in great part explained by the imprecision in cutting fresh liver, even when care was taken to mark the axis of the ablations on the liver surface for easy reference in the pathology lab. Nevertheless, the area ablated by HIFU was clearly identified by the IUSP and the dimensions of the image correlated well with those from gross pathological examination. This is a major advantage over other ablative techniques: we were able to demonstrate the effectiveness of real-time US guidance to clearly determine the area to treat and to assess the precision of ablation and, potentially, the accuracy of treatment margins. In contrast, the hyperechoic image observed with ultrasonography after RFA overestimates the ablated area [29]. One important finding of the current study is that all HIFU ablations had the same size and shape even if generated around major vessels. Pathological examination confirmed the destruction of all forms of liver cell except the endothelial cells of major vessels, as we previously reported in the pig model [8]. These observations differ from those seen following RFA when the size and shape of lesions vary depending on the technique used and on tissue impedance [30].

Although this study advances our understanding of the potential of HIFU in treating CLM, there were some limitations. Since we targeted healthy liver, the efficacy of HIFU in ablating tumour tissue could not be demonstrated. Secondly, since we resected the ablated areas shortly after HIFU treatment, we were not able to investigate longer-lasting effects such as apoptosis or, of greater clinical significance, any post-operative complications that might emerge in patients whose ablated liver is kept in place.

The aim of this initial study was to obtain evidence of the safety, efficacy and precision of ablation achieved using this powerful new device. We thought it unethical to use the device to treat otherwise resectable metastases since incomplete ablation or poor targeting might have resulted in a patient losing the chance of potentially curative therapy. We therefore decided to perform the first assessments of this technique (Phase I and Phase IIa) using normal liver parenchyma. A patient was suggested for inclusion only if a major hepatectomy was planned and there was sufficient normal liver parenchyma between metastases. An example would be a right hepatectomy for CLM located in segments V, VII and VIII with a healthy segment VI. This feature of the study design meant that accrual was slow: only 15 patients were entered in 20 months. A second factor making for slow accrual was that patients taking part derived no benefit to themselves since the areas targeted for HIFU ablation were already scheduled for resection; and more than 30% of screened patients declined to be included.

This new HIFU device safely achieved large volume liver ablations in 40 seconds, with a precision of one to two millimetres under real-time monitoring. These initial clinical results justify the next step in clinical development (phase IIb) in which we will attempt HIFU ablation of small metastases (≤ 20 mm) and peri-lesional healthy liver. In this next phase, metastases will be wholly included in the area to be ablated with a planned safety margin of 5 mm. This will be obtained by juxtaposition of ablations as we previously described [13]. Immediately after HIFU treatment, hepatectomy will be performed to assess the efficacy and safety margin of ablations.

## Supporting Information

S1 Checklist TREND Checklist. (PDF)

S1 Ethics Initial ethics approval. (PDF)

S2 Ethics Amendment. (PDF)

S3 Ethics Ethics approval of amendment. (PDF)

S1 Protocol Trial protocol. (PDF)

S2 Protocol Trial protocol (french version). (PDF)
