# Correction: First Clinical Experience of Intra-Operative High Intensity Focused Ultrasound in Patients with Colorectal Liver Metastases: A Phase I-IIa Study

**DOI:** 10.1371/journal.pone.0123751

**Published:** 2015-03-30

**Authors:** The PLOS ONE Staff

The Funding statement in the PDF version of this article is incorrect. The publisher apologizes for the error. The correct Funding statement is: This work was supported by funding from the Cancéropôle Lyon Auvergne Rhône Alpes and the French NCI (under PHRC grant 12007 and Lyric grant INCa-DGOS-4664). The funders had no role in study design, data collection and analysis, decision to publish, or preparation of the manuscript.
