# Supplementary material for: First Clinical Experience of Intra-Operative High Intensity Focused Ultrasound in Patients with Colorectal Liver Metastases: A Phase I-IIa Study
Source: PLoS One. 2015 Feb 26;10(2):e0118212. doi: 10.1371/journal.pone.0118212 (PMC4342219; doi:10.1371/journal.pone.0118212)
Supplement: S1 Ethics — (PDF) [file pone.0118212.s002.pdf]

Comité de Protection des Personnes SUD-EST IV

Centre Léon Bérard – 28 rue Laennec – 69373 LYON CEDEX 08

Président : Dr. Daniel ESPINOUSE - Vice-président : Mme Carine DIVER
Trésorier : Dr. David PEROL

Madame Anne LEFRANC
Centre Léon Bérard
28, rue Laennec
69008 LYON

Lyon, le 24 septembre 2009

N° CPP : 09/070
Réf. de la délibération : A 09-205
N° ID RCB : 2009-A00779-48

Le Comité a été saisi le 10 septembre 2009 par le Centre Régional Léon Bérard d'une demande d'avis sur un projet de recherche intitulé :
"Evaluation, chez des patients nécessitant une chirurgie de résection de métastases hépatiques de cancers colorectaux, de l'utilisation per-opératoire d'ultrasons focalisés de haute intensité (HIFU) : faisabilité, innocuité, et capacité de ciblage des métastases" **- Protocole n° ET2009-068, version 0.0 du 10 août 2009;**
Documents d'information et de consentement, version du 10 août 2009 ;
Brochure pour l'investigateur :

1. **réponses aux exigences essentielles prototype MFOCUS de juillet 2009 ;**
2. **spécifications système MFOCUS de novembre 2008 ;**

Liste des investigateurs, d'août 2009 ;
L'investigateur coordonnateur est le Pr. Michel RIVOIRE, Département de Chirurgie, Centre Léon Bérard à Lyon (69).

Le Comité a examiné les informations relatives à ce projet au cours de sa séance du 22 septembre 2009.

Membres présents à la séance :
- Personnes qualifiées "Recherche Biomédicale" : Mme M. MONTANGE (titulaire), Dr. D. ESPINOUSE (titulaire), Dr. Pascale CONY-MAKHOUL (suppléante), Mme Nicole FALETTE (suppléante), Mme Raymonde MARAVAL-GAGET (suppléante).
- Médecin généraliste : Dr. M. LE GAL (titulaire, qualifiée en matière de biostatistique).
- Infirmiers : Mme Saléa PRADAT (suppléante).
- Personnes qualifiées "Ethiques" : Mme Christiane KAPITZ (titulaire), Mme F. TOURAINE-MOULIN (suppléante).
- Personnes qualifiées "Juridique" : Mme C. DIVER (titulaire).
- Représentants d'associations de malades et d'usagers de la santé : Dr. Denis AZOULAY (titulaire).

…/…

Secrétariat : Odette MARITAZ
☎ 04 78 78 27 61 - 🖷 04 78 78 28 58 – E-mail : maritazo@lyon.fnclcc.fr

Le Comité a adopté la délibération suivante :

AVIS FAVORABLE

Le seul traitement curatif actuel des métastases hépatiques des cancers colorectaux est la résection hépatique, accessible uniquement à une minorité de patients. Toutes les alternatives à la chirurgie testées jusqu'à maintenant n'ont pas été satisfaisantes. Il est donc indispensable de développer de nouvelles techniques de destruction tumorale, non invasive et complémentaire à la chirurgie.

La présente étude se propose d'utiliser en per-opératoire des ultrasons focalisés de haute densité (HIFU). Les travaux pré-cliniques ont démontré l'intérêt, la faisabilité et la tolérance clinique et biologique d'un tel traitement.
Il s'agit de tester ce dispositif médical dans une étude de phase I-II en trois parties chez des patients devant subir une résection des métastases hépatiques par hépatectomie. Ce procédé n'a encore jamais été utilisé chez l'homme dans cette indication.

La balance bénéfices/risques est positive puisqu'il s'agit d'utiliser une technique couplée à l'imagerie sur une partie du foie qui sera réséquée. Le seul risque pour le patient est une anesthésie générale plus longue, sans excéder 30 minutes.

Dr. Daniel ESPINOUSE,
Président de séance

Secrétariat : Odette MARITAZ
☎ 04 78 78 27 61 - 🖷 04 78 78 28 58 – E-mail : maritazo@lyon.fnclcc.fr
