# Supplementary material for: First Clinical Experience of Intra-Operative High Intensity Focused Ultrasound in Patients with Colorectal Liver Metastases: A Phase I-IIa Study
Source: PLoS One. 2015 Feb 26;10(2):e0118212. doi: 10.1371/journal.pone.0118212 (PMC4342219; doi:10.1371/journal.pone.0118212)
Supplement: S2 Ethics — (PDF) [file pone.0118212.s003.pdf]

RÉPUBLIQUE FRANÇAISE

afssaps

Agence française de sécurité sanitaire des produits de santé

Direction de l'évaluation des dispositifs médicaux
Unité essais cliniques (UEC)
Dossier suivi par Xavier WAGNER
Tél. +33 (0)1 55 87 38 02
Fax. : +33 (0)1 55 87 37 17
E-mail : dedim.dm@afssaps.sante.fr
N/Réf. : UEC/XavW/DA/2011-

Saint-Denis, le 11 FEV. 2011

Objet : Evaluation, chez des patients nécessitant une chirurgie de résection de métastases hépatiques de cancers colorectaux, de l'utilisation per-opératoire d'ultrasons focalisés de haute intensité : faisabilité, innocuité, et capacité de ciblage des métastases.
N° d'enregistrement : **2009-A00779-48 / MS1**

Madame,

Vous avez adressé à mes services une demande d'autorisation de modification substantielle pour la recherche biomédicale intitulée : « Evaluation, chez des patients nécessitant une chirurgie de résection de métastases hépatiques de cancers colorectaux, de l'utilisation per-opératoire d'ultrasons focalisés de haute intensité : faisabilité, innocuité, et capacité de ciblage des métastases. » et dont le numéro d'enregistrement est le 2009-A00779-48 / MS1.

Au vu du dossier reçu par l'Afssaps en date du 26 janvier 2011, j'autorise cette modification substantielle. En vertu des articles L. 1123-9 et R. 1123-35 du code de la santé publique, je vous rappelle toutefois qu'il vous appartient de demander l'avis, ou d'informer le comité de protection des personnes impliqué, si respectivement il s'agit, ou non, d'un élément du dossier initialement soumis à ce comité.

Les effets/évènements indésirables graves ainsi que les faits nouveaux susceptibles de porter atteinte à la sécurité des personnes sont à déclarer en vertu des articles L. 1123-10 du code de la santé publique. La fin de l'essai est également à déclarer en vertu de L. 1123-11 du code de la santé publique.

Je vous prie d'agréer, Madame, l'expression de mes salutations distinguées.

Centre Léon BERARD (CLB)
28 rue Laennec
69373 LYON CEDEX 08

A l'attention de Madame Anne LEFRANC

Cc : CPP Sud-Est IV

Le chef de Département surveillance du marché

Nicolas THEVENET
