# Supplementary material for: First Clinical Experience of Intra-Operative High Intensity Focused Ultrasound in Patients with Colorectal Liver Metastases: A Phase I-IIa Study
Source: PLoS One. 2015 Feb 26;10(2):e0118212. doi: 10.1371/journal.pone.0118212 (PMC4342219; doi:10.1371/journal.pone.0118212)
Supplement: S3 Ethics — (PDF) [file pone.0118212.s004.pdf]

Comité de Protection des Personnes SUD-EST IV

Centre Léon Bérard – 28 rue Laennec – 69373 LYON CEDEX 08

Président : Dr. Daniel ESPINOUSE - Vice-président : Mme Carine DIVER
Secrétaire général : Mme Odette MARITAZ - Trésorier : Dr. David PEROL

Madame Anne LEFRANC
Centre Léon Bérard
28, rue Laennec
69008 LYON

Lyon, le 13 janvier 2011

N° CPP : 09/070
Réf. de la délibération : A 11-12
N° ID RCB : 2009-A00779-48

Le Comité a été saisi le 10 janvier 2011 par le Centre Régional Léon Bérard d'une demande d'avis complémentaire sur un amendement n° 1 au projet de recherche intitulé :
"Evaluation, chez des patients nécessitant une chirurgie de résection de métastases hépatiques de cancers colorectaux, de l'utilisation per-opératoire d'ultrasons focalisés de haute intensité (HIFU) : faisabilité, innocuité, et capacité de ciblage des métastases" **- Protocole n° ET2009-068, version 2.0 du 9 janvier 2011;**
Documents d'information et de consentement, version du 9 janvier 2011 ;
Liste des investigateurs, d'août 2009 ;
L'investigateur coordonnateur est le Pr. Michel RIVOIRE, Département de Chirurgie, Centre Léon Bérard à Lyon (69).
Ce projet a précédemment fait l'objet d'un avis favorable du Comité le 24 septembre 2009.

Cet amendement n° 1 vise à mettre à jour le protocole de phase II en fonction des résultats de la phase I, notamment en ciblant les tirs HIFU par rapport à un repère stable métallique et en limitant le nombre de tirs à deux.

Le Comité a examiné les informations relatives à cet avis complémentaire lors de sa séance du 11 janvier 2011.

Membres présents à la séance :
- Personnes qualifiées "Recherche Biomédicale" : Mme M. MONTANGE (titulaire), Dr. D. PEROL (titulaire, qualifié en matière de biostatistique), Mme B. PIGNATELLI (titulaire), Dr. D. ESPINOUSE (titulaire), Mme N. FALETTE (suppléante), Mme R. MARAVAL-GAGET (suppléante).
- Médecin généraliste : Dr. M. LE GAL (titulaire).
- Pharmacien : Mme C. STAMM (titulaire).
- Infirmier : Mr. G. DUYCK (titulaire).
- Personnes qualifiées "Ethiques" : Mme C. KAPITZ (titulaire), Mme F. TOURAINE-MOULIN (suppléante).
- Personne qualifiée "Juridique" : Mme C. DIVER (titulaire).
- Représentant d'associations de malades et d'usagers de la santé : Dr. D. AZOULAY (titulaire).

Le Comité a adopté la délibération suivante :

AVIS FAVORABLE à l'amendement n° 1

Dr. Daniel ESPINOUSE,
Président de séance

Secrétariat : Odette MARITAZ
☎ 04 78 78 27 61 - 🖷 04 78 78 28 58 – E-mail : maritazo@lyon.fnclcc.fr
