# Supplementary material for: First Clinical Experience of Intra-Operative High Intensity Focused Ultrasound in Patients with Colorectal Liver Metastases: A Phase I-IIa Study
Source: PLoS One. 2015 Feb 26;10(2):e0118212. doi: 10.1371/journal.pone.0118212 (PMC4342219; doi:10.1371/journal.pone.0118212)
Supplement: S1 Protocol — (PDF) [file pone.0118212.s005.pdf]

CONTENTS

I. INTRODUCTION AND RATIONALE 8

I.1. SURGICAL RESECTION OF LIVER METASTASES IN COLORECTAL CANCER: CURRENT SITUATION 8
I.2. THERAPEUTICAL USE OF ULTRASOUNDS 8
I.3. DEVELOPMENT OF A TORIC HIFU TRANSDUCER FOR TREATMENT OF CLM 9
I.3.a. Preclinical study of HIFU treatment feasibility: analysis of a single hurt and strategies to extend necrosis 9
1.3. b. Preclinical study of HIFU treatments efficacy with a pseudotumoral pattern 10
I.3.c. Collaboration with the EDAP society – Development of a perioperative usable prototype in human being 11
I.4. EXPERIENCE OF THE TEAM IN THIS SUBJECT 11

II. OVERVIEW OF THE STUDY 12

II.1. TREATMENT INDICATION 12
II.2. STUDY DESIGN 12
II.3. BENEFIT / RISK RATIO 12
II.4. STUDY PROCEDURES 13
III.1. PHASE I 13
III.2. PHASE II 14
III.2.a. Phase IIa 15
III.2.b. Phase IIb 16

IV. METHODS 17

IV.1. POPULATION 17
IV.1.a. Inclusion criteria 17
IV.1.b. Non-inclusion criteria 17
IV.1.c. Specific inclusion criteria related to phase IIb 17
IV.2. STUDY PROCEDURE 18
IV.2.a. Prerequisites 18
IV.2.b. Inclusion 18
IV.2.c. Surgical procedures 18
IV.2.d. Postoperative follow-up 19
IV.3. MONITORING 20
IV.3.a. Common assessment to all phases 20
IV.3.b. Phase I 20
IV.3.c. Phase IIa 21
IV.3.d. Phase IIb 21
IV.4. EXCLUSION CRITERIA DURING THE STUDY 21
IV.5. PREMATURE END OF THE STUDY 21

V. SAFETY 22

V.1. NOTIFICATION OF ADVERSE EVENTS BY INVESTIGATORS 22
V.1.a. Notification of adverse events in the case report form 22
V.1.b. Notification of sever adverse events to the sponsor 22
V.1.c. Adverse events not to be reported immediately 22
V.1.d. Adverse events related to the pathology or the surgery 22
V.1.e. Expected adverse events – Definition of the benchmark document 22
V.2. ARCHIVING OF ADVERSE EVENTS STATEMENTS 23

VI. STATISTICAL METHODS 24

VI.1. SAMPLE SIZE AND STATISTICAL RULES 24
VI.1.a. Phase I 24
VI.1.b. Phase II 24
VI.2. DEFINITION OF THE STUDIED POPULATION 26
VI.3. DATA ANALYSIS 26

VII. EXPECTED OUTCOMES 26

VIII. STUDY MANAGEMENT AND COORDINATION 26

VIII.1. STEERING COMMITTEE 26
VIII.2. DATA COLLECTION 27
VIII.3. DATA REVIEW 27

VIII.4. DATA PROCESSING 27
VIII.5. STATISTICAL ANALYSIS 27
VIII.6. STUDY REPORT 27
VIII.7. PUBLICATIONS 27

IX. ETHICS 28

IX.1. DECISION-MAKING SERVICES 28
IX.1.a. CPP 28
IX.1.b. AFSSAPS 28
IX.1.c. CCTIRS et CNIL 28
IX.1.d. Insurance 28
IX.2. DATA OWNERSHIP AND PRIVACY 28
IX.3. AUDITS AND CONTROL 29
IX.3.1. Coordinating centre audit 29
IX.3.2. Health regulatory authorities controls 29

X. BIBLIOGRAPHY 30

ABBREVIATIONS LIST

- (AFSSAPS)-DEDIM Département d'Evaluation des Dispositifs Médicaux
- AE Adverse events
- AFSSAPS Agence Française de Sécurité Sanitaire des Produits de Santé
- BP Blood Pressure
- CCTIRS Comité Consultatif pour le Traitement de l'Information en matière de Recherche sur la Santé
- CF Cardiac Flow
- CLB Centre Léon Bérard
- CLM Colorectal Liver Metastases
- CNIL Commission Nationale Informatique et Liberté
- CNOR Certified Nurse Operating Room
- CPP Comité de Protection des Personnes
- CRA Clinical Research Assistant
- CRF Case Report Form
- CRT Clinical Research Technician
- CSA Clinical Study Assistant
- ESI Experimental Surgery Institute
- HIFU High Intensity Focused Ultrasound
- HR Heart Rate
- INSERM Institut Nationale de la Santé et de la Recherche Médicale
- MD Medical Device
- $PaCO_2$ Partial pressure of Carbon dioxide in arterial blood
- PAO_2 Partial pressure of Oxygen in arterial blood
- RPC Regional Pharmacovigilance Centre
- RR Respiratory Rate
- SAE Severe Adverse Events
- SpO_2 Saturation of Peripheral 0_2
- TCIA Target-Controlled IntraVenous Anaesthesia
- UBET Unité de Biostatistique et d'Evaluation des Thérapeutiques
- VT Tidal Volume
- WHO World Health Organization

FOREWORD

In this protocol the following terms will be used:

- "**Shootings**" means to deliver an ultrasound load through a HIFU transducer in order to cause a necrosis in a determined area.
- " **Hurt"** refers to the necrosed area generated by the use of HIFU

SYNOPSIS

SHORT TITLE

HIFU

TITLE

A phase I-II study to evaluate, in patients operated for a hepatic resection of colorectal liver metastases, a High Intensity Focused Ultrasound (HIFU) procedure: feasibility, safety and accuracy

PROTOCOL NUMBER

N° CLB : ET2009-068
N° ID RCB : 2009-A00779-48

SPONSOR

Centre Léon Bérard (CLB) - LYON

PRINCIPAL INVESTIGATOR

Pr Michel RIVOIRE – Surgery Department – CLB

COORDINATING CENTRE

Unité de Biostatistique et d'Evaluation des Thérapeutiques (UBET) – Département de Santé Publique (DSP) – CLB

INDICATION

Colorectal Liver Metastases (CLM) requiring surgery.

RATIONALE

In France, the ColoRectal Cancer (CRC) is the second cancer considering its incidence with about 36 000 new cases each year (source: French network of cancer registers). It sill have a poor prognosis causing nearly 16 000 deaths a year and the more often due to metastases.

Hepatic resection is the mainstay for CLM and remains to date the only curative treatment. However, only few patients (10 to 20 %) can benefit from it. Consequently, many techniques were developed and evaluated, such as focused radiofrequency or cryotherapy, in order to find out an alternative to the surgery of CLM. Nevertheless, these widespread techniques have many limitations: they do not enable reliable real-time monitoring, they are invasive, can only be applied to small tumors, imply long duration treatment and lead to high rate of local recurrence.

Therefore, it is necessary to develop a more accurate process to kill tumoral cells, which would not be size-limited nor invasive, in addition to the surgical treatment.

High Intensity Focused Ultrasound (HIFU) is a new approach, which enables to generate irreversible cell death through coagulative necrosis in a few seconds. There is no cooling effect of the perfusion because of the shortness of the phenomenon. Perioperative HIFU in CLM has never been evaluated clinically. The surgical team of the Centre Léon Bérard, in collaboration with the INSERM U556 one, has undertaken a research program on CLM treatment by HIFU. A new and very powerful device, without the previously listed limitations, has been developed. Preclinical studies revealed the interest, the feasibility and the safety of this process. These results enable to consider the implementation of a clinical trial.

STUDY DESIGN

Prospective, monocentric phase I/II study evaluating a surgical medical device (SMD)

MAIN ASSESSMENT CRITERIA

1st part; Phase I

- ✓ (Feasibility) Ability to perform shootings, supplementary duration of intervention < 30 minutes
- ✓ (Safety) Asepsis, absence of lesion of nearly tissues.
- ✓ (Tolerance) Preservation of hemodynamic and respiratory vital signs.

2nd part: Phase IIa

- ✓ (Accuracy) distance from the epicentre of the HIFU lesion to the mark previously positioned in the liver ≤ 5mm.

3rd part: Phase IIb

- ✓ (Efficacy) macro-lesion generated, including metastasis, in negative margins.

POPULATION

Inclusion criteria

- 18 years old or more patient,
- Affected of hepatic metastasis of a colorectal cancer,
- Who must undergo a hepatectomy by laparotomy with the aim of the resection of hepatic metastasis,
- ECOG performance status (PS) = 1,
- Mandatory affiliation to a health security insurance,
- Written informed consent.

Non-inclusion criteria

- Having already undergone a major hepatic surgery (more than three segments) or biliary major (context of major iterative hepatic surgery),
- Having already undergone a major abdominal surgery with the exception of a colorectal surgery for the treatment of its primitive tumor (the surgery of the gall-bladder by laparoscopy for the deadline upper to 6 months do not constitute a criterion of non inclusion),
- Unable to be followed during the duration of the study,
- Pregnant or breast-feeding woman (a pregnancy test must be negative at the time of the inclusion in the study for the women in age to procreate; a method of reliable contraception must be used during the duration of the study).

STUDY PROCEDURE

Inclusion

The patients will be included during the surgery preliminary visit, by the surgeon

Surgical Procedure

✓ **Settings**

During the first part of the intervention, once liver freed and indication of liver resection checked, prior to undertake any specific procedure regarding liver resection, the investigator surgeon will perform the shootings on the dedicated part of the liver.

✓ **Phase I**

The shootings will concern the healthy liver to remove, distant to the important vascular structures and metastases. Two HIFU shootings will be made on each patient: one on the surface of the liver and the other one in depth.

✓ **Phase IIa**

The shootings will concern healthy liver to remove, distant (step 1) then close (step 2) to the important vascular structures of the liver. Several shootings HIFU will be done on each patient (ideally 4 hurts, minimum 2 hurts).

✓ **Phase IIb**

Shootings will concern small liver metastases to be resected and will be juxtaposed on multiple plans, to generate a hurt containing the metastasis and guaranteeing sufficient safety margins. Several metastases can be treated in the same patient.

NB: After the HIFU shootings, there will be no difference regarding the hepatectomy: this one will be as identical as the one which would have been done in the absence of HIFU shootings.

Immediate following of the operation

The whole abdominal cavity will be explored to detect possible adverse effects.

The anatomopathological analysis will include, in addition, an analysis of each of the HIFU hurts.

Postoperative follow-up

Within the framework of this study, the patients will be followed during 30 days after the surgical operation.

STATISTICAL METHODS

32 to 38 patients will be included (phase I : 6 patients, phase Iia : 6 to 12 patients, phase IIb : about 20 patients).

For each phase, analyses will be achieved in the selected population to evaluate the clinical feasibility, the tolerance and the efficacy in CLM treated by HIFU. This population concerns all the patients included in the study and who underwent a hepatectomy. The data analysis will be mainly

descriptive.
An interim analysis will be given to the Steering committee in charge of the study.
This Committee will meet to make the necessary decisions as for the continuation of the study according to its progress, in agreement with the Sponsor.

Key steps identification

**Start of enrolment** :	first quarter 2010
**Inclusion period** :	18 months
**Patients follow-up** :	30 days after the end of the surgery
**Estimation of the study duration** :	19 months

I. Introduction and rationale

I.1. Surgical resection of liver metastases in colorectal cancer: current situation

Colorectal liver metastases (CLM) are a major public health issue (16 000 deaths each year in France) (1). Despite all improvements achieved regarding chemotherapy (2) and other therapies based on the use of physical agents (radiofrequency and cryotherapy) (3;4), complete resection of CLM offers the only potential curative approach - A 5-year survival rate after liver resection is ranged from 20 to 35%. However, only few patients (10 to 20 %) can benefit from it. Mainly because of the number, the location, and the size of the liver metastases or because the insufficient remaining functional liver volume contraindicates the surgery (5;6).

Consequently, many alternatives to surgery of CLM have been evaluated. Currently, focused radiofrequency, cryotherapy, micro-waves and laser hyperthermia are the main local treatments used in CLM (7). Radiofrequency, and cryotherapy, which are more common, led to an increase of the number of patients treated for CLM with promising results. These techniques have yet several limitations. They do not enable reliable real-time monitoring (per operative treatment modification cannot be done with CT-scan) and require intraparenchymal introduction of a probe. Moreover, only small hepatic volume can be targeted. Finally, a 2-year recurrence rate is important. It ranges from 30% to 40% for cryotherapy and nearly 15% (2% -60%) for radiofrequency (8, 9).

Therefore, it is necessary to develop a more accurate process to kill tumoral cells which would not be size-limited nor invasive, in addition to surgical treatment. The main goal is to make curative surgery possible for a larger population and to reduce the rate of liver recurrences after resection. To this extent, focused tumoral treatments provide a good efficacy and spare more significantly healthy liver.

I.2. Therapeutical use of ultrasounds

HIFU are mechanical waves generated by an ultrasound transducer. A focused approach enables a sudden and accurate energy absorption in a distant but precisely defined targeted area. This induces a temperature increase going from 40 to 60°C in a few seconds and generates irreversible cell death through coagulative necrosis in an ellipsoidal shape with a 15 mm long axis and a diameter of 20 mm. There is no cooling effect of the perfusion because of the shortness of the phenomenon. The main asset of the technique is the use of non-ionizing waves which allows high focusing without hazardous effects on surrounding tissues. Plus it leads to almost no adverse effects. Thus, for about two decades, therapeutical ultrasounds implementations (hyperthermia, HIFU, lithotripsy) have been rising and explored in numerous ways: extracorporeal, percutaneous, transrectal, laparoscopic, transoesophagal, perioperative. Preclinical and clinical studies using these treatments concerned various pathologies such as glaucoma (10 ;11), localized prostate carcinoma (12 ;13), benign prostate hypertrophy (14;15), or other organs like bladder (16), breast (17), oesophagus (18), liver (19), uterine fibroma (20) or even brain (21). Because of the different ways to reach organs to be treated and depending on the way chosen for the treatment, many specific transducer technologies were developed. Nowadays, therapeutic ultrasounds appear as a great supplementary option to surgery among treatments using physical agents. Despite all these elements, HIFU technologies are to be improved concerning CLM treatment. It is in particular necessary to decrease the time of intervention because the elementary hurt HIFU is ellipsoïdale and of small dimensions. A treatment by HIFU consists in juxtaposing in a precise way (generally by a movement automated by the probe of treatment) these elementary hurts until containing the tumor targeted with a margin of healthy tissues.

At the present time a treatment of prostate cancer by HIFU takes 149 minutes (22). Moreover, a real-time imaging device must be combined to HIFU to see precisely the treated area after each shooting.

Extracorporeal treatments in primitive liver cancer using HIFU are currently tested by Chinese and English teams but come up with questions that are not yet answered: bones stopping waves propagation, air filled spaces and targeting difficulty related to breathing. Furthermore, it is hard to reach liver in depth.

For all these reasons the surgical team of the CLB, in collaboration with the NIHMR U556 one, has undertaken a preclinical research program on CLM treatment by HIFU. A new and very powerful device enabling large hurts in the liver, has been developed.

I.3. Development of a toric hifu transducer for treatment of CLM

Cf. biblio (23;24)

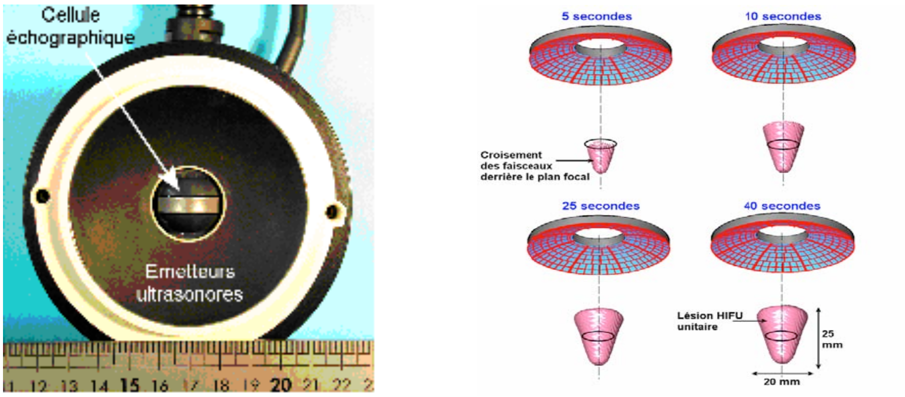

Figure 1 : HIFU probe.

A perioperative ultrasound medical probe in treatment of CLM with HIFU has been developed and received at a preclinical phase in a porcine model. 7 cm^3 large (20 mm of diameter and 25 mm of long axis) unitary conical resections were done in 40 seconds with a crossed toric transducer which is an efficient result considering the time consumed and the volume treated. A hole in the middle of the medical device (MD) enables to add an echographic probe to localize and control liver metastases treatment. Characteristics of the generated hurts were studied on the models and in *in vitro* and *in vivo* preclinical studies.

I.3.a. Preclinical study of HIFU treatment feasibility: analysis of a single hurt and strategies to extend necrosis

Cf. biblio (25;26)

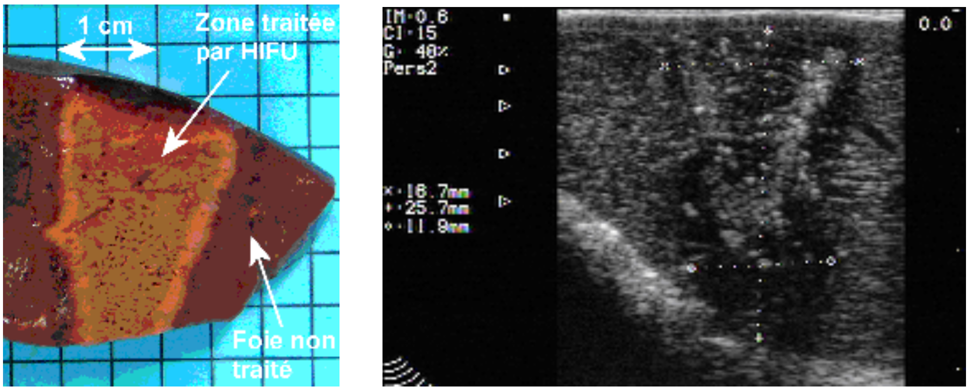

(a) **(B)**

Figure 2: (a) Elementary HIFU hurt generated in 40 seconds

(b) Echographic aspect of elementary HIFU hurt.

One of the most important things, concerning the CLM treatment, is the accuracy of targeting. In standard surgery or with a therapy using physical agents (radiofrequency, cryotherapy) the treatment is considered properly done when a tumor is killed with negative geometrical margins. The elementary hurt generated by the toric HIFU transducer in treatment of CLM was thoroughly studied in preclinical phase *in vivo* in pig liver. Its geometrical features (Figure 2a) and the absence of cooling effect on perfusion are really relevant to cure CLM taking into account its rich vascularization. The perioperative manual use of toric HIFU transducer prototype gave accurate localized treatment in liver, thanks to the echographic guidance provided by the combined probe. Since both localization and necrosis in liver tissues were controlled, the proposed HIFU treatment seemed to be more convenient. The hurts are homogenous and have, microscopically or macroscopically, well defined borders.

Generated hurts are easily visible on echographic pictures (Figure 2b) which makes possible to place side by side several elementary cone-shaped hurts to extend the size of the generated necrosis (Figure 3). The treatment is non-invasive to the organ and is well tolerated.

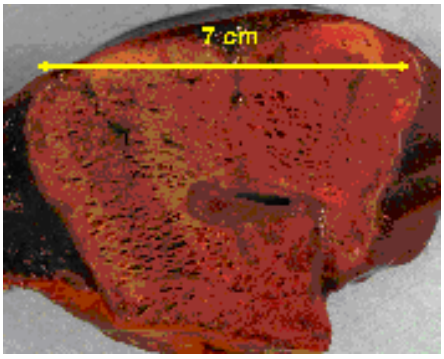

Figure 3: Juxtaposition of 6 elementary hurts

1.3. b. Preclinical study of HIFU treatments efficacy with a pseudotumoral pattern

Cf. biblio (27)

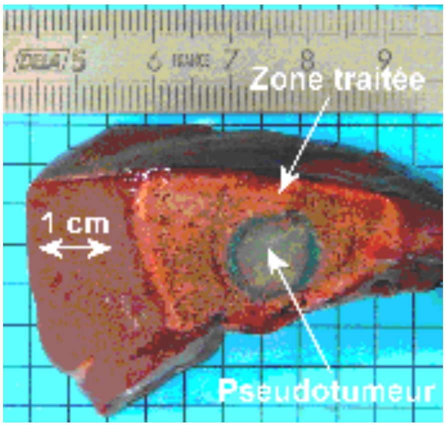

Figure 4 : PseudoTumor HIFU

The objective of these studies was the preclinical evaluation of the accuracy of the toric HIFU transducer prototype in treatment of CLM, using a pseudotumoral pattern implanted in a pig liver. This pattern is identifiable in both anathomopathology and echographic imaging, and can therefore estimate the accuracy of the volume targeted by HIFU (Figure 4). Hurts were done ensuring negative margin all around the pseudotumor and in all directions.

Studied shootings on a pseudotumoral pattern measured precisely the accuracy of the treatment using juxtaposed elementary hurts to generate large and well targeted liver lesions. Through all these

preclinical studies, clearly appears the interest of using a HIFU transducer manually in a perioperative procedure.

I.3.c. Collaboration with the EDAP society – Development of a perioperative usable prototype in human being

Previously described studies were undertaken with experimental prototype developed by the unit 556 of INSERM in collaboration with the EDAP society which has the world leadership in HIFU prostate cancer treatment. In this indication, was developed Ablatherm® which is now worldwide spread. The work previously described was funded through a research program "Preuve de Concept" proposed by the "Canceropôle Lyon Auvergne Rhône-Alpes" (CLARA). Positive results led EDAP society, with the help of "Oséo-ANVAR", to create a new MD usable in human being: MFOCUS® prototype

I.4. Experience of the team in this subject

The CLB surgical team's proficiency in curing liver metastases, especially CLM, is well known. This is shown by an important activity of liver metastases resections (80 to 100 operations/year), by the development of focused technologies to kill tumors in addition to or as an option to surgery, by the involvement in a large number of clinical trials with frequent collaborations with medical oncology teams (28)

For 3 years since then, in collaboration with the U556 of INSERM, a research program has been developed on HIFU as treatment of CLM (24). The experience gathered through *in vitro* and *in vivo* preclinical experimentations found in national and international publications makes it today possible to conceive a rigorously led study to evaluate the feasibility, tolerance and efficacy of this technique.

Thanks to those results and the support provided by contributors, a clinical trial using this new therapeutic procedure is now conceivable in order to evaluate its performances in liver metastases treatment, which is the main point of the protocol.

It will be the first time in human being, and the first time in the whole world that a perioperative HIFU technique is assessed in treatment of liver metastases in addition to surgery.

II. Overview of the study

II.1. Treatment indication

Treatment of CLM.

II.2. Study design

This is a phase I/II, prospective, monocentric (CLB), first time evaluation in human being of a surgical MD without CE mark.

II.3. Benefit / risk ratio

The principal objective of the study is to confirm the results given in the pig model in human liver. It is important to remind that former experiments were well tolerated.
The study participants cannot expect any personal benefit from their participation.
However, to avoid any problem, it has been decided to test it in patient undergoing a curative operation for CLM. The surgical procedure to cure these patients' candidates to a liver resection will be totally identical to the one they would have undergone if not included in the study. The only difference will concern HIFU shootings on the healthy or peritumoral liver (depending on the study phase). These shootings will be performed, on liver to be resected, only after ultimate checking of indication and immediately before the resection phase. Thus, there is no risk to generate HIFU hurts on a healthy liver part that would not have to be resected.
The duration of the procedure of HIFU shootings will have to prolong the surgical operation of no more than 30 minutes. (Phase I main assessment criteria).
The principal potential risk to which could be exposed the patients of the study would be a hurt of surrounding organs secondary to HIFU shootings. This risk will be, of course, estimated after each shooting and this all through the study. Safety measure will be taken with particular care to avoid any accident: compresses will be put under the targeted area to limit risks of ultrasound propagation, and the shootings axis will not point towards noble intra-abdominal organs.
The post operative follow-up period will last 30 days, which seems to be enough time to report possible late AE. In that case, total care will be given until total remission or at least stabilization of adverse events.
Besides, the patients' enrolment will be divided into steps including from 2 to 4 participants (depending on the study phase). The decision to proceed to next steps will be taken by an independent Steering committee after considering all the study results. Naturally, the study could end prematurely due to SAE or accident.
Generally speaking, if the results of this study appear to be clinically relevant, the development of this technique could be a huge improvement in treatment of CLM, by providing non-invasive surgery for the considered organ and to extend this treatment to other patients who cannot benefit from hepatectomy (too large liver volume to resect, metastases localization, etc) or developing new extracorporeal treatment processes…

II.4. Study procedures

III. Objectives and measurable assessment criteria

Objectives are divided into two parts as following:

III.1. Phase I

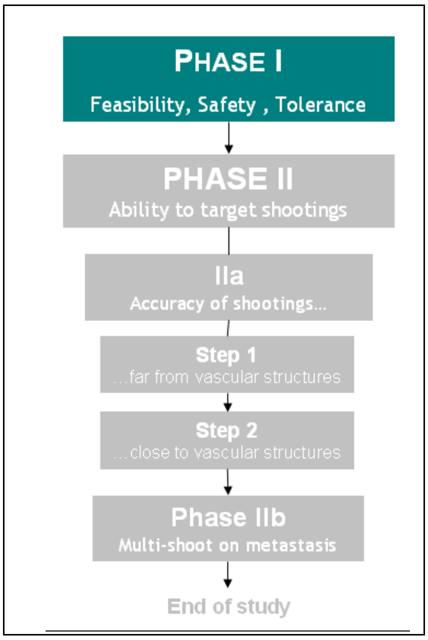

✓ **Primary objectives**

To estimate:

- Hifu shootings **feasibility**,
  ➯ *Through* :
  1) Ability to shoot :
     - On the surface of the liver : in this case the hurt will start from the Glisson capsule and be extended to the subjacent parenchyma,

     and

     - In DEPTH: In this case the hurt will be located in deeper parenchyma, at least 10 mm deeper than surface hurts.
  2) The supplementary duration of intervention for 2 shootings given in minutes includes:
     a. time to initialize the MD in operating room;
     b. probe conditioning with acoustic coupling liquid;
     c. time to define the target (x2) ;
     d. time to deliver a shooting (x2).

     The overtime caused must not exceed 30 minutes.

     NB : Step a. and b. above-mentioned can be done in parallel to surgery. As far as they do not increase the duration of the surgical operation, they will not be taken into account. Same for the subsidiary research acts such as implanting marks.

- **Safety** using the MD,
  ➯ *Through* :
  1) Capability to use the MD following requested asepsis procedures ,
  2) No evidence of hurt on peripheral tissues (Glisson capsule on the opposite side of the HIFU shootings entrance area, retro-peritoneal, retro-hepatic tissues and diaphragm).

     NB : it is possible that the Glisson capsule ultrasound hurts on the entrance area, that will be notified but not considered as a failure.

- HIFU shootings **tolerance**,
  ➯ *Through:* Ability to keep vital signs stable (hemodynamic[1], respiratory[2], body temperature) during shooting phase and the following 5 minutes.

[1] Hemodynamic vital signs : heart rate (HR), blood pressure (BP), heart flow (HF), by measuring the area under the curve on the Vigleo (N°3) monitor.

A significant alteration of vital signs will be defined as, at least, a 10% modification of one of these parameters compared to its value prior shootings.

✓ **Secondary objectives**

To determine :

- **Probe ergonomics** and feasibility of **area targeting**,
  ↳ *Through :* Ability to adjust the probe position to different liver segments and patient physical structure. The aim is to be free to generate a HIFU hurt in at least 80 % of the total liver volume.
- Feasibility to set a mark (mark to estimate shootings accuracy in phase IIa-step 1),
  ↳ *Through* : Possibility to set a stationary mark, at given depth, ecographically detectable,
- Feasibility to spot a specific vessel (used to estimate shootings targeting accuracy in phase IIa-step 2),
  ↳ *Through* : Possibility to spot ecographically a previously Patent Blue or Methylene blue marked vessel.
- **Each step duration** to set the MD (initialization, conditioning, targeting, shooting – cf. supra),
  ↳ *Through* : Mean duration to achieve each step expressed in minutes.
- Feasibility of real-time monitoring of the HIFU impact using an echograph combined with the MD,
  ↳ *Through:* Possibility to locate HIFU hurt shape, to identify and to describe its outline (diameters, depth, volume etc.) during perioperative echography.
- Feasibility of **hurt early anatomopathological description** ,
  ↳ *Through:* Possibility to locate HIFU hurt shape, to identify and to describe its limits (diameters, depth, volume, etc. – en mm) during anatomopathological analysis

  NB: anatomopathological procedures will be standardized in this phase.
- Feasibility to **measure distance between the mark / marked vessel and the hurt limits** during anatomopathological analysis,
  ↳ *Through:* Precise measure given in mm by the anatomopathologist.

III.2. Phase II

[2] Respiratory vital signs : saturation of Peripheral 0_2 ($Sp0_2$) *or* partial pressure of oxygen (PAO_2 – samples will be taken prior shootings and during the ending phase of evaluation, that is to say during the 5 minutes following the last shootings, on an arterial catheter) partial pressure of carbon dioxide in arterial blood ($PaC0_2$ – identical sampling procedure as for PAO_2)

III.2.a. Phase IIa

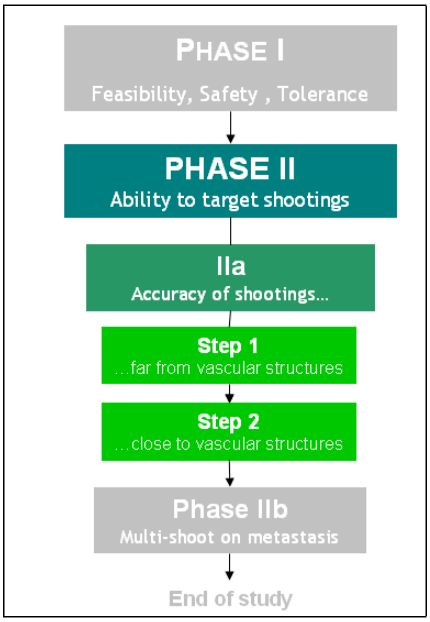

✓ **Primary objective**

To estimate :

- **Accuracy of shootings** on a precise area (step 1) then on a zone to be spared (step 2),
  ➯ *THROUGH:* both the distance between the hurt epicentre generated by hifu and a mark (step 1) and between the hurt limit and a vessel border previously marked (step 2). These sizes will be measured in mm during anatomopathological exam.

✓ **Secondary objectives**

To estimate :

- **Safety** using the MD,
  ➯ *Through:* No evidence of hurt on peripheral tissues (Glisson capsule on the opposite side of the HIFU shootings entrance area, retro-peritoneal, retro-hepatic tissues and diaphragm).

  NB: it is possible that the Glisson capsule ultrasound hurts on the entrance area that will be notified but not considered as a failure.

- HIFU shootings **tolerance**,
  ➯ *Through:* Ability to keep vital signs stable (hemodynamic, respiratory, body temperature) during shooting phase and the following 5 minutes.

- **Correlation between hurt perioperative echographic and postoperative macroscopic**, measures,
  ➯ *Through:* hurt dimensions comparison (diameters, depth, volume, etc. – in mm), blind-measured, with echography in a first time and in a second time, right after the resection, in anatomopathology.

III.2.b. Phase IIb

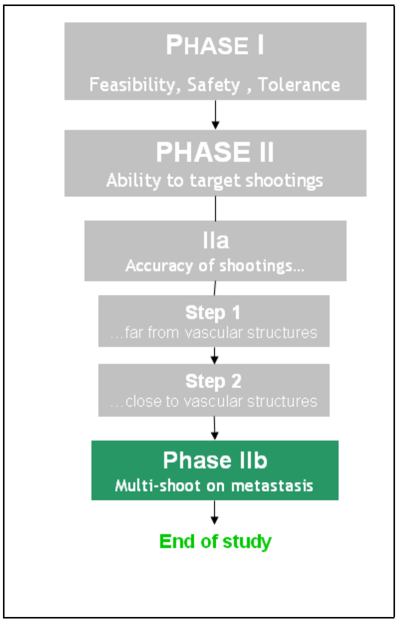

✓ **Primary objective**

To estimate :

- Possibility, by **juxtaposing elementary HIFU hurts in metastases** to be resected, to generate global hurts containing metastases and guaranteeing sufficient safety margin in healthy liver.
  ➯ *through :*
  1) Possibility of number of ≤ 15 shootings,
  2) The hifu hurt generated must encompass the metastasis and guarantee a safety margin ≥ 5 mm from any point (anatomopathological analysis).

✓ **Secondary objectives**

To estimate :

- **Safety** using the MD,
  ➯ *Through:* No evidence of hurt on peripheral tissues (Glisson capsule on the opposite side of the HIFU shootings entrance area, retro-peritoneal, retro-hepatic tissues and diaphragm).

  NB: it is possible that the Glisson capsule ultrasound hurts on the entrance area that will be notified but not considered as a failure
- HIFU shootings **tolerance**,
  ➯ *Through:* Ability to keep vital signs stable (hemodynamic, respiratory, body temperature) during shooting phase and the following 5 minutes.

IV. Methods

IV.1. Population

Patients complying with the following criteria:

IV.1.a. Inclusion criteria

- 18 years old or more patient,
- Affected of hepatic metastasis of a colorectal cancer,
- Who must undergo a hepatectomy by laparotomy with the aim of the resection of hepatic metastasis,
- ECOG performance status (PS) = 1,
- Mandatory affiliation to a health security insurance,
- Written informed consent.

IV.1.b. Non-inclusion criteria

- Having already undergone a major hepatic surgery (more than three segments) or biliary major (context of major iterative hepatic surgery),
- Having already undergone a major abdominal surgery with the exception of a colorectal surgery for the treatment of its primitive tumor (the surgery of the gall-bladder by laparoscopy for the deadline upper to 6 months do not constitute a criterion of not inclusion),
- Unable to be followed during the duration of the study,
- Pregnant or breast-feeding woman (a pregnancy test must be negative at the time of the inclusion in the study for the women in age to procreate; a method of reliable contraception must be used during the duration of the study).

IV.1.c. Specific inclusion criteria related to phase IIb

- Having one or more liver metastasi(e)s ≤ 2 cm, to be resected by hepatectomy

IV.2. Study procedure

IV.2.a. Prerequisites

The investigator team (investigator surgeon and National Certified Nurse Operating Room (CNOR)) that may act for the present study will have been taught principles of use of the MD by the qualified staff of the EDAP society, prior to the study start.

IV.2.b. Inclusion

The patients will be included during the surgery preliminary visit, by the surgeon (during the month preceding the operation). Once an eligible patient is identified, the investigator will inform him or her about the study, and will give him or her a written informed consent to be signed.

IV.2.c. Surgical procedures

Once liver fully freed and indication of liver resection checked, prior to undertake any specific procedure regarding liver resection, the investigator surgeon will perform the shootings on the dedicated part of the liver.

✓ **<u>Anaesthesia procedure</u>**
A standardized anaesthesia procedure will be defined for all patients included in the study.
Shootings will start only once full sedation is achieved. Except in vital emergency cases, no anaesthesia parameter will be modified during the evaluation phase (from first hurt targeting until 5 minutes after the last hurt).

✓ **<u>Initialization of the MD and probe preparation</u>**
The initialization of the MD in the operating room and the probe preparation will be done by previously trained CNOR, in parallel to the operation and as far as possible without consequence on the surgical procedure duration.

The whole echographic imaging will be recorded on a DVD.

✓ **<u>Patient's preparation</u>**
Vascular access will be given by a central venous catheter and an arterial catheter (procedure not related to the study). Protection compresses will be put under the targeted area to limit risks of ultrasound propagation, and hurt of any nearby organs. Abdominal cavity will be filled with a 37°C saline solution to improve the acoustic transmission between the HIFU transducer and liver. The HIFU probe will be in contact with the liver surface through an ultrasounds coupling liquid. From the user interface and with the help of the combined echographic probe, the target of treatment will be defined.
NB: In order to limit as far as possible risks to damage nearby organs, shootings axis will not point towards noble intra abdominal organs.

✓ **<u>Shootings process</u>**
⇨ **Phase I**
The shootings will concern the healthy liver to remove, distant to the important vascular structures and metastases. Two HIFU shootings will be made on each patient:

- One hurt on the surface starting from the Glisson capsule and extending to subjacent hepatic parenchyma.
- One hurt at given depth, at least 10 mm deeper than the hurt done on the surface

NB: The shooting depth will determined for each patient at the beginning of the operation, considering preoperative exams (scans, etc.), size and location of the liver part to be resected and physical structure of the patient.

⇨ **Phase IIa**

The shootings will concern healthy liver to be resected, distant (step 1) then close (step 2) to the important vascular structures of the liver. Several shootings HIFU will be made on each patient (ideally 4 hurts, minimum 2 hurts)

Step 1

Each mark will be spotted by a stationary mark set at a given depth (1st hurt on liver surface, 2nd hurt at depth of 40±5 mm, 3rd hurt at 20±5 mm, 4th hurt at 60±5 mm). Shootings will be performed by superimposing the theorical hurt epicentre and the mark (the mark will have to be included in the generated hurt)

Step 2

Vascular structures will be colored in Patent Blue or Methylene Blue and will work as a mark.
Shootings will be performed by positioning the closest theorical hurt limits to a vessel at a distance of 5 to 10 mm from the vessel border.

⇨ **Phase IIb**

Shootings will concern small metastases (≤ 20 mm) and peri lesional healthy liver. They will be juxtaposed on multiple plans in order to generate a large hurt containing the metastasis targeted and guaranteeing a sufficient safety margin in healthy liver.
Several metastases can be treated in the same patient

✓ **Immediate following of the intervention**

The whole abdominal cavity will be explored to detect possible adverse effects, especially burns, involving adjacent organs to liver.

Once HIFU delivered the hepatectomy procedure will be totally identical to the one that would have been done if patient would not have received HIFU shootings.

The anatomopathological analysis will include, in addition, an analysis of each of the HIFU hurts.

IV.2.d. Postoperative follow-up

All patients included will be followed in accordance with the guidelines for patients operated for CLM. Within the framework of this study, the patients will be followed during 30 days after the surgical operation.

IV.3. Monitoring

IV.3.a. Common assessment to all phases

	INCLUSION	PERIOPERATIVE	POSTOPERATIVE	30 JOURS POST OPERATION
☐ WRITTEN CONSENT (SIGNED AND DATED)	☐			
☐ PREGNANCY TEST & IF NEEDED PRESCRIPTION OF A RELIABLE MEAN OF CONTRACEPTION (WOMEN OF CHILDBEARGIN AGE)	☐			
☐ PERIOPERATIVE CT-SCAN (WITH PRECISE METASTASES SIZES IN PHASE IIB)	☐			
☐ CLINICAL CHECK-UP	☐			
☐ NO EVIDENCE OF HURT IN NEARBY ORGANS		☐	☐	☐
☐ HEMODYNAMIC VITAL SIGNS		☐		
☐ RESPIRATORY VITAL SIGNS		☐		
☐ BODY TEMPERATURE		☐		

IV.3.b. Phase I

	INCLUSION	PERIOPERATIVE	POSTOPERATIVE	30 JOURS POST OPERATION
☐ SHOOTINGS FEASIBILITY<br>• ON SURFACE<br>• IN DEPTH		 ☐<br>☐		
☐ DURATION<br>• OF OVERTIME NEEDED TO PERFORM 2 HIFU SHOOTINGS<br>• OF EACH SHOOTINGS STEP (INITIALIZATION, PROBE CONDITIONING, TARGETING, SHOOTING		 ☐<br>☐		
☐ ASEPSIS		☐		
☐ POSSIBILITY TO GENERATE A HIFU HURT IN AT LEAST 80% OF THE TOTAL LIVER		☐		
☐ POSSIBILITY TO :<br>• SET A STATIONARY MARK<br>• MARK A VESSEL WITH PATENT BLUE OR METHYLENE BLUE		 ☐<br>☐	 ☐<br>☐	
☐ FEASIBILITY OF REAL-TIME MONITORING OF THE HIFU IMPACT USING AN ECHOGRAPH COMBINED WITH THE MD		☐		
☐ FEASIBILITY OF HURTWITH EARLY ANATOMOPATHOLOGICAL DESCRIPTION			☐	
☐ FEASIBILITY TO MEASURE DISTANCE BETWEEN THE MARK / MARKED VESSEL AND THE HURT LIMITS DURING ANATOMOPATHOLOGICAL ANALYSIS			☐	

IV.3.c. Phase IIa

	INCLUSION	PERIOPERATIVE	POSTOPERATIVE	30 JOURS POST OPERATION
☐ DISTANCE BETWEEN<br>• HURT EPICENTRE / MARK (STEP 1)<br>• HURT LIMITS AND MARKED VESSEL BORDERS (STEP 2)			☐<br>☐	
☐ CORRELATION BETWEEN HURT MEASURES TROUGH<br>• ECHOGRAPHY COUPLED TO THE MD<br>• ANATOMOPHATHOLOGICAL ANALYSIS RIGTH AFTER THE OPERATION		☐	☐	

IV.3.d. Phase IIb

	INCLUSION	PERIOPERATIVE	POSTOPERATIVE	30 JOURS POST OPERATION
☐ SHOOTINGS AMOUNT / METASTASIS		☐		
☐ ANATOMOPATHOLOGICAL ANALYSIS			☐	

IV.4. Exclusion criteria during the study

A patient can be excluded of the study for one or several reasons:

- On sponsor or investigator decision,
- Withdrawal of his or her consent, or opposition to receive treatment,
- Inability to continue study (impossibility to carry out the hepatectomy, perioperative contraindication, intercurrent disease, worsening of clinical state, administrative reasons…)
- No news of the patient (lost to follow up),
- Patient's death.

Except in the last 2 cases, a final evaluation must be done, establishing the reasons why patient has to be dropped out.

IV.5. Premature end of the study

The study can end prematurely due to administrative reasons, or if new elements come up and reveal an unbalanced benefit / risks ratio, and/or on sponsor or statutory authorities decision.
If the study ends prematurely or has to be suspended, the sponsor will immediately inform the investigators, statutory authorities and the CPP why. Whatever happens during the study, a follow-up has to be achieved by the investigator

V. SAFETY

The CLB is represented by the Lyon Regional Pharmacovigilance Centre (RPC) for the safety in clinical trials for which it is the sponsor.

V.1. Notification of adverse events by investigators

V.1.a. Notification of adverse events in the case report form

Any intercurrent clinical or biological event, either severe or not, happening at anytime of the study, being related to research or not, must be written in the dedicated part of the case report form (CRF)
All intercurrent events, of any severity, must be followed by investigator until total remission or at least stabilization of the adverse event.

V.1.b. Notification of sever adverse events to the sponsor

The investigator will immediately inform the sponsor about all Severe Adverse Events (SAE) occurring in patients included or among users of the MD during the study period (except for those mentioned hereunder), in a notification form, the SAE being considered imputable to research or not. The initial notification must be promptly followed by one or several supplementary detailed statement(s).

Any SAE occuring in patients included must be stated from the date of the written consent until 30 days after surgery (being the end of the planned follow-up period), without time limits if investigator thinks that the event is likely to be related to the experimentation.

V.1.c. Adverse events not to be reported immediately

Non severe events: Less than 24 hours hospitalisation.
Events not related to MD experimentation: planned hospitalisation prior to the study start.
NB: If isolated, these events do not require to be immediately notified to sponsor, but need to be reported in the CRF.

V.1.d. Adverse events related to the pathology or the surgery

SAE related to colorectal metastatic cancer: digestive haemorrhage associated with anastomisis, perianastomtic abscess, digestive fistula, persisting post operative ileus…
Complications specific to liver metastases surgery: Bleeding from the hepatectomy resection, clinical or biological liver insufficiency (increased bilirubin above 50µmol/L and decreased prothrombine beneath 50 % after the fifth day post operation), ascites, icterus, portal hypertension signs, persistent biliary fistula…

V.1.e. Expected adverse events – Definition of the benchmark document

Are considered as expectable AE, any AE appearing in the benchmark document of the experimental MD. This MD does not own the CE mark. The benchmark is made of documents related to MFOCUS® experimental MD:

- First preclinical experimentations report with toric HIFU transducer, June 2009,
- MFOCUS® prototype handbook of EDP 800 548 A version, July,
- Liver metastases treated by HIFU - risks analysis – EDP 050 project, July 2009.

With all these elements, potential identified risks associated with HIFU treatment and therefore expectable AE are:

- For patients : contamination risk during probe setting,
- For patients : Burns of adjacent organs during HIFU shootings,
- For the medical team / user: injury risk resulting from a fall of the MD support (2 kg).

Besides during preclinical phase in animal, one peritonitis as a consequence of a biliary leak has been noticed. NB: The framework of this study does not include the treatment of this area.

V.2. Archiving of adverse events statements

The original statements of AE done by investigators and checked by the Clinical Research Assistant (CRA) during monitoring visits to make sure that the elements of the clinical file are conform to what has been faxed to the Pharmacovigilance Centre.
AE statements and related documents are filed by the sponsor following predefined procedures.
A copy is kept by the investigator in the CRF.

VI. STATISTICAL METHODS

VI.1. Sample size and statistical rules

This study plans to include 32 to 38 patients:

VI.1.a. Phase I

A group of 6 patients will be included, in a sequential analysis divided into 2 steps following Lee's criteria (29). This analysis aims to stop prematurely the study if a failure rate > 15 % is observed.

<u>STATISTICAL RULES :</u>

A failure is defined by at least one of the following situations:

- Overtime caused by shootings for 2 hurts exceeding 30 minutes,
- Impossibility to perform shootings on surface and/or in depth,
- Failing to respect requested asepsis procedures while setting the MD,
- Accidental hurt of nearby organs,
- Significant alteration of vital signs of at least 10 % from its original value prior to the shootings.

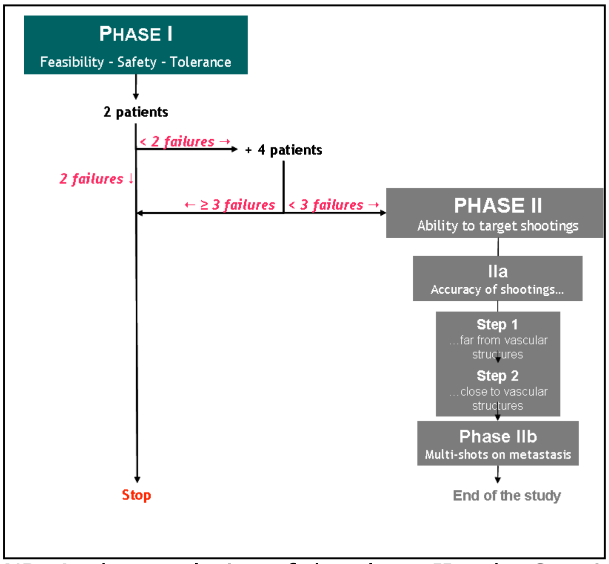

To proceed to phase II will require to have fulfilled the following rule:

- 1st analysis after 2 patients:
  - If < 2 failures are noticed ➔ continuation of the study and inclusion of 4 new patients,
  - If 2 failures the study is stopped.
- 2nd analysis after 6 patients:
  - If < 3 failures are noticed ➔ continuation of the study and beginning of phase II (with Steering Committee's consent),
  - If ≥ 3 failures ➔ study is stopped.

<u>NB:</u> At the conclusion of the phase IIa, the Steering committee of the study will meet to examine all the brought together data, validate or not the criteria of continuation of the study and define more exactly the methodology of the phase IIb.

VI.1.b. Phase II

✓ **<u>Phase IIa</u>**

A group of 6 to 12 patients will be included in 2 sequential steps with 3 to 6 patients in each. This number of selected patients has been defined by a classic dose escalation method used in clinical trials. The goal is to bring to light an insufficient accuracy of HIFU shootings if this event arises with a high frequency. Based on the binomial distribution, groups of respectively 3 and 6 patients allow a 90 % probability to get at least one patient receiving an unacceptable shooting (inaccuracy), if this event occurs respectively in at least 54 % and 32 % of the cases.

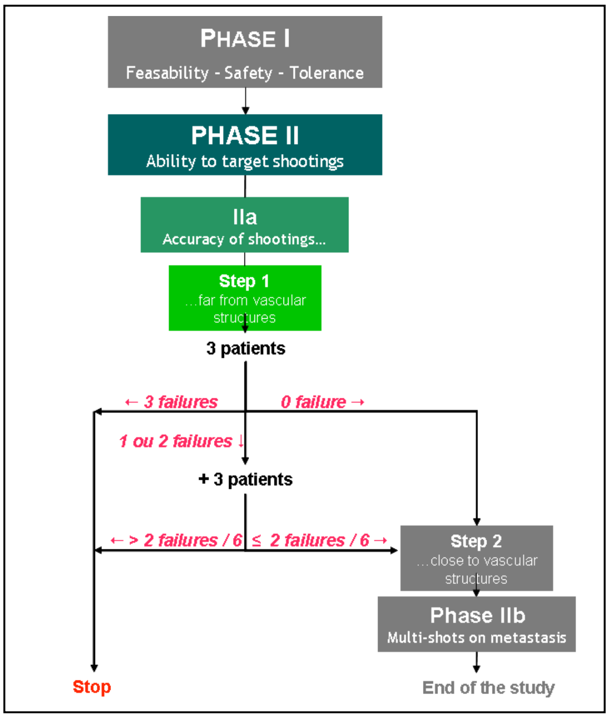

- **1st step** (3 to 6 patients) :
A success is defined for one patient in a given amount of shootings (2 ≤ n ≤ 4), if the mean distance between the closest hurt limit to the mark and the mark itself is < 5 mm. Otherwise, the event will be considered as a failure.

3 patients will be included in the 1st step:

  - If no failure is noticed ➔ proceed to step 2;
  - If 1 or 2 failure is(are) noticed 3 more patient are included in first step.
    ↳If > 2 failures out of 6 ➔ the study is stopped,
    ↳If ≤ 2 failures out of 6 patients ➔ proceed to 2nd step
  - If 3 failures are noticed ➔ the study is stopped.

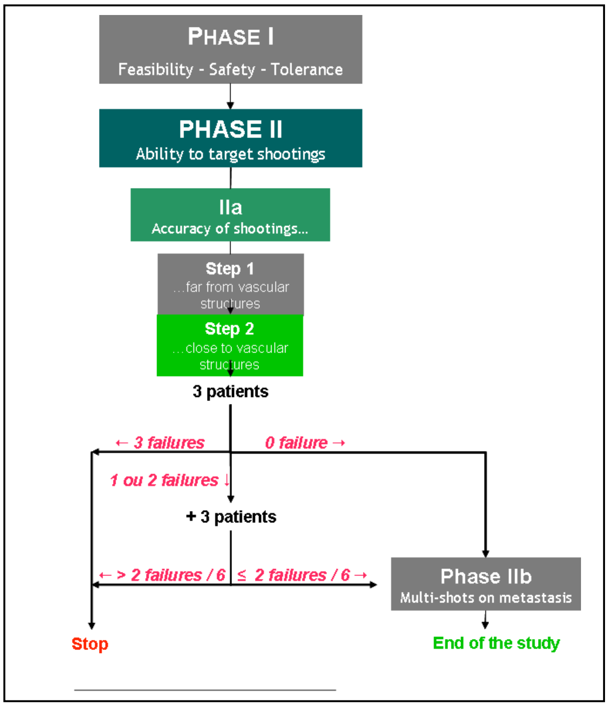

- **2nd Step** (3 to 6 patients) :
A success is defined for one patient in a given amount of shootings (2 ≤ n ≤ 4), if the mean distance between the closest hurt limit to the vessel mark and the vessel it-self is ≥ 5 mm and < 10 mm. Otherwise, the event will be considered as a failure.

3 patients will be included in the 1st step:

  - If no failure is noticed ➔ proceed to phase IIb;
  - If 1 or 2 failure is(are) noticed, 3 more patient are included in first.
    ↳If > 2 failures out of 6 ➔ the study is stopped,
    ↳If ≤ 2 failures out of 6 patients ➔ proceed to phase IIb,
  - If 3 failures are noticed ➔ the study is stopped.

<u>NB:</u> At the conclusion of the phase IIa, the Steering committee of the study will meet to examine all the brought together data, validate or not the criteria of continuation of the study and define more exactly the methodology of the phase IIb.

✓ **Phase IIb**

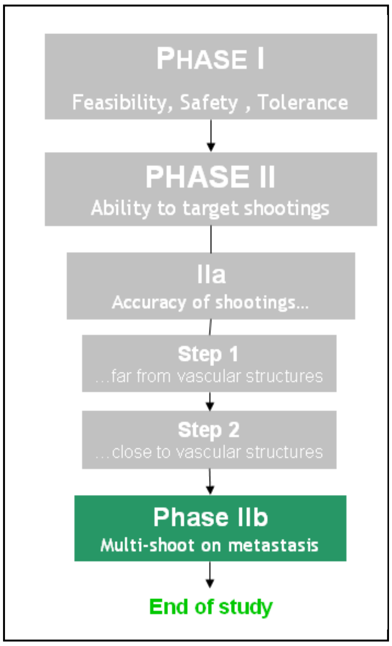

A group of 20 patients will be included in this phase. This number has been defined as following:

- The statistical unit for analysis is the metastasis,
- Fleming-A'Hern's method was formerly developed for phase II studies without steps (30) and has been used to determine the number of metastasis to target. A success is defined by a hurt encompassing the metastasis and guaranteeing safety margin ≥ 5 mm from any point, within a maximum of 15 shots.

The next hypotheses are used, in which P is the success probability:

- **p_0 = 0,70** is the superior limit value of success rate which, if met, would mean that HIFU are inefficient on metastases to be resected and peri lesional healthy liver.
- **p_1 = 0,90** is the inferior limit value of success rate which, if met, would mean that HIFU are efficient to treat metastases to be resected and peri lesional healthy liver.

Given a 5 % alpha risk and an 80 % statistical power, 28 metastases will have to be targeted by HIFU to conclude unilaterally. Assuming that about 1,5 metastases will be targeted on each included patient, that is to say nearly 20 patients.
A count of successes ≥ 24 enables to reject H_0 in favour of H_1.

VI.2. Definition of the studied population

In each phase, the primary and secondary endpoints analyses will concern the population assessable for clinical feasibility, tolerance and accuracy to target CLM with HIFU. This population will consist in all patients included and which could have undergone a hepatectomy. Only those which could not have will be dropped out.

VI.3. Data analysis

Data analysis will be mainly descriptive. Qualitative data will be described by their frequency distribution and associated percentage with a 95 % confidence interval. Quantitative data will be described by the number of observations, mean, standard deviation, median, minimum and maximum values.

VII. Expected outcomes

Bringing to light, the feasibility, the tolerance, and the accuracy to target CLM by HIFU will be an essential step before using this new technique in curative or extra corporeal applications.

VIII. Study Management and coordination

VIII.1. Steering committee

A steering committee will be established prior to the study start.
An interim analysis will be given to the Steering committee in charge of the study. As the study goes along, they will have to take a decision as regards its continuation, with the agreement of the sponsor.

VIII.2. Data collection

All the data brought together by the investigator, assisted by CNOR and the Clinical Study Assistant (CSA) chosen for this task will be reported in the CRF for each patient. The forms will be regularly sent to the Coordinating Centre.

NB: To carry out properly this study, procedures and instructions given to fill in the CRF must be followed.

VIII.3. Data review

A Clinical Research Assistant from the Coordinating Centre will be in charge of the quality control of the data during monitoring visits.
NB: During phase I, the quality control will focus on the validation of the eligible criteria and the SAE notification.

VIII.4. Data processing

All data collected in the specific forms will be numerically managed by a Clinical Research Technician (TRC) from the Coordinating Centre. A coherence check-list will be defined and applied to all numerised forms. This control will lead to rectifications queries checked by the investigators. It will be active until no more rectification query is generated.

VIII.5. Statistical analysis

Statistical analysis will be achieved at the end of the study, once the database is frozen (when rectifications are done and rechecked).

VIII.6. Study report

The report of the final analysis will be transferred to project and statistical managers and to the coordinating investigator at the latest 6 months after the study closure (freezing of database).

VIII.7. Publications

The coordinating investigator is bound to publish the study results. No publication (abstract, poster, oral or written work…) can be done without his or her agreement.

PUBLICATION RULES:

Any publication or communication (oral or written) will be commonly decided by the coordinating investigator and the Coordinating Centre Manager (UBET) and will follow the international guidelines (http://www.icmje.org/).

The listed authors, who highly contributed to protocol elaboration, study progression, and results edition, will be the first signers, namely:

- The investigator(s) who enrolled and followed patients will all be cited. If necessary the names order will be determined upon the number of patients included by authors,
- The UBET staff who participated to elaboration of the study protocol, those who will be in charge of data analysis and who helped to edit scientific articles,
- The INSERM team who made this study possible,

NB:
- The CLB will be cited as the study Sponsor and acknowledged,
- The EDAP-TMS society will be cited in the acknowledgements list,
- Other sponsors will be included in the acknowledgements list as well.

IX. ETHICS

The study will be conducted in agreement with:

- the ethical principles of the Declaration of Helsinki,
- Good Clinical Practice guidelines issued by the International Conference on harmonization (ICH-E6, 17/07/96),
- European Directive (2001/20/EC) of April 4th 2001 on clinical trial conduct,
- French Public Health Law (n°2004-806) of August 9th 2004,
- French law "Loi Informatique et Libertés n°78-17" of January 6th 1978 modified by the law n°2004-801 of August 6th 2004 related to the protection of individuals with regard to the processing of personal data,
- French bioethics Law n°2004-800 of August 6th 2004.

IX.1. Decision-making services

IX.1.a. CPP

This study received a favourable approval by the CPP Sud-Est IV on the 2009/09/22.

IX.1.b. AFSSAPS

This study received an authorization by the AFSSAPS on the (AFSSAPS-DEDIM) 2009/09/23.

IX.1.c. CCTIRS et CNIL

- This study already has the authorization of the Comité Consultatif sur le Traitement de l'Information en matière de Recherche dans le domaine de la Santé (CCTIRS) – Commission Nationale de l'Informatique et des Libertés (CNIL) : Record number: 00.S.018 for CCTIRS, and 00-1142 for CNIL

IX.1.d. Insurance

In accordance with the French public health law (n°2004-806) of August 9th 2004 (decree 2006-477 of April 26th 2006), the Sponsor must subscribe an insurance to cover financially any accident in relation with the present procedure.

IX.2. Data ownership and privacy

Patients's data collected on CRF during the trial will be registered in an anonymous way and the patients will only be identified by the patient number, and by his/her initials if also required.
The sponsor is the owner of all collected data and reports.
All relative information to the experimental device linked with this study, not previously published, will be considered as confidential and not subject to disclosure.
Study documents provided by the sponsor (protocols, investigators' brochures, CRF and other material) will be stored appropriately to ensure their confidentiality.
Each study collaborator is bound by professional secrecy (R.5120 article, Code de Santé Publique)..

IX.3. Audits and control

IX.3.1. Coordinating centre audit

On-site monitoring by a panel of experts may also be performed on behalf of the coordinating center.
The investigator (or his/her deputy) agrees to cooperate with the monitors for the duration of the visit, and to give them full access to all laboratory data and technical devices, as well as to patients' records. The verification of the data must be done by direct inspection of source documents.
Patient anonymity will be protected and all personal information made available for inspection will be handled in strictest confidence.

IX.3.2. Health regulatory authorities controls

The investigator must allow the inspection of the study-related records by the regulatory authorities, it could concern any of the following points:

- general organization of the study,
- qualification of the involved people,
- quality of equipments,
- informed consent forms,
- approval by independent ethics committee,
- storage and distribution of medical device,
- Management of the study (collected data, etc.),
- Archiving of study-related records.

X. BIBLIOGRAPHY

Reference List

1. Manfredi S, Lepage C, Hatem C, Coatmeur O, Faivre J, Bouvier AM. Epidemiology and management of liver metastases from colorectal cancer. *Ann.Surg.* 2006;244:254-9.

2. Bismuth H, Adam R, Levi F, Farabos C, Waechter F, Castaing D et al. Resection of nonresectable liver metastases from colorectal cancer after neoadjuvant chemotherapy. *Ann.Surg.* 1996;224:509-20.

3. Mulier S. Local recurrence after hepatic radiofrequency coagulation: multivariate meta-analysis and review of contributing factors. 2005.

4. Rivoire M. Combination of neoadjuvant chemotherapy with cryotherapy and surgical resection for the treatment of unresectable liver metastases from colorectal carcinoma. 2002.

5. Antoniou A, Lovegrove RE, Tilney HS, Heriot AG, John TG, Rees M et al. Meta-analysis of clinical outcome after first and second liver resection for colorectal metastases. *Surgery* 2007;141:9-18.

6. Pawlik TM, Schulick RD, Choti MA. Expanding criteria for resectability of colorectal liver metastases. *Oncologist.* 2008;13:51-64.

7. Garcea G, Lloyd TD, Aylott C, Maddern G, Berry DP. The emergent role of focal liver ablation techniques in the treatment of primary and secondary liver tumours. *Eur.J.Cancer* 2003;39:2150-64.

8. Mulier S. Local recurrence after hepatic radiofrequency coagulation: multivariate meta-analysis and review of contributing factors. 2005.

9. Seifert JK. Indicators of recurrence following cryotherapy for hepatic metastases from colorectal cancer. 1999.

10. Coleman DJ, Lizzi FL, Driller J, Rosado AL, Burgess SE, Torpey JH et al. Therapeutic ultrasound in the treatment of glaucoma. II. Clinical applications. *Ophthalmology* 1985;92:347-53.

11. Valtot F, Kopel J, Petit E, Moulin F, Haut J. [Treatment of refractory glaucoma with high density focused ultrasonics]. *J.Fr.Ophtalmol.* 1995;18:3-12.

12. Chapelon JY, Margonari J, Vernier F, Gorry F, Ecochard R, Gelet A. In vivo effects of high-intensity ultrasound on prostatic adenocarcinoma Dunning R3327. *Cancer Res.* 1992;52:6353-7.

13. Poissonnier L, Chapelon JY, Rouviere O, Curiel L, Bouvier R, Martin X et al. Control of prostate cancer by transrectal HIFU in 227 patients. *Eur.Urol.* 2007;51:381-7.

14. Kinsey AM, Diederich CJ, Rieke V, Nau WH, Pauly KB, Bouley D et al. Transurethral ultrasound applicators with dynamic multi-sector control for prostate thermal therapy: in vivo evaluation under MR guidance. *Med.Phys.* 2008;35:2081-93.

15. Madersbacher S, Pedevilla M, Vingers L, Susani M, Marberger M. Effect of high-intensity focused ultrasound on human prostate cancer in vivo. *Cancer Res.* 1995;55:3346-51.

16. Vallancien G, Harouni M, Guillonneau B, Veillon B, Bougaran J. Ablation of superficial bladder tumors with focused extracorporeal pyrotherapy. *Urology* 1996;47:204-7.

17. Wu F, Wang ZB, Cao YD, Zhu XQ, Zhu H, Chen WZ et al. "Wide local ablation" of localized breast cancer using high intensity focused ultrasound. *J.Surg.Oncol.* 2007;96:130-6.

18. Melodelima D, Prat F, Fritsch J, Theillere Y, Cathignol D. Treatment of esophageal tumors using high intensity intraluminal ultrasound: first clinical results. *J.Transl.Med.* 2008;6:28.

19. Wu F, Wang ZB, Chen WZ, Zhu H, Bai J, Zou JZ et al. Extracorporeal high intensity focused ultrasound ablation in the treatment of patients with large hepatocellular carcinoma. *Ann.Surg.Oncol.* 2004;11:1061-9.

20. Vaezy S, Fujimoto VY, Walker C, Martin RW, Chi EY, Crum LA. Treatment of uterine fibroid tumors in a nude mouse model using high-intensity focused ultrasound. *Am.J.Obstet.Gynecol.* 2000;183:6-11.

21. Marquet F, Pernot M, Aubry JF, Montaldo G, Marsac L, Tanter M et al. Non-invasive transcranial ultrasound therapy based on a 3D CT scan: protocol validation and in vitro results. *Phys.Med.Biol.* 2009;54:2597-613.

22. Uchida T, Ohkusa H, Nagata Y, Hyodo T, Satoh T, Irie A. Treatment of localized prostate cancer using high-intensity focused ultrasound. *BJU.Int.* 2006;97:56-61.

23. Melodelima, D. Toric HIFU transducer for large thermal ablation. 2007.
Ref Type: Conference Proceeding

24. Melodelima D, N'Djin WA, Parmentier H, Chesnais S, Rivoire M, Chapelon JY. Ultrasound surgery with a toric transducer allows the treatment of large volumes over short periods of time. *Applied Physics Letters* 2007;91:**193901**.

25. Melodelima D. Thermal ablation by high-intensity-focused ultrasound using a toroid transducer increases the coagulated volume. Results of animal experiments. 2009.

26. Parmentier H. High-Intensity Focused Ultrasound Ablation for the Treatment of Colorectal Liver Metastases During an Open Procedure: Study on the Pig. 2009.

27. N'Djin WA. Utility of a tumor-mimic model for the evaluation of the accuracy of HIFU treatments. Results of in vitro experiments in the liver. 2008.

28. Rivoire M. Combination of neoadjuvant chemotherapy with cryotherapy and surgical resection for the treatment of unresectable liver metastases from colorectal carcinoma. 2002.

29. Lee YJ, Wesley RA. Statistical contributions to phase II trials in cancer: interpretation, analysis and design. *Semin.Oncol.* 1981;8:403-16.

30. A'Hern RP. Sample size tables for exact single-stage phase II designs. *Stat.Med.* 2001;20:859-66.
