# Supplementary material for: First Clinical Experience of Intra-Operative High Intensity Focused Ultrasound in Patients with Colorectal Liver Metastases: A Phase I-IIa Study
Source: PLoS One. 2015 Feb 26;10(2):e0118212. doi: 10.1371/journal.pone.0118212 (PMC4342219; doi:10.1371/journal.pone.0118212)
Supplement: S2 Protocol — (PDF) [file pone.0118212.s006.pdf]

PROMOTEUR

Centre Léon Bérard (CLB)

28 rue Laennec - 69373 Lyon Cedex 08

Protocole HIFU

Version n°4.0 - 15/07/2013

EVALUATION, CHEZ DES PATIENTS NECESSITANT UNE CHIRURGIE DE RESECTION DE METASTASES HEPATIQUES, DE L'UTILISATION PER-OPERATOIRE D'ULTRASONS FOCALISES DE HAUTE INTENSITE (HIFU) : FAISABILITE, INNOCUITE ET CAPACITE DE CIBLAGE DES METASTASES

N° protocole Promoteur : ET2009-068 / **N° ID-RCB :** 2009-A00779-48

INVESTIGATEUR COORDONNATEUR

Pr Michel Rivoire - Département de Chirurgie – CLB

☏ : 04.78.78.28.34 🖶 : 04.78.78.28.90 💻 : michel.rivoire@lyon.unicancer.fr

CENTRE DE COORDINATION

Direction de la Recherche Clinique et de l'Innovation (DRCI) – CLB

☏ : 04.78.78.27.52 🖶 : 04.78.78.27.15

Autorisé par l'Agence Nationale de Sécurité du Médicament (ANSM) le 23/09/2009

Ayant reçu l'avis favorable du Comité de Protection des Personnes (CPP) Sud Est IV le 22/09/2009

REFERENTS

✓ Le référent sur les **aspects cliniques** est représenté par :

Pr Michel RIVOIRE - Investigateur coordonnateur - Département de Chirurgie - CLB
☏ : 04.78.78.28.34 - 🖶 : 04.78.78.28.90 - 💻 : michel.rivoire@lyon.unicancer.fr

✓ Les référents sur les **aspects techniques** sont représentés par :

Dr David MELODELIMA – Chercheur - Unité 556 - INSERM
☏ : 04.72.68.19.30 - 🖶 : 04.72.68.19.31 - 💻 : david.melodelima@inserm.fr

M. Nicolas GUILLEN – Ingénieur biomédical - EDAP-TMS
☏ : 04.72.15.31.98 - 🖶 : 04.72.15.31.51 - 💻 : nguillen@edap-tms.com

✓ Les référents sur les **aspects méthodologiques** sont représentés par :

Dr David PEROL – Coordonnateur médical et scientifique - DRCI CLB
☏ : 04.78.78.27.52 - 💻 : david.perol@lyon.unicancer.fr

Sylvie CHABAUD – Responsable du Pôle Biostatistiques - DRCI - CLB
☏ : 04.78.78.27.98 - 💻 : sylvie.chabaud@lyon.unicancer.fr

COMITE DE PILOTAGE

✓ Le Comité de pilotage est composé de :

M. Emmanuel BLANC	EDAP - TMS
Dr Jean-Yves CHAPELON	Unité 556 – INSERM
Dr Arnaud DE LA FOUCHARDIERE	Département d'anatomopathologie - CLB
Dr Dominique ELIAS	Département de chirurgie générale - Institut Gustave Roussy
DRCIPr. Frédéric MARCHAL	Département de chirurgie oncologique - Centre Alexis Vautrin
Dr David MELODELIMA	Unité 556 - INSERM
Dr David PÉROL	DRCI - CLB
Pr Michel RIVOIRE	Département de Chirurgie – CLB
Dr Hervé ROSAY	Département d'anesthésie – CLB
Pr Olivier ROUVIERE	Département de radiologie - Hôpital Edouard Herriot
Pr. Alain SEZEUR	Département de chirurgie - Groupe Hospitalier Diaconesses-Croix Saint Simon

DATA-MANAGEMENT

✓ Le **data-management** sera assuré par la DRCI du CLB :

Leila BEN ABDESSELEM – ☏ : 04.78.78.29.22 - 💻 : Leila.benabdesselem@lyon.unicancer.fr

GESTION ADMINISTRATIVE ET REGLEMENTAIRE

✓ La **gestion administrative et règlementaire** sera assurée par la Direction de la Recherche Clinique et de l'Innovation du CLB :

Pôle Affaires réglementaires :
Séverine GUILLEMAUT- ☏ : 04.78.78.29.68 - 💻 : severine.guillemaut@lyon.unicancer.fr

Pôle Contractualisations et partenariats :
Sophie DARNIS - ☏ : 04.78.78.29.19 - 💻 : sophie.darnis@lyon.unicancer.fr

VIGILANCE

✓ La gestion des données de **vigilance** sera assurée par le Centre Régional de Pharmacovigilance (CRPV) de Lyon :

Judith COTTIN- ☏ : 04 72 11 93 90 - 💻: judith.cottin@chu-lyon.fr
Anne MILLARET - ☏ : 09 83 77 53 07- 💻 : pharmacovigilance.clb@gmail.com

Sommaire

Synopsis....5

Tableau de synthèse 10

Glossaire des abréviations 12

Avant propos.... 13

I Introduction et rationnel 14

I.1 Résection chirurgicale des métastases hépatiques de cancers colorectaux : état des lieux. 14

I.2 Les ultrasons thérapeutiques 14

I.3 Développement d'une sonde HIFU torique dédiée au traitement des MHCCR 15

I.3.a Evaluation préclinique de la faisabilité des traitements HIFU : étude de la lésion unitaire et stratégie d'élargissement de la nécrose 16

I.3.b Evaluation préclinique de l'efficacité des traitements HIFU à l'aide d'un modèle de pseudotumeur.... 17

I.3.c Collaboration avec la société EDAP - Développement d'un prototype utilisable en peropératoire chez l'Homme 17

I.4 Expérience antérieure de l'équipe sur le sujet 18

I.5 Nouveaux développements technologiques 18

II Généralités concernant la présente étude 20

II.1 Indication thérapeutique.... 20

II.2 Type d'étude.... 20

II.3 Balance benéfices / risques 20

II.4 Schéma général de l'étude 21

III Objectifs et critères de jugement associés 21

III.1 Phase I.... 21

III.1.a Objectifs principaux.... 21

III.1.b Objectifs secondaires 22

III.2 Phase II.... 23

III.2.a Phase IIa 23

III.2.b Phase IIb 24

IV Méthodologie 25

IV.1 Population 25

IV.1.a Critères d'inclusion.... 25

IV.1.b Critères de non-inclusion 25

IV.1.c Critère d'inclusion spécifique à la phase IIb (hors étape préliminaire) 25

IV.2 Déroulement de l'étude.... 26

IV.2.a Préalable à la mise en place de l'étude 26

IV.2.b Inclusion 26

IV.2.c Interventions chirurgicales 26

IV.2.d Suivi postopératoire 29

IV.3 Bilans d'évaluation.... 30

IV.3.a Bilans communs à toutes les étapes 30

IV.3.b Phase I 30

IV.3.c Phase IIa 31

IV.3.d Phase IIb : étape préliminaire ... 31
IV.3.e Phase IIb : étapes 1 et 2 ... 32
IV.4 Critères de sortie d'étude ... 32
IV.5 Arrêt prématuré de l'étude ... 32
V Vigilance ... 33
V.1 Notification des évènements indésirables par l'Investigateur ... 33
V.1.a Notification des événements indésirables dans le cahier d'observation ... 33
V.1.b Notification des événements indésirables graves au Promoteur ... 33
V.1.c Notification des grossesses au Promoteur ... 35
V.2 Évènements indésirables ne nécessitant pas une déclaration immédiate ... 35
Événements non liés à l'utilisation du DM expérimental : hospitalisations programmées préalablement au début de l'étude. ... 35
V.3 Évènements indésirables imputables à la pathologie ou à la chirurgie ... 35
V.4 Effets indésirables attendus – Définition du document de référence ... 35
V.5 Archivage des déclarations d'événement indésirable ... 36
VI Méthodologie statistique ... 37
VI.1 Nombre de participants et règles statistiques ... 37
VI.1.a Phase I ... 37
VI.1.b Phase II ... 37
VI.2 Définition des populations d'analyse ... 40
VI.3 Analyses ... 40
VII Résultats attendus ... 40
VIII Gestion et coordination de l'essai ... 41
VIII.1 Pilotage ... 41
VIII.2 Recueil de données ... 41
VIII.3 Contrôle qualité des données ... 41
VIII.4 Traitement informatique ... 41
VIII.5 Analyses statistiques ... 41
VIII.6 Rédaction du rapport d'étude ... 41
VIII.7 Publications ... 42
IX Aspects éthiques et règlementaires ... 43
IX.1 Instances impliquées dans l'étude ... 43
IX.1.a Comité de Protection des Personnes ... 43
IX.1.b Autorité compétente ... 43
IX.1.c Comité Consultatif sur le Traitement de l'Information en matière de Recherche dans le domaine de la Santé – Commission Nationale de l'Informatique et des Libertés ... 43
IX.2 Assurance ... 43
IX.3 Confidentialité ... 43
IX.4 Audits et inspections ... 44
IX.4.a Audits par le Centre de Coordination ... 44
IX.4.b Inspections par les autorités de santé ... 44
X Références bibliographiques ... 45
Annexes ... 47

Synopsis

TITRE ABREGE

HIFU

TITRE

Evaluation, chez des patients nécessitant une chirurgie de résection de métastases hépatiques, de l'utilisation per-opératoire d'ultrasons focalisés de haute intensité (HIFU) : faisabilité, innocuité, et capacité de ciblage des métastases.

NUMERO DU PROJET

N° CLB : ET2009-068

N° ID RCB : 2009-A00779-48

VERSION ET DATE

N°4.0 – 15/07/2014

PROMOTEUR

Centre Léon Bérard (CLB) - LYON

INVESTIGATEUR-COORDONNATEUR

Pr Michel RIVOIRE – Département de chirurgie – CLB

CENTRE DE COORDINATION

Direction de la Recherche Clinique et de l'Innovation (DRCI)- CLB

INDICATION THERAPEUTIQUE

Traitement chirurgical des métastases hépatiques (MH).

RATIONNEL

En France, le cancer colorectal (CCR) est le second cancer en terme d'incidence avec environ 36 000 nouveaux cas chaque année (source : réseau français des registres des cancers). Le pronostic reste grave avec environ 16 000 décès par an, le plus souvent liés à la présence de métastases.

La résection hépatique constitue le traitement de référence des MHCCR et reste à ce jour le seul traitement curatif. Toutefois, seule une minorité des patients (10 à 20 %) peuvent en bénéficier. En conséquence, de nombreuses techniques visant à trouver une alternative à la résection chirurgicale des MHCCR, telles que la radiofréquence ou la cryothérapie, ont été développées et évaluées. Cependant, l'utilisation de ces techniques, très répandues, présente des limites : impossibilité de monitorage fiable en temps réel, geste invasif, faible taille des volumes hépatiques détruits, durée de traitement longue, taux de récidive locale élevé.

Il s'avère donc indispensable de développer de nouveaux procédés de destruction tumorale qui doivent être précis, sans limite en terme de taille, non invasifs pour l'organe cible et qui puissent être utilisés de manière complémentaire à la chirurgie.

Les ultrasons focalisés de haute intensité (HIFU) sont des ondes mécaniques, donc non ionisantes, qui permettent, en quelques secondes, de générer une nécrose cellulaire par coagulation. La brièveté du phénomène rend les traitements peu dépendants de la perfusion. L'application clinique peropératoire des HIFU pour le traitement des MHCCR est un procédé qui n'a pas encore été exploré. L'équipe chirurgicale du Centre Léon Bérard (CLB), en collaboration avec l'équipe INSERM U556 au sein de l'Institut de Chirurgie Expérimentale (ICE) de Lyon, a entrepris un programme de recherche sur ce sujet. Les travaux préliminaires *in vitro* et précliniques ont ainsi démontré l'intérêt, la faisabilité et la tolérance clinique et biologique du traitement par HIFU des MHCCR.

Ces résultats permettent d'envisager la mise en place d'une étude clinique, qui fait l'objet du présent protocole.

TYPE D'ETUDE

Essai clinique de phase I-II, prospectif, d'évaluation d'un dispositif médical (DM) chirurgical (1ère utilisation chez l'Homme), monocentrique.

OBJECTIFS PRINCIPAUX DE L'ETUDE

1ère partie (Phase I - sur du foie sain à réséquer)

- ✓ Faisabilité de tirs HIFU,
- ✓ Sécurité d'utilisation du DM,
- ✓ Tolérance des tirs HIFU.

2ème partie (Phase IIa - sur du foie sain à réséquer, au niveau d'un repère stable puis à une distance prédéfinie par rapport à un repère stable.)

- ✓ Capacités de ciblage des tirs HIFU par rapport à une zone à détruire, matérialisée par un repère stable, d'une part (palier 1), et à une distance prédéfinie par rapport à une zone à éviter, matérialisée par un repère stable, d'autre part (palier 2).

3ème partie (Phase IIb – Sonde de traitement à balayage électronique - Etape préliminaire : sur du foie sain à réséquer, au niveau d'un repère stable ; étapes 1 et 2 = transducteur classique ou transducteur torique à croisement intérieur : sur des métastases à réséquer)

- ✓ Capacité, en sommant les tirs HIFU, à générer des lésions englobant une métastase et garantissant une marge de sécurité suffisante dans le tissu sain.

POPULATION

Critères d'inclusion

- Homme ou femme âgé(e) de 18 ans ou plus,
- Atteint(e) de métastase(s) hépatique(s) d'un cancer colorectal,

  NB : à titre exceptionnel, l'inclusion de patients porteurs de métastase(s) hépatique(s) d'une autre origine est possible.
- Devant subir une hépatectomie par laparotomie en vue de la résection de métastase(s) hépatique(s), au Centre Léon Bérard,
- Performance status ECOG ≤ 1
- Affilié(e) à un régime de sécurité sociale,
- Ayant daté et signé un consentement éclairé.

Critère d'inclusion spécifique à la phase IIb (hors étape préliminaire)

- Patients présentant une ou plusieurs métastase(s) hépatique(s) de taille inférieure ou égale à 2 cm devant être réséquée(s) lors de l'hépatectomie objet de la présente étude, et ce que cette résection ait lieu dans le cadre d'une chirurgie en un temps ou bien dans le cadre d'une chirurgie de résection programmée en deux temps.

Critères de non inclusion

- Ayant déjà subi une chirurgie hépatique majeure (résection de plus de 3 segments) ou biliaire majeure (contexte de chirurgie hépatique itérative complexe),
- Ayant déjà subi une chirurgie abdominale majeure à l'exclusion d'une chirurgie colorectale pour le traitement de sa tumeur primitive ou exceptionnellement une chirurgie abdominale autre pour le traitement de sa tumeur primitive si celle-ci n'est pas colorectale (la chirurgie de la vésicule biliaire par laparoscopie dans un délai supérieur à 6 mois ne constitue pas un critère de non inclusion),
- Ne pouvant être suivi(e) pour des raisons sociales, familiales, géographiques ou psychologiques,

- Femme enceinte ou allaitante (un test de grossesse sérique ou urinaire doit être négatif au moment de l'inclusion dans l'étude pour les femmes en âge de procréer ; une méthode de contraception fiable devra être utilisée durant la durée de l'étude).

DEROULEMENT DE L'ÉTUDE

Inclusion

L'inclusion aura lieu lors de la consultation préalable à la chirurgie (visite préalable au premier temps chirurgical dans le cas d'une chirurgie de résection programmée en deux temps (phase IIb de l'étude seulement)).

Intervention

✓ **Préparation**

En début d'intervention chirurgicale (ou au cours du premier temps chirurgical en cas de chirurgie de résection programmée en deux temps) après libération complète du foie, confirmation de l'indication de résection hépatique et déconnection du foie à réséquer, avant réalisation des gestes spécifiques de la résection hépatique, le chirurgien investigateur réalisera le traitement HIFU sur la partie de foie qu'il est prévu de réséquer.

✓ **Phase I**

Les tirs porteront sur le foie sain à réséquer, à distance des structures vasculaires importantes et des métastases. Deux tirs HIFU seront réalisés sur chaque patient : une lésion superficielle et une lésion en profondeur.

✓ **Phase IIa**

Les tirs porteront sur le foie sain à réséquer. Après positionnement d'un repère stable, deux tirs HIFU seront réalisés sur chaque patient (2 lésions). Dans un premier temps (palier 1), les lésions devront cibler le repère stable. Dans un second temps palier 2), les lésions devront être produites à une distance prédéfinie par rapport au repère stable.

✓ **Phase IIb**

A l'étape préliminaire, les tirs porteront sur le foie sain à réséquer. Après positionnement d'un repère stable, les tirs HIFU seront réalisés sur chaque patient avec une sonde à balayage électronique et cibleront le repère stable (de 1 à 2 lésions).

Aux étapes 1 et 2 les tirs porteront sur les métastases à réséquer de petite taille (≤ 20 mm) et le foie sain péri-lésionnel. Plusieurs métastases pourront être ciblées par patient. Un transducteur classique sera utilisé pour les lésions proches des vaisseaux ou un transducteur torique à croisement intérieur pour les lésions sous capsulaires.

Quelle que soit la phase, il n'y aura aucune modification en ce qui concerne l'hépatectomie qui sera en tout point identique à celle qui aurait été réalisée en l'absence de tirs HIFU.

Suites de l'intervention

A la suite de la réalisation des tirs HIFU, une exploration de l'ensemble de la cavité abdominale sera réalisée afin de détecter d'éventuels effets collatéraux.

L'analyse anatomopathologique (qui sera réalisée à l'issue de l'intervention chirurgicale ayant conduit à la résection de(s) métastase(s) hépatique(s) et de la zone de traitement par HIFU) comportera en plus une analyse de chacune des lésions HIFU.

Suivi postopératoire

Les patients seront suivis pendant 30 jours à l'issue de l'intervention chirurgicale ayant conduit à la résection de(s) métastase(s) hépatique(s) et de la zone de traitement par HIFU (même temps opératoire si chirurgie en un temps ou seconde intervention si chirurgie programmée en deux temps pour les patients de la phase IIb).

SCHEMA DE L'ETUDE & NOMBRE DE PARTICIPANTS

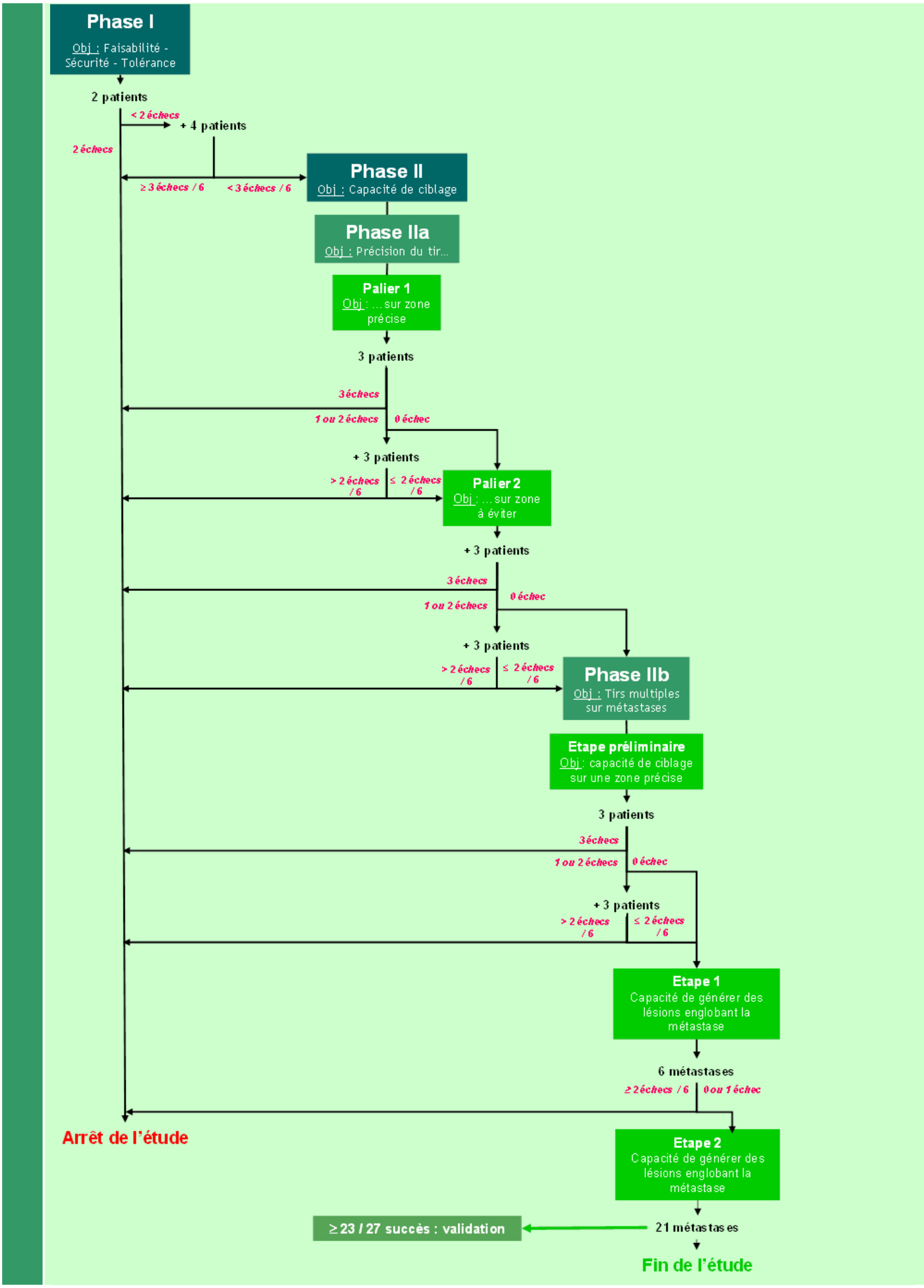

METHODES STATISTIQUES

Cette étude prévoit d'inclure 35 à 44 patients (phase I : 6 patients, phase IIa : 6 à 12 patients, phase IIb : 23 à 26 patients).

La phase I sera réalisée en 2 étapes selon une règle séquentielle (critères de Lee) afin d'arrêter précocement l'étude si un taux d'échec > 15 % est observé. Le passage à la phase II implique que moins de 3 échecs soient constatés à l'issue de l'analyse des 6 patients inclus.

Dans la phase IIa, un effectif de 6 à 12 patients sera inclus en 2 paliers successifs comprenant chacun 3 à 6 patients. Il s'agit d'être capable de mettre en évidence une insuffisance de précision des tirs HIFU si cet événement survient avec une fréquence élevée. En utilisant la loi binomiale, des effectifs de respectivement 3 et 6 patients permettent ainsi de garantir une probabilité de 90 % d'observer au moins un patient avec une précision de tir inacceptable, si cet événement se produit respectivement dans au moins 54 % et 32 % des cas.

La phase IIb comprendra 3 étapes :

- une étape préliminaire inclura de 3 à 6 patients. Comme précédemment (palier 1 de la phase IIa), il s'agit d'être capable de mettre en évidence une insuffisance de précision des tirs HIFU, mais en utilisant une sonde à balayage électronique.
- l'effectif des étapes suivantes (étapes 1 et 2 utilisant un transducteur classique ou un transducteur torique à croisement intérieur) a été calculé en utilisant un plan expérimental de Simon en 2 étapes (Optimal design) (p_0 = 0,70 - p_1 = 0,90). Avec un risque d'erreur alpha égal à 5 % et une puissance de 80 %, 27 métastases devront être ciblées pour rejeter H_0: $P \leq p_0$ *versus* H_1: $P \geq p_1$ en situation unilatérale. En considérant qu'en moyenne 1,5 métastases seront traitées par patient inclus, un effectif de 20 patients est nécessaire. L'observation d'un nombre de succès (réalisation d'une lésion HIFU conforme aux critères de jugement principal) supérieur ou égal à 23 permettra de conclure au rejet de H_0 pour accepter H_1.

Pour chacune des phases de l'étude, les analyses seront réalisées sur la population évaluable pour la faisabilité clinique, la tolérance et l'efficacité du traitement des MH par HIFU. Cette population comprendra tous les patients inclus dans l'étude et pour lesquels une hépatectomie aura pu être réalisée.

Les analyses seront réalisées sur une base descriptive. Les variables qualitatives seront décrites par leurs distributions de fréquences et les pourcentages correspondants avec leur intervalle de confiance à 95 %. Les variables quantitatives seront décrites par leur nombre d'occurrence, moyenne, écart-type, médiane, valeurs minimum et maximum.

Les résultats des analyses intermédiaires obtenus à l'issue de chacune des phases de l'étude seront communiqués au Comité de Pilotage de l'étude. Ce Comité se réunira pour prendre les décisions nécessaires quant à la poursuite de l'étude au fur et à mesure de son déroulement, en accord avec le Promoteur.

CALENDRIER

**Début de l'étude :**	mars 2010
**Durée d'inclusion :**	66 mois (septembre 2015)
**Durée de suivi :**	30 jours post opératoire
**Durée de l'essai :**	67 mois (octobre 2015)

Tableau de synthèse

Phase	Objectif principal	Nombre de patients / de lésions	Règles statistiques
**I**	Faisabilité - Sécurité d'utilisation - Tolérance	6 patients<br>2 lésions / patient<br>Soit 12 lésions à différentes profondeurs	1ere analyse après 2 patients :<br>- si < 2 échecs sont constatés ➨ poursuite de l'étude<br>- si 2 échecs ➨ arrêt de l'étude.<br>2ème analyse après 6 patients :<br>- si < 3 échecs sont constatés ➨ poursuite de l'étude<br>- si ≥ 3 échecs ➨ arrêt de l'étude.
**IIa Palier 1**	Capacité de ciblage des tirs sur une zone précise	3 à 6 patients<br>2 lésions / patient<br>Soit 6 à 12 lésions centrées sur repère	- Si 0 échec ➨ passage au 2eme palier ;<br>- Si 1 ou 2 échec(s) ➨ inclusion de 3 patients supplémentaires au 1er palier<br>↳Si > 2 échecs sur les 6 patients ➨ arrêt de l'étude,<br>↳Si ≤ 2 échecs sur les 6 patients ➨ passage au 2ème palier ;<br>- Si 3 échecs ➨ arrêt de l'étude.
**Palier 2**	Capacité de ciblage des tirs à distance d'une zone à épargner	3 à 6 patients<br>2 lésions / patient<br>Soit 6 à 12 lésions à une distance prédéfinie du repère	- Si 0 échec ➨ passage à la phase IIb ;<br>- Si 1 ou 2 échec(s) ➨ inclusion de 3 patients supplémentaires au 2ème palier<br>↳Si > 2 échecs sur les 6 patients ➨ arrêt de l'étude,<br>↳Si ≤ 2 échecs sur les 6 patients ➨ passage à la phase IIb ;<br>- Si 3 échecs ➨ arrêt de l'étude.
**IIb**<br>**Etape préliminaire**	Capacité de ciblage des tirs sur une zone précise	3 à 6 patients<br>1 à 2 lésions / patient<br>Soit 3 à 12 lésions centrées sur repère	- Si 0 échec ➨ passage à l'étape 1<br>- Si 1 ou 2 échec(s) ➨ inclusion de 3 patients supplémentaires à l'étape préliminaire<br>↳Si > 2 échecs sur les 6 patients ➨ arrêt de l'étude,<br>↳Si ≤ 2 échecs sur les 6 patients ➨ passage à l'étape 1<br>- Si 3 échecs ➨ arrêt de l'étude.

**IIb**<br>**Etape 1**	Faisabilité de somation des tirs sur des métastases à réséquer : capacité à générer une lésion englobant la métastase avec des marges de sécurité suffisante :	6 métastases ciblées<br>En considérant en moyenne 1,5 métastases traitées / patient, l'inclusion de 4 à 5 patients est attendue	- Si 0 ou 1 échec sur les 6 métastases ➡ passage à l'étape 2<br>- Si ≥ 2 échecs sur les 6 métastases ➡ arrêt de l'étude
**IIb**<br>**Etape 2**	soit transducteur classique utilisé pour les lésions proches des vaisseaux ; soit transducteur torique à croisement intérieur utilisé pour les lésions sous-capsulaires	21 métastases ciblées<br>En considérant en moyenne 1,5 métastases traitées / patient, l'inclusion d'une quinzaine de patients est attendue	Analyse sur les 27 métastases ciblées (étapes 1 et 2 de la phase IIb)<br>- Si ≥ 23/27 succès ➡ validation de la phase IIb

Glossaire des abréviations

- AEC — Assistant d'Essai Clinique
- AIVOC — Anesthésie intraveineuse à objectif de concentration
- ANSM — Agence Nationale de Sécurité du Médicament et des produits de santé
- ARC — Attaché de Recherche Clinique
- CCTIRS — Comité Consultatif pour le Traitement de l'Information en matière de Recherche
- CLB — Centre Léon Bérard
- CNIL — Commission Nationale Informatique et Liberté
- CPP — Comité de Protection des Personnes
- CRF — Case Report Form
- CRPV — Centre Régional de Pharmacovigilance
- DC — Débit cardiaque
- DM — Dispositif médical
- DRCI — Direction de la Recherche Clinique et de l'Innovation
- EI — Evènement Indésirable
- EIG — Evènement Indésirable Grave
- FC — Fréquence cardiaque
- FR — Fréquence respiratoire
- HIFU — High Intensity Focused Ultrasound / ultrasons focalisés de haute intensité
- IBODE — Infirmier de Bloc Opératoire Diplômé d'Etat
- IC — Index cardiaque
- ICE — Institut de Chirurgie Expérimentale
- INSERM — Institut Nationale de la Santé et de la Recherche Médicale
- MH — Métastases Hépatiques
- MHCCR — Métastases Hépatiques de Cancers Colorectaux
- OMS — Organisation Mondiale de la Santé
- PA — Pression artérielle
- PAO_2 — Pression artérielle en O_2
- $PetCO_2$ — Pression télé expiratoire en CO_2
- RVP — Résistance vasculaire périphérique
- SpO_2/CO_2 — Saturation périphérique en O_2/CO_2
- SvO_2/CO_2 — Saturation du sang veineux mêlé en O_2/CO_2
- TRC — Technicien de Recherche Clinique
- VT — Volume courant

Avant propos

Dans le cadre de ce protocole, les termes :

- « **tir** » signifie le fait de générer une décharge d'ultrasons au moyen de la sonde à ultrasons focalisés de haute intensité objet de la présente évaluation, dans le but de générer une zone de nécrose tissulaire,
- « **lésion** » signifie la zone de nécrose générée par l'utilisation de la sonde à ultrasons focalisés de haute intensité objet de la présente évaluation.

I Introduction et rationnel

I.1 Résection chirurgicale des métastases hépatiques de cancers colorectaux : état des lieux

Les métastases hépatiques de cancers colorectaux (MHCCR) représentent un problème majeur de santé publique (16 000 décès par an en France) (1). Malgré les progrès récents de la chimiothérapie (2) et des traitements par agents physiques (radiofréquence et cryothérapie) (3, 4), la résection chirurgicale reste le seul traitement permettant d'obtenir une survie à long terme, voire une guérison définitive dans un certain nombre de cas. La survie à 5 ans après chirurgie de résection est comprise entre 20 et 35 %. Cependant, seule une minorité des patients (10 à 20 %) peuvent bénéficier d'une chirurgie de résection. Le plus souvent, le nombre, la localisation, la taille des métastases hépatiques, ou la faiblesse du volume hépatique fonctionnel restant contre-indiquent le geste chirurgical (5, 6).

En conséquence, de nombreuses techniques ont été évaluées pour trouver une alternative à la résection chirurgicale des MHCCR. Les principales techniques actuelles de destruction focalisée des MHCCR sont la radiofréquence, la cryothérapie, les micro-ondes et l'hyperthermie laser (7). La radiofréquence et la cryothérapie, largement utilisées, ont permis d'augmenter le nombre de patients traités avec des résultats encourageants. Ces techniques souffrent cependant de l'absence de moyen de monitorage fiable en temps réel (les évaluations par scanner et IRM ne permettent pas d'ajuster le traitement au cours de l'intervention) et nécessitent un geste invasif (sonde introduite dans la métastase à traiter). Une limite supplémentaire est la faible taille des volumes hépatiques ciblés. Enfin, les taux de récidives locales à 2 ans sont élevés, entre 30 % et 40 % pour la cryothérapie et de l'ordre de 15 % (2 % - 60 %) pour la radiofréquence (3, 8).

Il s'avère donc indispensable de développer de nouveaux procédés de destruction tumorale qui doivent être précis, sans limite en terme de taille, non traumatisants pour le foie et complémentaire de la chirurgie. Le but est de permettre d'augmenter le nombre de patients pouvant bénéficier d'une chirurgie d'exérèse à visée curative et de diminuer le risque de récidive après résection hépatique. Dans ce cadre, les méthodes de destruction focalisée des MHCCR permettent une bonne efficacité thérapeutique tout en préservant au maximum le foie sain.

I.2 Les ultrasons thérapeutiques

Les ultrasons focalisés de haute intensité (HIFU) sont des ondes mécaniques générées par un transducteur ultrasonore. L'effet de focalisation permet une absorption brutale d'énergie confinée dans la zone focale située à distance de l'émetteur et dont les dimensions sont parfaitement maîtrisées. L'absorption d'énergie ultrasonore crée une élévation de température de 40 à 60°C en quelques secondes, localisée dans la zone focale, entraînant une nécrose cellulaire par coagulation, de forme ellipsoïdale dont les dimensions sont typiquement un grand axe de 15 mm et un diamètre de 20 mm. La brièveté du phénomène rend les traitements peu dépendants de la perfusion. L'avantage est de bénéficier d'une onde non ionisante qui peut être fortement focalisée, donc très peu dangereuse pour les tissus situés en amont ou en aval de la zone traitée et qui présente de plus des effets secondaires quasi nuls. Ainsi, depuis une vingtaine d'années, les applications thérapeutiques des ultrasons (hyperthermie, HIFU, lithotritie) ne cessent de se multiplier et explorent de nombreuses voies : extracorporelle, percutanée, transrectale, laparoscopique, transœsophagiène, per-opératoire. Les études précliniques et cliniques concernent des traitements très variés telles ceux des glaucomes (9, 10), des cancers localisés de la prostate (11, 12), de l'hypertrophie bénigne de la prostate (13, 14), de la vessie (15), du sein (16), de l'œsophage (17), du foie (18), du fibrome utérin (19) ou encore du cerveau (20). Selon l'accessibilité des organes à traiter et des voies de traitements adoptées, différentes technologies de transducteurs voient le jour. Les ultrasons thérapeutiques apparaissent aujourd'hui en bonne place au sein des traitements émergents par agents physiques complémentaires à la chirurgie. Malgré ces avantages, des innovations sont nécessaires pour rendre les HIFU adaptés au traitement des MHCCR. Il est en particulier nécessaire de diminuer le temps d'intervention car les lésions HIFU élémentaires sont ellipsoïdales et de petites dimensions. Ainsi un traitement par HIFU consiste à juxtaposer de manière précise (généralement par un déplacement robotisé de la sonde de traitement) ces lésions élémentaires jusqu'à englober la tumeur ciblée avec une marge de tissus sains. Actuellement un traitement HIFU des cancers de prostate nécessite en moyenne 149 minutes (21). De plus, un

système d'imagerie en temps réel doit être couplé aux HIFU pour visualiser avec précision la zone traitée après chaque exposition ultrasonore.

Des traitements des cancers primitifs du foie par HIFU sont actuellement testés par des équipes chinoises et anglaises avec une approche purement extracorporelle mais l'application soulève des difficultés qui ne sont pas encore résolues : propagation ultrasonore gênée par les os, les espaces contenant de l'air et difficulté de ciblage en raison de la respiration. De plus, il est difficile d'atteindre les régions profondes du foie.

Pour toutes ces raisons, l'objectif des travaux réalisés par l'équipe de chirurgie du CLB en collaboration avec l'équipe de l'INSERM U556 au sein de l'ICE de Lyon a été de développer au stade préclinique, un prototype de sonde HIFU adapté au traitement des MHCCR, capable de générer des lésions volumineuses dans le foie.

I.3 Développement d'une sonde HIFU torique dédiée au traitement des MHCCR

Cf. ref biblio (22, 23)

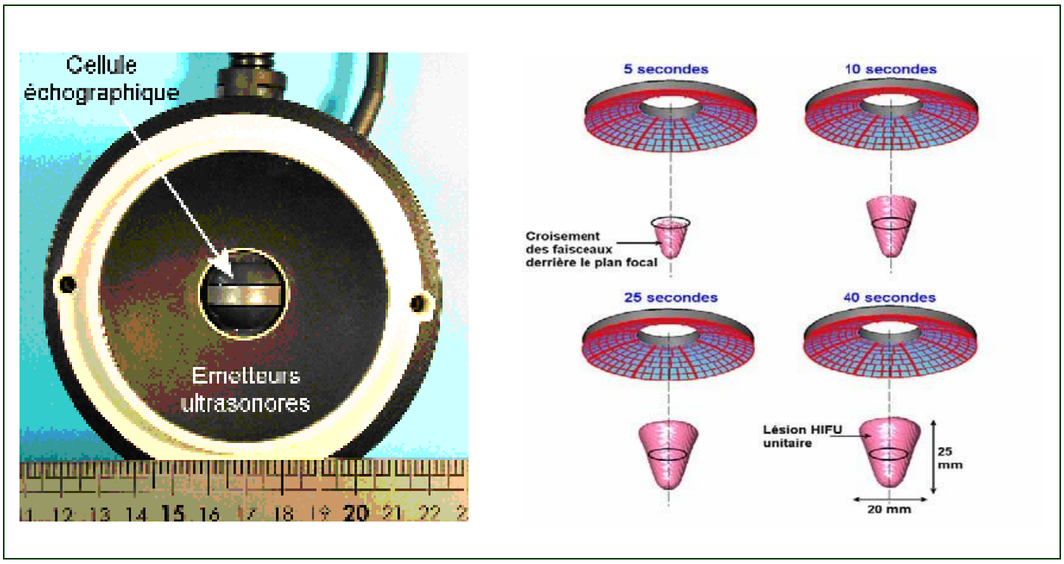

Figure 1 : Sonde HIFU.

Une sonde médicale peropératoire de thérapie ultrasonore dédiée à l'ablation par HIFU des MHCCR a été développée et validée au stade préclinique sur le modèle porcin. Des ablations coniques unitaires de 7 cm^3 (diamètre de 20 mm et profondeur de 25 mm) ont pu être générées en 40 secondes par l'intermédiaire d'un transducteur de forme torique croisée ce qui représente une stratégie efficace en termes de temps et de volume de traitement HIFU dans le foie. Une ouverture au centre du dispositif médical (DM) de thérapie permet de loger une sonde échographique pour le repérage des métastases hépatiques et le contrôle du traitement par échographie. Les caractéristiques de ces lésions ont été étudiées en modélisation et lors d'essais précliniques *in vitro* et *in vivo*.

I.3.a Evaluation préclinique de la faisabilité des traitements HIFU : étude de la lésion unitaire et stratégie d'élargissement de la nécrose

Cf. ref biblio (24, 25)

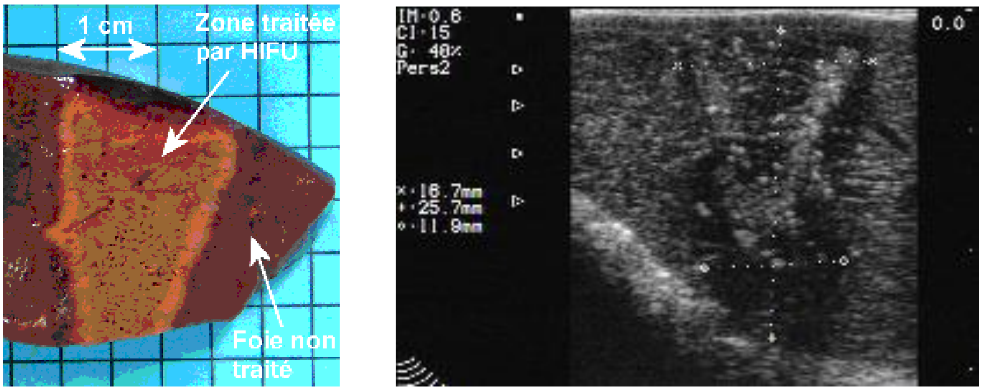

(a) **(b)**

<u>Figure 2 :</u> (a) Lésion HIFU élémentaire produite en 40 secondes.

(b) Lésion HIFU élémentaire observée par échographie durant le traitement.

Un point majeur pour le traitement des MHCCR est la qualité du ciblage car en chirurgie classique, comme avec les traitements par agents physiques (radiofréquence, cryothérapie), un traitement est considéré comme efficace lorsque la tumeur est détruite avec des marges géométriques négatives. La lésion élémentaire réalisée avec la sonde HIFU torique dédiée au traitement des MHCCR a été étudiée en détail au stade préclinique dans le foie de porc *in vivo*. Ses caractéristiques géométriques (Figure 2a) et son indépendance vis-à-vis de la perfusion se sont révélées adaptées au traitement des métastases dans un organe volumineux et très perfusé tel que le foie. L'utilisation manuelle du prototype de sonde HIFU torique sous une approche peropératoire a permis de générer des traitements localisés dans le foie, avec précision, grâce à la qualité du guidage par échographie intégrée à la sonde de traitement. Le traitement par HIFU torique proposé semble donc convenir puisque le positionnement et l'établissement de la nécrose dans les tissus hépatiques sont bien maîtrisés. Les lésions sont homogènes et ont des frontières bien définies dans le foie aux niveaux macroscopique et microscopique.

Les lésions produites apparaissent distinctement sur les images échographiques (Figure 2b) ce qui permet de juxtaposer plusieurs lésions élémentaires coniques afin augmenter le volume de nécrose (Figure 3). Le traitement est non invasif pour l'organe et très bien toléré.

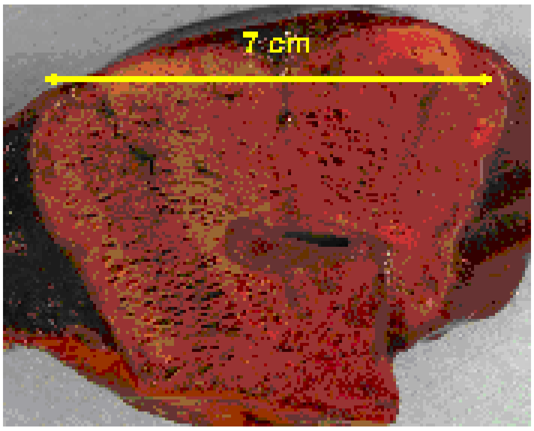

<u>Figure 3 :</u> Juxtaposition de 6 lésions élémentaires

I.3.b Evaluation préclinique de l'efficacité des traitements HIFU à l'aide d'un modèle de pseudotumeur

Cf. ref biblio (26)

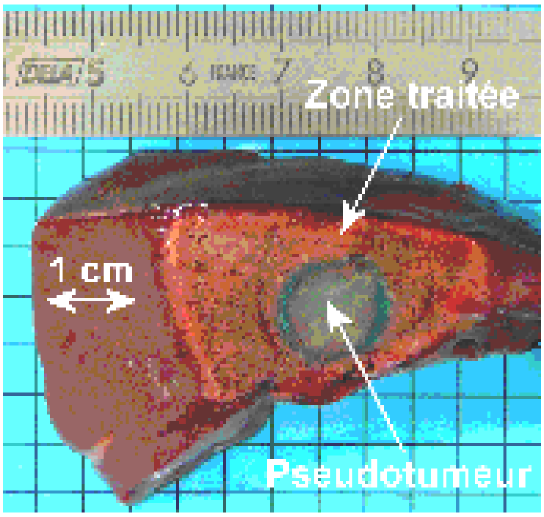

Figure 4 : PseudoTumeur HIFU

Ces études avaient pour objectif l'évaluation préclinique des performances de ciblage du prototype de sonde HIFU torique dédié au traitement des MHCCR, en utilisant un modèle pseudotumoral implanté dans le foie de porc. Ce modèle est repérable en échographie et en anatomopathologie et permet d'évaluer la qualité du ciblage de la thérapie HIFU (Figure 4). Des lésions ont donc été réalisées en assurant des marges négatives autour des pseudotumeurs dans toutes les directions.

Les études de tirs HIFU sur le modèle de pseudotumeur ont permis de quantifier la précision du traitement HIFU et l'efficacité de la juxtaposition des lésions élémentaires pour réaliser des lésions volumineuses et bien ciblées dans le foie.

A travers ces différentes études précliniques, l'intérêt de l'approche peropératoire et l'efficacité de l'utilisation manuelle de la sonde HIFU torique ont été mis en évidence.

I.3.c Collaboration avec la société EDAP - Développement d'un prototype utilisable en peropératoire chez l'Homme

Les études précédemment décrites ont été menées avec un prototype expérimental développé par l'unité INSERM 556 en collaboration avec la société EDAP. Cette société est leader mondial dans les HIFU pour le traitement du cancer de la prostate. Dans cette indication, elle a développé l'Ablatherm® qui est commercialisé et utilisé dans le monde entier.

La collaboration mise en place et les matériels utilisés dans le cadre des études précédemment citées ont été financés dans le cadre d'un programme « Preuve de Concept » du Cancéropôle Lyon Auvergne Rhône Alpes (CLARA). Les résultats très positifs obtenus ont conduit la société EDAP, avec l'aide d'Oséo-ANVAR, à développer un nouveau DM utilisable en clinique humaine : le prototype MFOCUS®.

I.4 Expérience antérieure de l'équipe sur le sujet

L'équipe de chirurgie du CLB possède une expertise reconnue dans la prise en charge des métastases hépatiques qui se traduit par une activité clinique importante (80 à 100 interventions/an), par le développement de techniques de destruction focalisée alternatives ou complémentaires à la chirurgie, par la participation à de nombreuses études cliniques et par une collaboration régulière avec les équipes d'oncologie médicale (4).

Depuis maintenant 3 ans, en collaboration avec l'équipe INSERM U556, a été développé un programme de recherche sur l'utilisation des HIFU pour le traitement des MHCCR (23). L'expérience accumulée au travers d'applications *in vitro* puis précliniques chez l'animal, relatée dans des publications nationales et internationales, permet aujourd'hui d'envisager l'évaluation de la faisabilité, de la tolérance et de l'efficacité de cette technique chez l'Homme, dans le cadre d'études cliniques rigoureusement menées.

Ces résultats et les collaborations mises en place permettent d'envisager la réalisation d'une évaluation clinique des performances de ce nouveau procédé thérapeutique de traitement des métastases hépatique, objet du présent protocole.

Il s'agira d'une première utilisation chez l'Homme, et d'une première évaluation mondiale d'une technique d'HIFU peropératoire pour la destruction focalisée des métastases hépatiques, complémentaire de la chirurgie.

I.5 Nouveaux développements technologiques

Deux nouvelles solutions technologiques ont été développées pour améliorer les stratégies thérapeutiques misent en évidence lors des travaux conduits avec le dispositif MFOCUS.

La première innovation consiste à utiliser les capacités de balayage électronique de la sonde par l'intermédiaire des 256 émetteurs qui composent la surface émettrice d'HIFU. En utilisant des lois de phases adaptées, il est possible de traiter des métastases de 2 cm de diamètre comme celles prévues dans la Phase IIb du protocole sans aucune nécessité de juxtaposition manuelle. La séquence de traitement dure 130 secondes et permet de traiter une zone de 40 mm de diamètre par 62 mm de profondeur (Figure 5). Le fait de s'affranchir d'une juxtaposition manuelle des lésions HIFU représente une amélioration significative de la sécurité d'utilisation du dispositif, ainsi que de la précision du traitement.

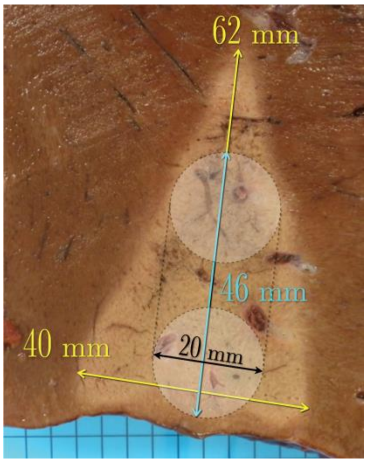

Figure 5 : Lésion HIFU de 40 mm de diamètre par 62 mm de profondeur produite en 130 secondes, sans déplacement manuel de la sonde.

Une deuxième approche a été identifiée sous la forme d'un nouveau transducteur torique. Cette nouvelle géométrie permet de croiser les faisceaux HIFU entre l'émetteur et la zone focale principale ce qui conduit à une zone de traitement élargie par rapport à la sonde précédente. Ce transducteur est découpé en 32 anneaux. En utilisant également le principe de balayage électronique nous avons montré qu'il est possible de traiter des zones de 47 mm de diamètre par 38 mm de profondeur sans juxtaposition manuelle (Figure 6). Ces nouvelles possibilités thérapeutiques vont être utilisées lors de la Phase IIb du protocole.

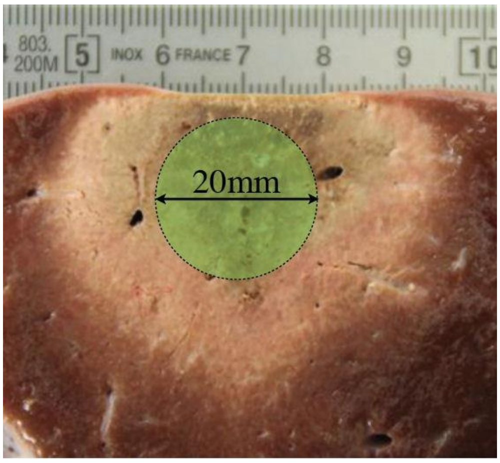

Figure 6 : Lésion HIFU de 47 mm de diamètre par 38 mm de profondeur produite en 6 minutes et 10 secondes, sans déplacement manuel de la sonde.

II Généralités concernant la présente étude

II.1 Indication thérapeutique

Traitement des métastases hépatiques des cancers colorectaux (MHCCR).

II.2 Type d'étude

Il s'agit d'une étude clinique de phase I-II, prospective, évaluant un DM chirurgical ne disposant pas du marquage CE, monocentrique (Centre Léon Bérard).

II.3 Balance bénéfices / risques

L'objectif principal de la présente étude est de confirmer sur le foie humain les résultats précliniques obtenus sur le modèle de porc. Il est à rappeler que les expérimentations réalisées sur le porc ont été bien tolérées.

Les participants à la présente étude ne pourront escompter aucun bénéfice personnel de leur participation.

Toutefois, afin d'éviter toute prise de risque, il a été décidé de le tester chez des patients opérés à visée curative pour des MH, soit dans le cadre d'une chirurgie en un temps, soit dans le cadre d'une chirurgie de résection programmée en deux temps. Ces patients candidats à une résection hépatique auront une intervention en tout point conforme à celle qui aurait été réalisée en dehors du protocole. La seule différence sera l'application de tirs HIFU sur le foie sain, le foie péritumoral ou directement sur les MH (en fonction de la phase de l'étude). Ces tirs HIFU ne seront réalisés, sur le foie destiné à être réséqué, qu'après les ultimes vérifications permettant de confirmer l'indication de résection hépatique. Cette résection aura lieu soit immédiatement après la réalisation des tirs HIFU (chirurgie de résection en un temps) soit en différé (chirurgie de résection programmée en 2 temps). En cas de chirurgie de résection programmée en deux temps, le second temps opératoire sera réalisé au plus tôt dans les 4 semaines suivant la première intervention, et au plus tard dans les 24 semaines suivant cette intervention, avec un délai médian attendu de l'ordre de 6 semaines.

La durée de la procédure de tir HIFU ne devra pas prolonger l'intervention chirurgicale de plus de 30 minutes (critère de jugement de la 1ère phase de cette étude).

Le principal risque auquel pourraient être exposés les participants à la présente étude serait une lésion des tissus de voisinage, secondaire au tir HIFU. Ce risque sera bien entendu évalué après chaque tir et ceci pendant toute la durée de l'essai. Toutes les précautions seront par ailleurs mises en œuvre pour qu'aucun accident de ce type ne survienne : des compresses de protection seront préalablement positionnées sous la zone à traiter par HIFU afin de limiter tout risque de diffusion des ondes ultrasonores, les tirs seront toujours effectués avec un axe de tir évitant les organes nobles intra abdominaux.

Dans le cadre de cette recherche, les patients seront suivis sur une durée de 30 jours postopératoires à l'issue de l'intervention chirurgicale ayant conduit à la résection de(s) métastase(s) hépatique(s) et de la zone de traitement par HIFU. Cette durée parait suffisante pour évaluer d'éventuels événements indésirables qui surviendraient tardivement. Dans le cas où un évènement de ce type venait à survenir, les patients seront pris en charge jusqu'à résolution complète de l'évènement (ou stabilisation).

Par ailleurs, l'inclusion des patients est prévue par paliers de 2, 3 ou 4 individus (selon la phase de l'étude). La décision de passer ou non à l'étape suivante sera prise par un Comité de pilotage indépendant, au regard de l'intégralité des résultats obtenus. Il est entendu que l'étude pourra également être interrompue plus précocement si un incident grave devait survenir.

De manière générale, si les résultats de cette étude s'avèrent cliniquement intéressants, le développement de cette technique représenterait une avancée majeure dans la prise en charge des patients atteints de MHCCR en permettant la mise en œuvre de chirurgies non invasives pour l'organe cible, l'extension des possibilités de traitement à des patients qui jusqu'ici ne pouvaient bénéficier d'une hépatectomie (trop gros volumes hépatiques à réséquer, localisation des métastases, etc.) ainsi que le développement de nouveaux procédés de traitement extracorporels, etc.

II.4 Schéma général de l’étude

Objectifs et critères de jugement associés

Les objectifs de l’étude se déclinent suivant les 2 phases suivantes :

II.5 Phase I

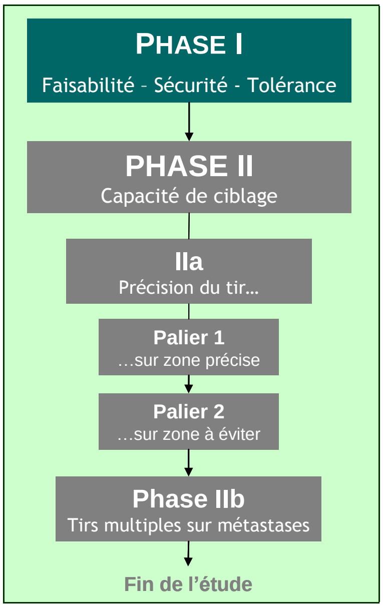

II.5.a Objectifs principaux

Il s’agit d’évaluer :

- la **faisabilité** des tirs HIFU,

  ➯ *Objectivée par :*

  1) la possibilité de réaliser des tirs :
     - à la surface du foie : dans ce cas la lésion débutera au contact de la capsule de Glisson et s’étendra dans le parenchyme hépatique sous-jacent,

     et

     - en profondeur : dans ce cas la lésion sera placée à une profondeur supérieure d’au moins 10 mm par rapport aux lésions superficielles précédemment citées.

  2) la durée supplémentaire d’intervention nécessaire à la réalisation de 2 tirs, en minutes. Elle comprend les durées suivantes :
     a. mise en service de l’appareil dans la salle opératoire ;
     b. préparation de la sonde avec le liquide de couplage ;
     c. repérage du point de tir (x2) ;
     d. réalisation du tir (x2).

     Elle ne devra pas impacter la durée de l’intervention chirurgicale de plus de 30 minutes.

     NB : Les étapes a. et b. ci-dessus pourront être réalisées en parallèle de l’intervention. Dans la mesure où elles n’allongent pas la durée d’intervention, elles ne seront pas comptabilisées. Par ailleurs, les durées de réalisation des « gestes annexes » liés à la recherche (ex : mise en place des repères) ne seront pas comptabilisées.

- la **sécurité d’utilisation** du DM,

  ➯ *Objectivée par :*

  1) la capacité à utiliser le DM sans faute d’asepsie,
  2) l’absence de lésion sur les tissus de voisinage (capsule de Glisson opposée à la zone d’entrée du tir HIFU, tissus rétro péritonéaux, rétro hépatiques et diaphragme).

     NB : les éventuelles lésions sur la capsule de Glisson au point d’entrée des ultrasons seront enregistrées mais non considérées comme un critère d’échec.

- la **tolérance** des tirs HIFU,
  - ↳ *Objectivée par :* le maintien des constantes hémodynamiques[1], respiratoires[2] et de la température corporelle du patient, pendant la phase de tir et les 5 minutes qui suivent (apprécié par l'anesthésiste).

II.5.b Objectifs secondaires

Il s'agira d'évaluer :

- l'**ergonomie de la sonde** et la faisabilité du **repérage de la zone à traiter**,
  - ↳ *Objectivées par :* la possibilité de positionnement de la sonde en fonction des différents segments du foie et de la conformation des patients. L'objectif est de tester que la lésion HIFU pourrait être placée dans un volume correspondant à au moins 80 % du foie total.

- la faisabilité du **positionnement d'un repère** (repère destiné à évaluer la capacité de ciblage lors de la phase IIa-palier 1),
  - ↳ *Objectivée par :* la possibilité de mise en place d'un repère stable, à une profondeur donnée, détectable à l'échographie,

    NB : les spécifications du DM utilisé comme repère sont décrites en annexe 1 p.48.

- la faisabilité du **repérage d'un vaisseau** (repère destiné à évaluer la capacité de ciblage lors de la phase IIa-palier 2),
  - ↳ *Objectivée par :* la possibilité de repérage d'un vaisseau au moyen d'un repère stable, détectable à l'échographie.

- la **durée de réalisation de chacune des étapes** de mise en œuvre du DM (mise en place, préparation, repérage, tir – cf. supra),
  - ↳ *Objectivée par :* les durées moyennes de réalisation de chacune des étapes, en minutes.

- la faisabilité du **monitorage immédiat de l'effet au moyen de l'échographe couplé au DM**,
  - ↳ *Objectivée par :* la possibilité de retrouver la forme de la lésion HIFU, d'identifier ses contours et d'en faire une description (diamètres, profondeur, volume, etc.) lors de l'échographie peropératoire.

- la faisabilité de la **description précoce de la lésion en anatomopathologie**,
  - ↳ *Objectivée par :* la possibilité de retrouver la forme de la lésion HIFU, d'identifier ses contours et d'en faire une description (diamètres, profondeur, volume, etc. – en mm) lors de l'examen anatomopathologique.

    NB : les procédures opératoires d'anatomopathologie seront notamment standardisées lors de cette phase et seront insérées en annexe 2 p.50.

- la faisabilité de la **mesure de la distance comprise entre le repère / le vaisseau repéré et le bord de la lésion** à l'examen anatomopathologique,
  - ↳ *Objectivée par :* l'obtention d'une mesure précise par l'anatomopathologiste, quantifiée en mm.

---

[1] Paramètres cardiaques investigués : fréquence cardiaque (FC), pression artérielle (PA), Index cardiaque (IC)

[2] Paramètres respiratoires investigués : saturation périphérique en O_2 (SpO_2) *ou* pression artérielle en O_2 (PaO_2 - prélèvements effectués avant les tirs et en fin de période d'évaluation, soit 5 min après le dernier tir, sur cathéter artériel), pression artérielle en CO_2 ($PaCO_2$ – prélèvements idem PaO_2).

II.6 Phase II

II.6.a Phase IIa

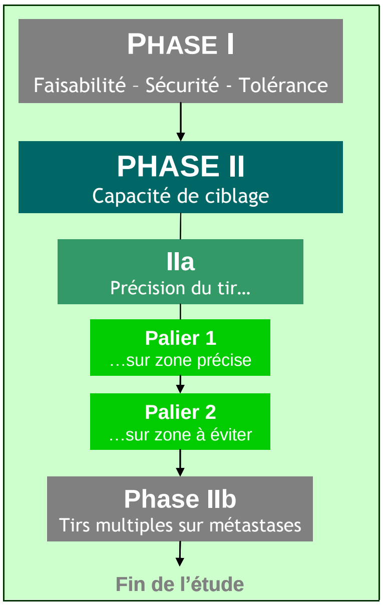

✓ **Objectif principal**

Il s'agira d'évaluer :

- la **capacité de ciblage** des tirs HIFU sur une zone précise (palier 1) puis sur zone à épargner (palier 2),

  ➯ *Objectivée par :* la distance entre l'épicentre de la lésion générée par HIFU et un repère de référence préalablement positionné d'une part (palier 1) et la distance entre le bord de la lésion et le bord d'un repère matérialisant un vaisseau (palier 2). Ces dimensions seront mesurées à l'examen anatomopathologique, en mm.

✓ **Objectifs secondaires**

Il s'agira d'évaluer :

- la **sécurité d'utilisation** du DM,

  ➯ *Objectivée par :* l'absence de lésion sur les tissus de voisinage (capsule de Glisson opposée à la zone d'entrée du tir HIFU, tissus rétro péritonéaux rétro hépatiques et diaphragme).

  NB : les éventuelles lésions sur la capsule de Glisson au point d'entrée des ultrasons seront enregistrées mais non considérées comme un critère d'échec.

- la **tolérance** des tirs HIFU,

  ➯ *Objectivée par :* le maintien des constantes hémodynamiques, respiratoires et de la température corporelle du patient (cf. § II.5.a p. 21), pendant la phase de tir et les 5 minutes qui suivent (apprécié par l'anesthésiste).

- la **corrélation entre les mesures échographiques peropératoires et macroscopiques postopératoires** de la lésion,

  ➯ *Objectivée par :* confrontation des dimensions de la lésion (diamètres, profondeur, volume, etc. – en mm), mesurées en insu, à l'échographie d'une part et immédiatement après la résection hépatique en anatomopathologie d'autre part.

II.6.b Phase IIb

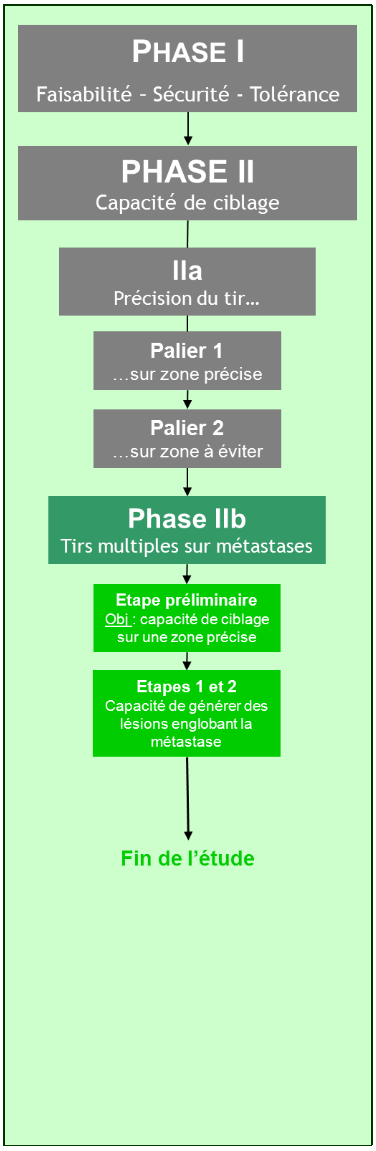

✓ **Objectif principal**

Il s'agira d'évaluer :

- Dans une étape préliminaire, **la capacité de ciblage des tirs HIFU réalisés sur une zone précise avec une sonde de traitement à balayage électronique,** cette innovation permettant d'éviter le déplacement manuel de la sonde.

  ➯ *Objectivée par :* la distance entre l'épicentre de la lésion générée par HIFU et un repère de référence préalablement positionné d'une part (de la même façon qu'au palier 1 de la phase IIa). Cette dimension sera mesurée à l'examen anatomopathologique, en mm.

  En complément, sera également évaluée dans cette étape préliminaire la capacité à générer des lésions HIFU permettant l'inclusion théorique d'une métastase de 2 cm de diamètre, avec des marges de sécurité de 3 mm. Ce critère complémentaire ne sera pas pris en compte pour le passage aux étapes suivantes de la phase IIb.

- Puis (étapes 1 et 2) la capacité de générer des lésions englobant la métastase et garantissant des marges de sécurité suffisantes dans le foie sain.

  ➯ *Objectivée par :*

  la lésion HIFU générée devra, avec un nombre de tirs inférieur ou égal à 15, englober la métastase et garantir à **l'analyse anatomopathologique :**

  macroscopiquement, une marge de sécurité supérieure ou égale à 3 mm avec une incertitude de mesure de +/- 1 mm par mesure au réglet,

  et microscopiquement, des marges non nulles dans les plans non évalués macroscopiquement.

✓ **Objectifs secondaires**

Il s'agira d'évaluer :

- la **sécurité d'utilisation** du DM,

  ➯ *Objectivée par :* l'absence de lésion sur les tissus de voisinage (capsule de Glisson opposée à la zone d'entrée du tir HIFU, tissus rétro péritonéaux rétro hépatiques et diaphragme).

  NB : les éventuelles lésions sur la capsule de Glisson au point d'entrée des ultrasons seront enregistrées mais non considérées comme un critère d'échec.

- la **tolérance** des tirs HIFU,

  ➯ *Objectivée par :* le maintien des constantes hémodynamiques, respiratoires et de la température corporelle du patient (cf. § II.5.a p. 21), pendant la phase de tir et les 5 minutes qui suivent (apprécié par l'anesthésiste).

NB : Des études ancillaires à cette phase II seront réalisées et sont abordées en annexe 2 p.50.

III Méthodologie

III.1 Population

Patients répondant aux critères suivants :

III.1.a Critères d'inclusion

- Homme ou femme âgé(e) de 18 ans ou plus,
- Atteint(e) de métastase(s) hépatique(s) d'un cancer colorectal,

  NB : à titre exceptionnel, l'inclusion de patients porteurs de métastase(s) hépatique(s) d'une autre origine est possible.
- Devant subir une hépatectomie par laparotomie en vue de la résection de métastase(s) hépatique(s), au Centre Léon Bérard,
- Performance status (PS) ECOG ≤ 1,
- Affilié(e) à un régime de sécurité sociale,
- Ayant daté et signé un consentement éclairé.

III.1.b Critères de non-inclusion

- Ayant déjà subi une chirurgie hépatique majeure (plus de trois segments) ou biliaire majeure (contexte de chirurgie hépatique itérative majeure),
- Ayant déjà subi une chirurgie abdominale majeure à l'exclusion d'une chirurgie colorectale pour le traitement de sa tumeur primitive ou exceptionnellement une chirurgie abdominale autre pour le traitement de sa tumeur primitive si celle-ci n'est pas colorectale (la chirurgie de la vésicule biliaire par laparoscopie dans un délai supérieur à 6 mois ne constitue pas un critère de non inclusion),
- Ne pouvant être suivi(e) pour des raisons sociales, familiales, géographiques ou psychologiques,
- Femme enceinte ou allaitante (un test de grossesse sérique ou urinaire doit être négatif au moment de l'inclusion dans l'étude pour les femmes en âge de procréer ; une méthode de contraception fiable devra être utilisée durant la durée de l'étude).

III.1.c Critère d'inclusion spécifique à la phase IIb (hors étape préliminaire)

- Présentant une ou plusieurs métastase(s) hépatique(s) de taille inférieure ou égale à 2 cm devant être réséquée(s) lors de l'hépatectomie objet de la présente étude, et ce que cette résection ait lieu dans le cadre d'une chirurgie en un temps ou bien dans le cadre d'une chirurgie de résection programmée en deux temps.

III.2 Déroulement de l'étude

III.2.a Préalable à la mise en place de l'étude

Les personnels du centre investigateur (chirurgien investigateur et Infirmiers de Bloc Opératoire Diplômés d'Etat (IBODE)) appelés à intervenir dans le cadre de cette recherche auront été formés à l'utilisation du DM par le personnel qualifié de la société EDAP, préalablement à la mise en place de l'étude.

III.2.b Inclusion

Le patient sera inclus par le chirurgien investigateur, soit lors de la visite préalable à l'intervention chirurgicale de résection des métastases hépatiques (dans le mois précédent l'intervention) dans le cas d'une chirurgie en un seul temps ; soit lors de la visite préalable au premier temps chirurgical (réalisation des tirs HIFU) dans le cas d'une chirurgie de résection programmée en deux temps (phase IIb de l'étude seulement). Après avoir identifié un patient éligible (cf. § III.1 p.25), l'Investigateur l'informera sur l'étude, puis lui fera signer un formulaire de consentement.

III.2.c Interventions chirurgicales

Le geste aura lieu au cours de l'intervention chirurgicale (ou au cours du premier temps chirurgical en cas de chirurgie de résection programmée en deux temps), après libération complète du foie, confirmation de l'indication de résection hépatique, déconnection du foie à réséquer et avant réalisation des gestes spécifiques de la résection hépatique. Le chirurgien investigateur appliquera les lésions HIFU sur la partie de foie qu'il est prévu de réséquer.

✓ **Procédure d'anesthésie**

La procédure d'anesthésie sera standardisée pour tous les patients inclus dans l'étude. Elle est présentée en annexe 3 p.50.

La phase de tir ne sera démarrée qu'une fois l'anesthésie stabilisée. Sauf cas d'urgence vitale, aucun des paramètres d'anesthésie ne sera modifié durant la phase d'évaluation (ciblage de la 1ère lésion → 5 min après la dernière lésion).

✓ **Mise en service de l'appareil et préparation de la sonde**

La mise en service de l'appareil au bloc opératoire et la préparation de la sonde seront réalisées par des IBODE préalablement formés, en parallèle de l'intervention chirurgicale et dans la mesure du possible n'impacteront pas la durée d'intervention (cf. Manuel d'utilisation du DM MFOCUS® joint en document annexe).

L'intégralité de l'imagerie échographique acquise durant les traitements sera enregistrée sur un DVD.

✓ **Préparation du patient**

Une voie veineuse centrale ainsi qu'une voie artérielle seront mises en place (procédure non spécifique à l'étude).

Des compresses de protection seront préalablement positionnées sous la zone à traiter par HIFU afin de limiter tout risque de diffusion des ondes ultrasonores et toute lésion des organes avoisinants.

L'abdomen du patient sera rempli de sérum physiologique à 37°C de façon à améliorer le contact acoustique entre la sonde HIFU et le foie. La sonde HIFU sera amenée au contact de la surface du foie par l'intermédiaire d'un liquide de couplage ultrasonore. A partir de l'interface utilisateur et grâce à la sonde échographique intégrée au DM, la région à traiter sera localisée.

NB : Pour limiter le plus possible les risques de lésion des tissus avoisinants, les tirs seront toujours effectués avec un axe de tir évitant tous les organes nobles intra abdominaux.

✓ **Procédure de tir**

⇨ **Phase I**

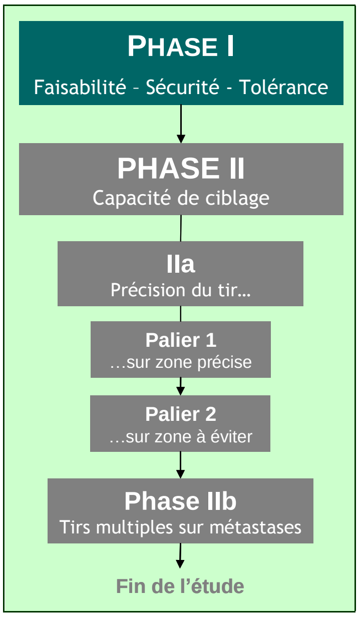

Les tirs porteront sur le foie sain à réséquer, à distance des structures vasculaires importantes et des métastases. Deux tirs HIFU seront réalisés sur chaque patient :

- une lésion superficielle qui débutera au contact de la capsule de Glisson et s'étendra dans le parenchyme hépatique sous-jacent,
- une lésion à une profondeur donnée, supérieure d'au moins 10 mm par rapport aux ablations sous-capsulaires.

  NB : La profondeur du tir sera déterminée pour chaque patient au début de l'intervention, au regard des examens préopératoires (scanner, etc.), de la pièce de foie à réséquer et de la conformation du patient.

⇨ **Phase IIa**

Cette phase aura pour but d'évaluer la précision avec laquelle une lésion HIFU peut être réalisée. Il s'agira, dans un premier temps (palier 1), de placer cette lésion de manière à englober un repère préalablement positionné dans le foie. Dans un deuxième temps (palier 2), il s'agira de démontrer qu'une zone du foie (un vaisseau, un pédicule glissonien…) peut être épargnée. Le repère sera utilisé pour symboliser un vaisseau. L'objectif sera de positionner la lésion HIFU à une distance prédéterminée de ce repère pour attester de la capacité du dispositif MFOCUS à épargner une zone préalablement identifiée.

Deux tirs HIFU seront réalisés sur chaque patient.

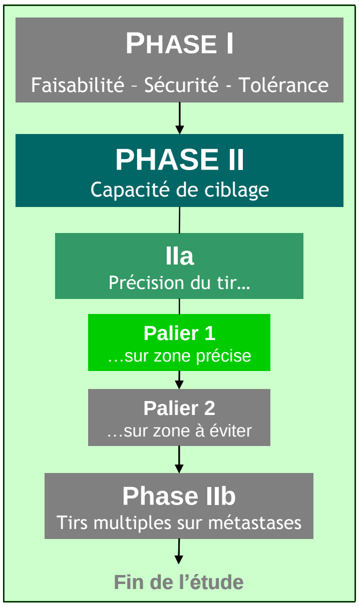

Au palier 1

Pour chacune des lésions un repère stable sera positionné à une profondeur donnée :

- 1ère lésion à la surface du foie,
- 2ème lésion à 20±5 mm de profondeur,

Les tirs seront réalisés en superposant l'épicentre théorique de la lésion et le repère (le repère devra être inclus dans la lésion réalisée).

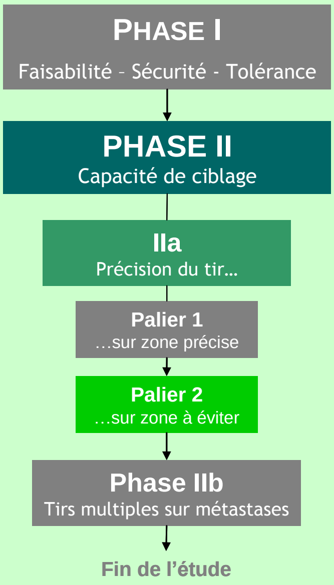

<u>Au palier 2</u>

Les structures vasculaires de référence seront mimées au moyen d'un repère stable.

Les tirs seront réalisés en positionnant le contour théorique de la lésion le plus proximal du vaisseau entre 5 et 10 mm du repère (idéalement 7.5 mm). Il faudra tenir compte des imprécisions de mesures à tous les niveaux de l'expérimentation, comme expliqué ci-après.
La distance à respecter sera fixée de la manière suivante. Le ressort repère est de forme circulaire. Son diamètre est de 5 mm. Selon l'axe de découpe et l'axe de positionnement du repère, l'imprécision varie donc entre 0 et 5 mm (soit 2,5 mm sur le bord du repère). L'analyse des résultats de la phase I a permis de montrer que, lors de la découpe, les valeurs extrêmes du diamètre au milieu des lésions (en regard du point focal) étaient comprises dans une plage de 5 mm (15 à 20 mm pour les lésions en surface et 17 à 22 mm pour les lésions en profondeur). Ainsi, si l'on souhaite conserver une distance comprise entre 5 et 10 mm (idéalement 7,5 mm) entre le bord de la lésion HIFU et le bord du repère, il faudra, compte tenu des incertitudes, observer sur la pièce opératoire une distance comprise entre 1 et 15 mm (idéalement 7,5 mm) comme l'illustre la figure suivante.

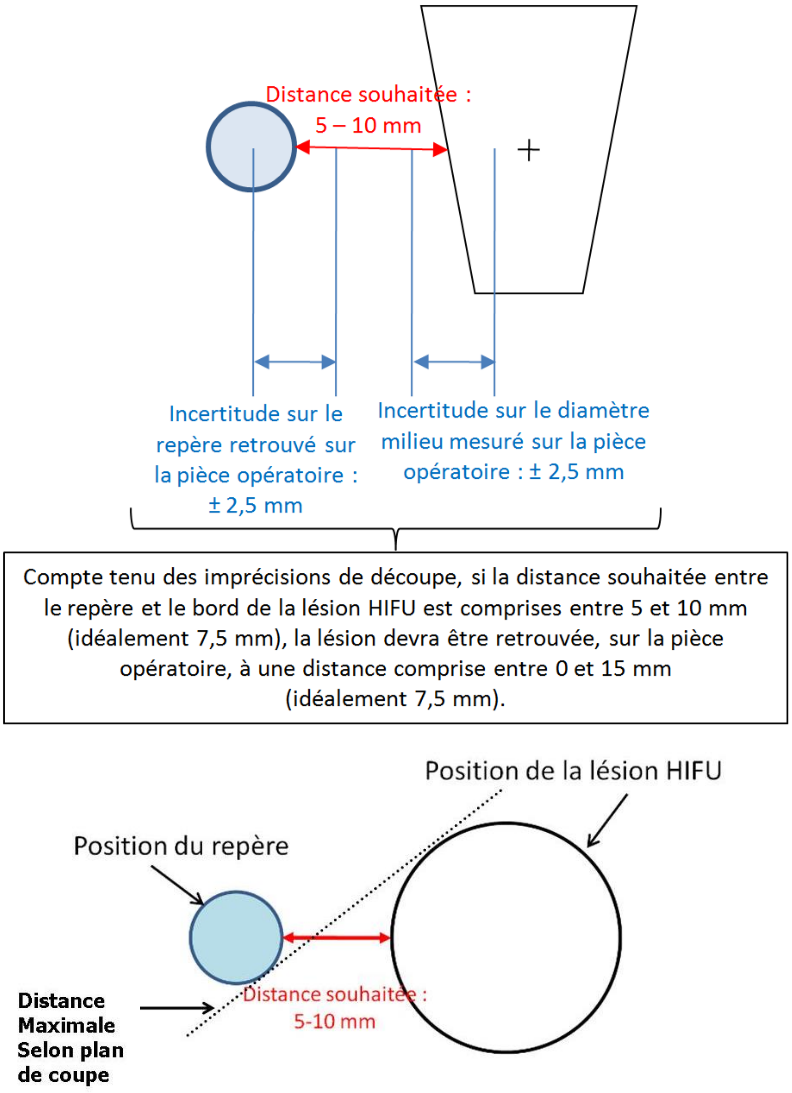

Définition des distances à respecter entre le repère et le bord de la lésion pour valider la capacité de la sonde HIFU à épargner une zone préalablement identifiée (Phase IIa, palier 2). (a) Vue dans l'axe de la découpe. (b) Vue dans un plan perpendiculaire à l'axe de la découpe et au niveau du plan focal.

⇨ **Phase IIb**

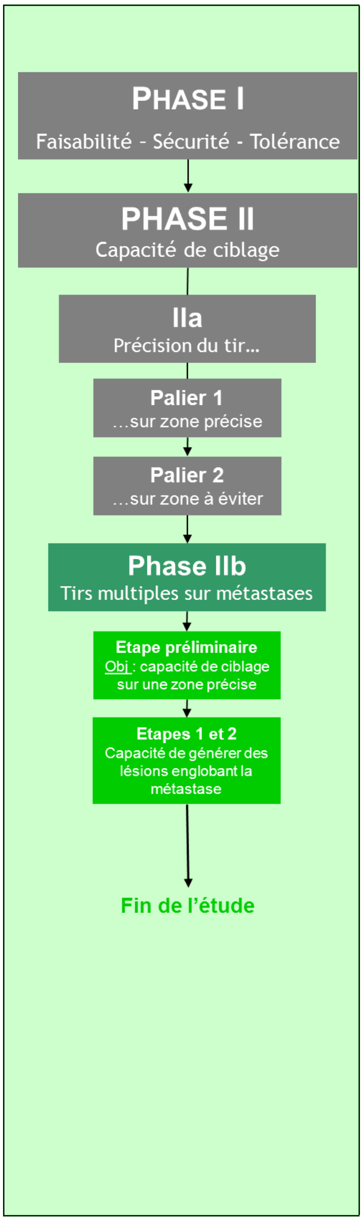

Etape préliminaire : cette étape aura pour but d'évaluer la précision avec laquelle une lésion HIFU peut être réalisée **avec une sonde de traitement à balayage électronique**. Il s'agira de placer cette lésion de manière à englober un repère stable préalablement positionné à la surface du foie. Les tirs seront réalisés en superposant l'épicentre théorique de la lésion et le repère (le repère devra être inclus dans la lésion réalisée).

Remarque : en complément, sera également évaluée dans cette étape préliminaire la capacité à générer des lésions HIFU permettant l'inclusion théorique d'une métastase de 2 cm de diamètre, avec des marges de sécurité de 3 mm. Ce critère complémentaire ne sera pas pris en compte pour le passage aux étapes suivantes de la phase IIb.

Pour la phase IIb proprement dite, les tirs porteront sur les métastases à réséquer de petite taille (≤ 20 mm) et le foie sain péri-lésionnel et seront sommés sur plusieurs plans de manière à générer une lésion englobant la métastase et garantissant une marge de sécurité suffisante dans le tissu sain péri-lésionnel. Pour cette phase qui comprendra deux étapes, deux types de transducteur pourront être utilisés (choix en fonction du type de lésion) :

- **pour les lésions proches des vaisseaux**, les tirs seront réalisés avec une sonde de traitement à balayage électronique doté d'un transducteur classique (sonde utilisée lors de la phase préliminaire)
- **pour les lésions sous capsulaires**, la sonde à balayage électronique sera dotée d'un transducteur torique à croisement intérieur.

Le traitement par HIFU devra suivre une répartition homogène de la taille des métastases ; à ce titre, et dans la mesure du possible (incertitudes de mesure) un tiers des métastases traitées par HIFU auront un diamètre ≤ 10 mm, un tiers auront un diamètre > 10 mm et ≤ 15 mm, et un tiers auront un diamètre > 15 mm et ≤ 20 mm.

Plusieurs métastases pourront être détruites chez un même patient.

✓ <u>**Suites de l'intervention**</u>

A la suite de la réalisation des tirs HIFU, une exploration de l'ensemble de la cavité abdominale sera réalisée afin de détecter d'éventuels effets collatéraux, en particulier des brûlures, sur les organes adjacents au foie.

Après l'application de tirs HIFU, il n'y aura aucune modification en ce qui concerne l'hépatectomie : celle-ci sera en tout point identique à celle qui aurait été réalisée en l'absence de tirs HIFU.

L'analyse anatomopathologique (qui sera réalisée à l'issue de l'intervention chirurgicale ayant conduit à la résection de(s) métastase(s) hépatique(s) et de la zone de traitement par HIFU) comportera en plus une analyse de chacune des lésions HIFU.

III.2.d Suivi postopératoire

Tous les patients inclus seront suivis conformément aux recommandations pour la prise en charge de patients opérés de métastases hépatiques. Dans le cadre de cette étude, les patients seront suivis pendant 30 jours à l'issue de l'intervention chirurgicale ayant conduit à la résection de(s) métastase(s) hépatique(s) et de la zone de traitement par HIFU (même temps opératoire si chirurgie en un temps ou seconde intervention si chirurgie programmée en deux temps pour les patients de la phase IIb).

III.3 Bilans d'évaluation

III.3.a Bilans communs à toutes les étapes

	Inclusion	Peropératoire	Postopératoire *	30 jours postopératoire
• Consentement (daté et signé)	•			
• Test de grossesse & prescription d'une méthode contraceptive fiable si nécessaire (femmes en âge de procréer)	•			
• Scanner préopératoire (avec mesure précise des métastases lors de la phase IIb)	•			
• Examen clinique	•			
• Absence de lésion des tissus avoisinants		•	•	
• Constantes hémodynamiques		•		
• Constantes respiratoires		•		
• Température corporelle		•		

* C'est-à-dire à la suite de la réalisation des tirs HIFU (soit à la fin du premier temps opératoire en cas de chirurgie de résection programmée en 2 temps lors de la phase IIb)*

III.3.b .Phase I

	Inclusion	Peropératoire	Postopératoire	30 jours postopératoire
• Faisabilité des tirs				
• en surface		•		
• en profondeur		•		
• Durées				
• d'intervention supplémentaire nécessaire à la réalisation de 2 tirs HIFU		•		
• de chaque étape du tir (mise en service de l'appareil, préparation de la sonde, repérage du point de tir, réalisation du tir)		•		
• Asepsie		•		
• Capacité à positionner la sonde dans un volume correspondant à 80 % du foie total		•		
• Capacité à :				
• positionner un repère stable		•	•	
• repérer un vaisseau à l'aide d'un repère stable		•	•	
• Faisabilité du monitorage immédiat de l'effet au moyen de l'échographe couplé au DM		•		
• Faisabilité de la description précoce de la lésion en anatomopathologie			•	
• Faisabilité de la mesure de la distance entre le repère / le vaisseau repéré et le bord de la lésion à l'examen anatomopathologique			•	

III.3.c Phase IIa

	Inclusion	Peropératoire	Postopératoire	30 jours postopératoire
• Distance				
• Epicentre de la lésion / repère (palier 1)			•	
• Bord de la lésion et repère matérialisant un vaisseau (palier 2)			•	
• Corrélation entre les dimensions de la lésion (mesurées en insu)				
• Au moyen de l'échographe couplé au DM en peropératoire		•		
• En anatomopathologie immédiatement après l'intervention			•	

III.3.d Phase IIb : étape préliminaire

	Inclusion	Peropératoire	Postopératoire	30 jours postopératoire
• Distance				
• Epicentre de la lésion / repère			•	
• Corrélation entre les dimensions de la lésion (mesurées en insu)				
• Au moyen de l'échographe couplé au DM en peropératoire		•		
• En anatomopathologie immédiatement après l'intervention			•	

III.3.e Phase IIb : étapes 1 et 2

	Inclusion	Peropératoire	Postopératoire *	30 jours postopératoire **
• Nombre de tirs / métastase		•		
• Examen anatomopathologique des lésions générées			•	•

* C'est-à-dire à la suite de la réalisation des tirs HIFU (soit à la fin du premier temps opératoire en cas de chirurgie de résection programmée en 2 temps).

** Soit 30 jours à l'issue de l'intervention chirurgicale ayant conduit à la résection de(s) métastase(s) hépatique(s) et de la zone de traitement par HIFU (même temps opératoire si chirurgie en un temps ou seconde intervention si chirurgie en deux temps pour les patients de la phase IIb).

III.4 Critères de sortie d'étude

Un patient peut être amené à interrompre de façon prématurée l'étude pour diverses raisons. Celles-ci peuvent être :

- Une décision du Promoteur ou de l'Investigateur,
- Le retrait, par le patient, de son consentement ou un refus de traitement,
- L'incapacité du patient à poursuivre l'étude (impossibilité de réaliser l'hépatectomie, contre-indication peropératoire, maladie intercurrente, détérioration clinique significative, raisons administratives…),
- L'absence de nouvelles du patient (perdu de vue),
- Le décès du patient.

Hormis dans les deux derniers cas cités précédemment, une évaluation finale doit être réalisée, établissant les raisons pour lesquelles le patient est sorti de l'étude.

III.5 Arrêt prématuré de l'étude

L'étude pourra être interrompue pour des raisons administratives, ou dans le cas où l'on disposerait de nouvelles données sur la recherche mettant en jeu le rapport bénéfice/risque et/ou sur décision du Promoteur ou des autorités réglementaires.

Si l'étude est arrêtée prématurément ou suspendue, le Promoteur informera immédiatement les Investigateurs, les autorités réglementaires et le CPP de la raison de l'arrêt ou de la suspension. Dans tous les cas, un suivi des patients doit être assuré par l'Investigateur.

IV Vigilance

Le Centre Régional de Pharmacovigilance (CRPV) de Lyon représente le CLB pour la vigilance des essais cliniques dont il est Promoteur.

Cf. - Définitions : annexe 4 p.51.

- Méthodologie d'imputabilité : annexe 5 p.52.

IV.1 Notification des évènements indésirables par l'Investigateur

IV.1.a Notification des événements indésirables dans le cahier d'observation

Tout événement intercurrent, clinique ou biologique, grave ou non, quelle que soit la période dans l'essai, qu'il soit ou non lié à la recherche, doit être noté dans le cahier d'observation aux pages prévues à cet effet.

L'Investigateur notera, pour chaque événement :

- sa description aussi clairement que possible selon la terminologie médicale standard,
- si l'événement est grave ou non,
- la date de fin ou précisera si l'événement est en cours au moment du recueil,
- l'intensité appréciée selon la définition (annexe 4 p.51),
- son évolution (en cas d'événement non fatal, l'évolution devra être suivie jusqu'à la guérison ou retour à l'état antérieur ou à la stabilisation d'éventuelles séquelles),
- la relation de causalité entre cet événement et le DM à l'essai (elle sera appréciée selon les définitions présentées en annexe 5 p.52).

Tous les événements intercurrents, quelle que soit leur sévérité, doivent être suivis par l'Investigateur jusqu'à résolution satisfaisante.

IV.1.b Notification des événements indésirables graves au Promoteur

L'Investigateur informe immédiatement le Promoteur de tous les évènements indésirables graves (EIG) se produisant durant l'étude (à l'exception de ceux mentionnés au § IV.4 p. 35), qu'ils soient jugés imputables ou non à la recherche, au moyen du formulaire de déclaration (annexe 6 p.53).

La déclaration initiale est obligatoirement et rapidement suivie par un ou des rapport(s) complémentaire(s) écrit(s) détaillé(s).

L'investigateur doit déclarer toute réaction ou incident survenant chez une personne qui se prête à la recherche ou chez l'utilisateur du dispositif.

Tout événement indésirable grave survenant pour un participant à la recherche doit être déclaré:

- A partir de la date de signature du consentement,
- Jusqu'à 30 jours après l'intervention chirurgicale ayant conduit à la résection de(s) métastase(s) hépatique(s)(soit jusqu'à la fin du suivi prévu dans l'étude),
- Sans limitation de durée si l'investigateur juge que l'événement est susceptible d'être dû à la présente expérimentation.

PROCEDURE DE NOTIFICATION :

① **L'Investigateur documente le plus précisément possible, date et signe le formulaire de notification d'un évènement indésirable grave** (annexe 6 p.53).

Pour être recevable, ce formulaire doit contenir au moins :

- un patient identifié par son numéro de code dans la recherche (ou un utilisateur),
- un notificateur,
- la description de la réaction et un diagnostic médical si possible et toute information pertinente pour l'évaluation de l'événement.

② **L'investigateur indique sur la fiche de déclaration son évaluation du lien de causalité** (annexe 6 p.53) **de l'événement avec :**

- le DM expérimental : sonde HIFU torique MFOCUS®,
- ou le geste de mise en œuvre du DM expérimental, par exemple la mise en place de la sonde ou toute manipulation du matériel (à préciser),
- ou avec l'intervention chirurgicale,
- ou avec les traitements concomitants,
- ou avec une pathologie sous-jacente (à préciser),
- ou avec une autre cause (à préciser).

③ **Il le faxe immédiatement au CRPV de Lyon (🖷 04 72 11 69 85).**

Les différents bilans et comptes-rendus (d'hospitalisation, d'imagerie, etc.) nécessaires à l'évaluation de l'événement sont joints à la fiche de déclaration initiale ou transmis dans les meilleurs délais. Tous ces documents doivent être anonymisés.

En cas de données manquantes nécessaires à l'évaluation du cas, un complément d'information sera demandé par le CRPV à l'Investigateur. Celui-ci devra adresser les différents éléments au CRPV dans les 48 heures ou dans les meilleurs délais.

De même, l'Investigateur :

- fait part au CRPV de tout fait nouveau de sécurité, et lui adresse les informations complémentaires et les données de suivi concernant ce fait nouveau.
- est responsable du suivi médical approprié des patients jusqu'à la résolution ou la stabilisation de l'effet ou jusqu'au décès du patient.
- transmet dans les 48h les informations de suivi au CRPV à l'aide d'un formulaire de déclaration d'événement indésirable grave (en cochant la case Suivi n°X pour préciser qu'il s'agit d'un suivi et non d'un rapport initial). Il transmet également le dernier suivi à la résolution ou à la stabilisation de l'événement.
- conserve les documents concernant l'événement indésirable afin de permettre, en cas de nécessité de compléter les informations précédemment transmises.

IV.1.c Notification des grossesses au Promoteur

L'Investigateur informe immédiatement le Promoteur de toute grossesse survenant au cours de l'étude. Il effectue un suivi de chaque grossesse jusqu'à son terme et informe le Promoteur de l'évolution de la grossesse.

L'Investigateur pourra utiliser la fiche de déclaration d'événement indésirable en précisant les données sur la patiente, sur les traitements (DM expérimental, traitements associés et habituels), la datation de la grossesse, le lieu du suivi de la grossesse et de l'accouchement.

IV.2 Évènements indésirables ne nécessitant pas une déclaration immédiate

Événements non graves : hospitalisations durant moins de 24 heures.

Événements non liés à l'utilisation du DM expérimental : hospitalisations programmées préalablement au début de l'étude.

NB : Seuls ces événements ne font pas l'objet d'une déclaration immédiate au Promoteur, ils doivent être notés dans le cahier de recueil de données du patient.

IV.3 Évènements indésirables imputables à la pathologie ou à la chirurgie

Événements indésirables graves liés au cancer colorectal métastatique : hémorragie digestive en rapport avec l'anastomose, abcès péri anastomotique, fistule digestive, iléus postopératoire prolongé…

Complications inhérentes à toute chirurgie de résection de MH : hémorragie de la tranche d'hépatectomie, insuffisance hépatique clinique ou biologique (augmentation de la bilirubine au-dessus de 50 µmol/L et diminution du taux de prothrombine en dessous de 50 % au-delà du cinquième jour postopératoire), ascite, ictère, signe d'hypertension portale, fistule biliaire prolongée…

IV.4 Effets indésirables attendus - Définition du document de référence

Sont considérés comme effets indésirables attendus, les effets indésirables figurant dans le document de référence du DM expérimental.

Ce DM ne fait pas l'objet d'un marquage CE. Le document de référence est donc constitué des documents relatifs au DM expérimental MFOCUS® :

- Compte rendu des premiers essais précliniques avec une sonde HIFU torique, Juin 2009 ;
- Manuel d'utilisation du prototype MFOCUS® version EDP 800 548 A, Juillet 2009 ;
- Analyse des risques - traitement HIFU métastases foie - projet EDP 050, Juillet 2009.

Compte tenu de ces données, les risques potentiels identifiés avec le traitement HIFU et donc les effets indésirables attendus du DM sont les suivants :

- Pour le patient : risque de contamination lors de l'installation de la sonde
- Pour le patient : risque de brûlure des organes adjacents lors des tirs HIFU
- Pour le personnel / utilisateur : risque de blessure suite à une chute du support (2 kg)

Par ailleurs, lors des études précliniques chez l'animal, un cas de fuite biliaire avec péritonite due à des tirs répétés au niveau du pédicule hépatique a été observé. NB : cette zone ne sera pas traitée dans le cadre du présent essai.

IV.5 Archivage des déclarations d'événement indésirable

L'original des déclarations d'événement indésirable complété par l'Investigateur est vérifié par l'Attaché de Recherche Clinique (ARC) lors des visites de monitoring afin de s'assurer de la conformité avec les données du dossier médical et avec le formulaire faxé préalablement au Centre de Pharmacovigilance.

Les déclarations d'événements indésirables et les documents s'y rapportant sont classés par le Promoteur suivant ses procédures.

Une copie est conservée par l'Investigateur dans le cahier de recueil de données (CRF).

V Méthodologie statistique

V.1 Nombre de participants et règles statistiques

Cette étude prévoit d'inclure 35 à 44 patients, soit :

V.1.a Phase I

Un **effectif de 6 patients** sera inclus, permettant la réalisation d'une analyse de type séquentielle en 2 étapes selon les critères définis par Lee (27). Cette analyse a pour but d'arrêter précocement l'étude si un taux d'échec > 15 % est observé.

<u>**REGLES STATISTIQUES :**</u>

Un échec sera défini par la survenue d'au moins une des situations suivantes :

- une durée de réalisation des 2 lésions impactant la durée de réalisation de l'intervention chirurgicale de plus de 30 minutes,
- l'impossibilité de réaliser un tir en surface et/ou en profondeur,
- la survenue d'une faute d'asepsie lors de la mise en place du DM,
- la survenue d'une lésion accidentelle sur les tissus de voisinage,
- l'altération significative d'une ou plusieurs constante(s) hémodynamique(s) ou respiratoire(s) (cf. § II.5.a p. 21) (appréciée par l'anesthésiste).

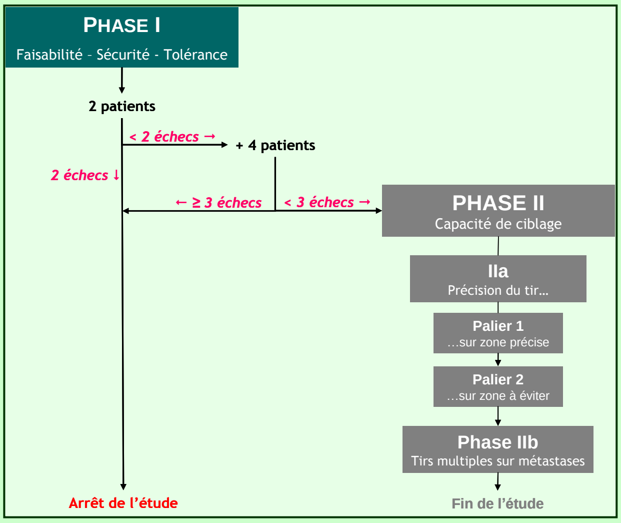

Le passage à la phase II nécessitera la validation de la règle suivante :

- 1ère analyse après 2 patients :
  - si < 2 échecs sont constatés ➡ poursuite de l'étude et inclusion de 4 patients supplémentaires,
  - si 2 échecs ➡ arrêt de l'étude.
- 2ème analyse après 6 patients :
  - si < 3 échecs sont constatés ➡ poursuite de l'étude et passage en phase II (après accord du Comité de pilotage),
  - si ≥ 3 échecs ➡ arrêt de l'étude.

<u>NB :</u> A l'issue de la phase I, le Comité de Pilotage de l'étude (cf. § VII.1 p.41) se réunira pour examiner l'ensemble des données colligées, valider ou non les critères de poursuite de l'étude et définir plus précisément la méthodologie de la phase II.

V.1.b Phase II

<u>**Phase IIa**</u>

Un **effectif de 6 à 12 patients** sera inclus en 2 paliers successifs comprenant chacun 3 à 6 patients. Cet effectif a été déterminé en utilisant la méthodologie classiquement utilisée dans les protocoles d'escalade de doses en recherche clinique. Il s'agit d'être capable de mettre en évidence une insuffisance de précision des tirs HIFU si cet événement survient avec une fréquence élevée. En utilisant la loi binomiale, des effectifs de respectivement 3 et 6 patients permettent ainsi de garantir une probabilité de 90 % d'observer au moins un patient avec une précision de tir inacceptable, si cet événement se produit respectivement dans au moins 54 % et 32 % des cas.

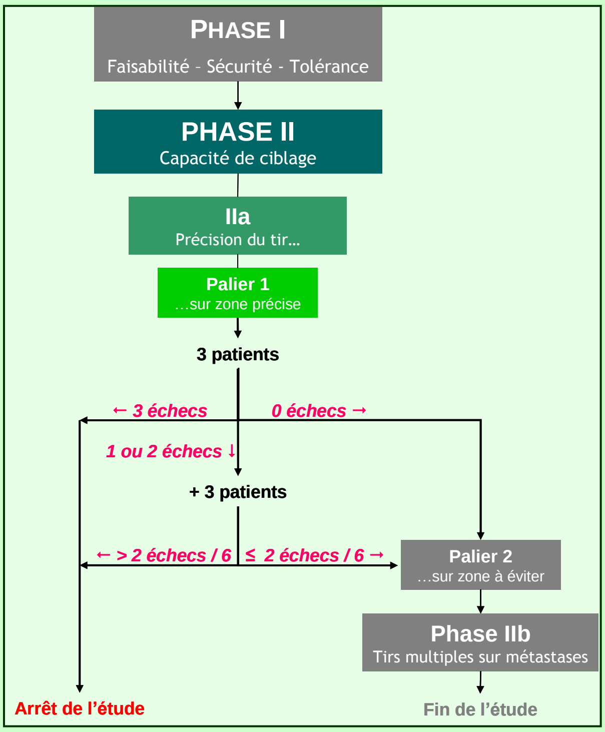

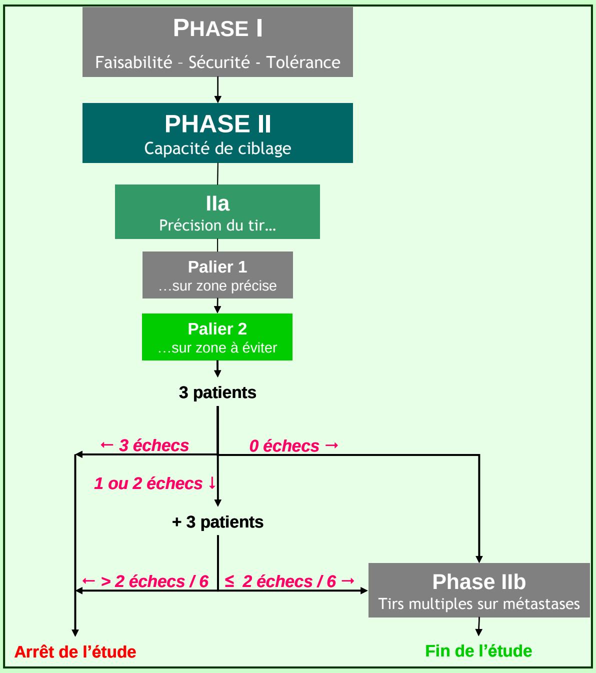

- **1er palier** (3 à 6 patients) :

Pour un patient et 2 tirs par patient, l'intervention est définie comme un succès si la moyenne des distances mesurées entre le bord de la lésion le plus proximal du repère et le repère lui-même est strictement inférieure à 5 mm. Dans le cas contraire, l'intervention sera notifiée en échec.

Trois patients seront inclus à ce 1er palier :

- Si aucun patient ne présente un échec ➡ passage au 2ème palier ;
- Si 1 ou 2 patients présentent un échec ➡ inclusion de 3 patients supplémentaires au 1er palier

  ↳Si > 2 échecs sur les 6 patients ➡ arrêt de l'étude,

  ↳Si ≤ 2 échecs sur les 6 patients ➡ passage au 2e palier;
- Si 3 patients présentent un échec ➡ arrêt de l'étude.

- **2ème palier** (3 à 6 patients) :

Pour un patient et 2 tirs par patient, l'intervention est définie comme un succès si la moyenne des distances mesurées entre le bord de la lésion le plus proximal du repère et le bord du repère lui-même est supérieure ou égale à 1 mm et strictement inférieure à 15 mm (idéalement 7,5 mm). Dans le cas contraire, l'intervention sera notifiée en échec.

Trois patients seront inclus à ce 2ème palier :

- Si aucun patient ne présente un échec ➡ passage à la phase IIb de l'étude ;
- Si 1 ou 2 patients présentent un échec ➡ inclusion de 3 patients supplémentaires au 2ème palier

  ↳Si > 2 échecs sur les 6 patients ➡ arrêt de l'étude,

  ↳Si ≤ 2 échecs sur les 6 patients ➡ passage en phase IIb ;
- Si 3 patients présentent un échec ➡ arrêt de l'étude.

NB : A l'issue de la phase IIa, le Comité de Pilotage de l'étude (cf. § VII.1 p.41) se réunira pour examiner l'ensemble des données colligées, valider ou non les critères de poursuite de l'étude et définir plus précisément la méthodologie de la phase IIb.

Phase IIb

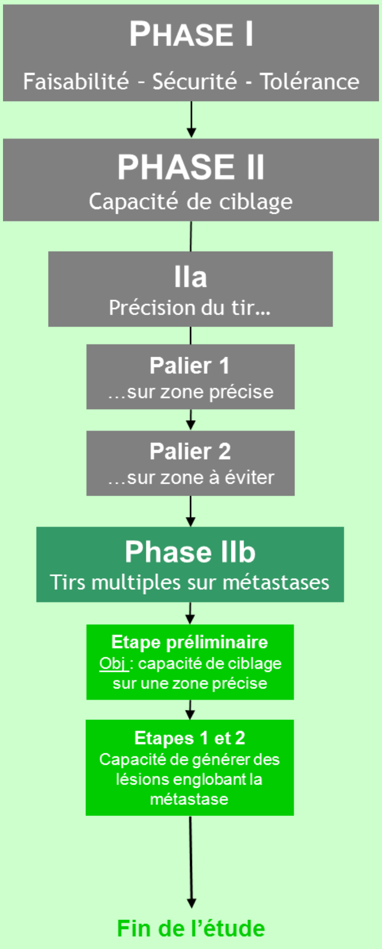

- Etape préliminaire (3 à 6 patients) :

Pour un patient et avec 1 à 2 lésions par patient, l'intervention est définie comme un succès si la moyenne des distances mesurées entre le bord de la lésion le plus proximal du repère et le repère lui-même est strictement supérieure à 5 mm. Dans le cas contraire, l'intervention sera notifiée en échec.

Trois patients seront initialement inclus à cette étape :

- Si aucun patient ne présente un échec ➡ passage à la phase IIb proprement dite (étapes 1 et 2) ;
- Si 1 ou 2 patients présentent un échec ➡ inclusion de 3 patients supplémentaires à cette étape préliminaire :
  - ↳Si > 2 échecs sur les 6 patients ➡ arrêt de l'étude,
  - ↳Si ≤ 2 échecs sur les 6 patients ➡ passage à la phase IIb proprement dite (étapes 1 et 2) ;
- Si 3 patients présentent un échec ➡ arrêt de l'étude.

Remarque : en complément, sera également évaluée dans cette étape préliminaire la capacité à générer des lésions HIFU permettant l'inclusion théorique d'une métastase de 2 cm de diamètre, avec des marges de sécurité de 3 mm. Ce critère complémentaire ne sera pas pris en compte pour le passage aux étapes suivantes de la phase IIb.

- Etapes 1 et 2 :

Un effectif d'une vingtaine de patients sera inclus dans cette phase. Cet effectif a été déterminé de la façon suivante :

- l'unité statistique pour l'analyse est la métastase,
- le nombre de métastase à cibler par HIFU a été déterminé en utilisant un plan expérimental de Simon (Optimal design) développée en oncologie pour les plans d'essais de phase II en deux étapes (28).

  Un succès est défini par la réalisation d'une lésion qui englobe la métastase en garantissant (avec un nombre maximum de 15 tirs) :

  a) macroscopiquement, une marge de sécurité supérieure ou égale à 3 mm en tout point (avec une incertitude de mesure de +/- 1 mm) , **et**

  b) microscopiquement, des marges non nulles dans les plans non évalués macroscopiquement.

Les hypothèses suivantes ont été utilisées, où P est la probabilité de succès :

- p_0 = **0,70** est la valeur limite supérieure du taux de succès qui, s'il est avéré, traduirait l'inefficacité de tirs HIFU multiples sur les métastases à réséquer et le foie sain péri-lésionnel.
- p_1 = **0,90** est la valeur limite inférieure du taux de succès qui traduirait l'efficacité de tirs HIFU multiples sur les métastases à réséquer et le foie sain péri-lésionnel.

Avec un risque d'erreur alpha égal à 5 % et une puissance de 80 %, 27 métastases devront être ciblées par HIFU pour pouvoir rejeter H_0: $P \leq p_0$ *versus* H_1: $P \geq p_1$ en situation unilatérale. En considérant

	qu'en moyenne 1,5 métastases seront ciblées sur chaque patient inclus, avec un maximum de 3 métastases par patient, un effectif d'environ 20 patients sera inclus.<br>Ces inclusions se feront en 2 étapes consécutives. Dans une première étape, un minimum de 5 succès devra être observé parmi les 6 premières métastases traitées pour poursuivre les inclusions dans une seconde étape.<br>Dans ce cas, 21 métastases supplémentaires seront traitées dans une seconde étape jusqu'à un total de 27 métastases.<br>Pour ces 2 étapes, une sonde de traitement à balayage électronique sera utilisée et sera associée à un transducteur (2 types possibles). Le choix du transducteur se fera en fonction du type de lésion : utilisation d'un transducteur classique pour les lésions proches des vaisseaux ou utilisation d'un transducteur torique à croisement intérieur pour les lésions sous capsulaires.<br>A la fin de la deuxième étape, un nombre de succès observé supérieur ou égal à 23 permettra de valider l'étape IIb et de conclure au rejet de H_0 pour accepter H_1.

V.2 Définition des populations d'analyse

Pour chacune des phases de l'étude, les analyses du critère de jugement principal et des critères de jugement secondaires seront réalisées sur la population évaluable pour la faisabilité clinique, la tolérance et l'efficacité du ciblage des MH par HIFU. Cette population comprendra tous les patients inclus dans l'étude et pour lesquels une hépatectomie aura pu être réalisée. Seuls les patients pour lesquels l'hépatectomie n'aura pas pu être réalisée seront exclus des analyses.

V.3 Analyses

Les analyses seront réalisées sur une base descriptive. Les variables qualitatives seront décrites par leurs distributions de fréquences et les pourcentages correspondants avec leur intervalle de confiance à 95 %, les variables quantitatives par leur nombre d'occurrence, moyenne, écart-type, médiane, valeurs minimum et maximum.

VI Résultats attendus

La démonstration de la faisabilité clinique, de la bonne tolérance et de l'efficacité du ciblage des MH par HIFU constituera une première étape indispensable avant d'utiliser cette nouvelle technologie pour des applications à visée curative et pour des applications extracorporelles.

VII Gestion et coordination de l’essai

VII.1 Pilotage

Un Comité de pilotage sera constitué avant le début de l’étude.

Les résultats des analyses intermédiaires obtenus à l’issue de chacune des phases de l’étude seront communiqués au Comité de pilotage de l’étude. Ce Comité se réunira pour prendre les décisions nécessaires quant à la poursuite de l’étude au fur et à mesure de son déroulement, en accord avec le Promoteur.

VII.2 Recueil de données

Pour chaque patient, l’ensemble des données recueillies par l’Investigateur, assisté des IBODE et de l’Assistant d’Essai Clinique (AEC) désignés pour cette tâche, sera colligé dans un cahier de recueil de données (CRF). Les formulaires seront transmis régulièrement au Centre de Coordination.

NB : Pour la bonne conduite de cette étude, il est impératif de suivre les procédures et instructions de remplissage notifiées sur le CRF.

VII.3 Contrôle qualité des données

Le contrôle de la qualité des données sera assuré sur site par l’Attaché de Recherche Clinique (ARC) du Centre de Coordination lors des visites de monitoring.

NB : pour la phase I, le contrôle qualité sera centré sur la validation des critères d’éligibilité et le recueil des EIG.

VII.4 Traitement informatique

L'infogérance de l'ensemble des données recueillies sur les bordereaux prévus à cet effet sera traitée par un Technicien de Recherche Clinique (TRC) du Centre de Coordination. Une liste de contrôles de cohérence sera définie et appliquée à l'ensemble des bordereaux saisis. Ces contrôles génèreront des demandes de corrections destinées aux Investigateurs. Les contrôles seront activés jusqu'à ce qu'aucune demande de correction ne soit générée.

VII.5 Analyses statistiques

Les analyses statistiques seront réalisées en fin d'étude, après le gel de la base de données (contrôle et saisie des corrections effectués).

VII.6 Rédaction du rapport d’étude

Le rapport de l’analyse finale de l’étude sera remis par le Chef de projet et le Responsable statistique à l’Investigateur coordonnateur au maximum 6 mois après la clôture de l’étude (gel de la base de données).

VII.7 Publications

L'Investigateur coordonnateur s'engage à publier les résultats de l'étude. Aucune publication (abstract, poster, communication, manuscrit, etc.) ne peut être effectuée sans son accord.

<u>REGLES DE PUBLICATION :</u>

Toute publication ou communication (orale ou écrite) sera décidée d'un commun accord entre l'Investigateur coordonnateur et le Responsable du Centre de Coordination (DRCI) et respectera les recommandations internationales (http://www.icmje.org/).

Seront cités parmi les auteurs les personnes ayant participé activement à l'élaboration du protocole de l'étude, à son déroulement et à la rédaction des résultats. Ils seront les premiers signataires. Parmi ceux-ci :

- Le(s) Investigateur(s) ayant inclus et suivi des malades seront tous cités. Si nécessaire, l'ordre de citation sera fonction du nombre d'inclusions dans l'étude,
- Les membres de la DRCI ayant participé à l'élaboration du protocole, ceux qui prendront en charge l'analyse des données et l'aide à la rédaction des articles scientifiques figureront parmi les auteurs,
- Les membres de l'INSERM ayant participé à la mise en œuvre de la présente étude figureront également parmi les auteurs,

<u>NB :</u>

- Le Centre Léon Bérard sera cité comme Promoteur de l'étude et remercié,
- La société EDAP-TMS figurera dans la liste des remerciements,
- Les financeurs figureront également dans la liste des remerciements.

VIII Aspects éthiques et règlementaires

L'étude sera conduite conformément :

- aux principes éthiques de la déclaration d'Helsinki,
- aux Bonnes Pratiques Cliniques de la Conférence Internationale d'Harmonisation (ICH-E6, 17/07/96),
- à la Directive Européenne (2001/20/CE) sur la conduite des études cliniques,
- à la Loi de Santé Publique (n°2004-806) du 9 août 2004,
- à la Loi Informatique et Libertés n°78-17 du 6 janvier 1978 modifiée par la loi n°2004-801 du 6 août 2004 relative à la protection des personnes physiques à l'égard des traitements de données à caractères personnel,
- à la Loi bioéthique n°2004-800 du 6 août 2004.

VIII.1 Instances impliquées dans l'étude

VIII.1.a Comité de Protection des Personnes

Ce protocole a reçu l'avis favorable du Comité de Protection des Personnes (CPP) Sud-Est IV le 22/09/2009 (annexe 7 p.55).

VIII.1.b Autorité compétente

La conduite de ce protocole a été autorisée par l'Agence Nationale de Sécurité du Médicament et des produits de santé (ANSM – ex-AFSSAPS-) le 23/09/2009 (annexe 8 p.57).

VIII.1.c Comité Consultatif sur le Traitement de l'Information en matière de Recherche dans le domaine de la Santé - Commission Nationale de l'Informatique et des Libertés

L'UBET (DRCI) a obtenu un avis favorable de conformité à la Méthodologie de référence MR-01 pour le dossier enregistré sous le numéro :

- 00.S.018 pour le Comité Consultatif sur le Traitement de l'Information en matière de Recherche dans le domaine de la Santé (CCTIRS),
- 00-1142 pour la Commission Nationale de l'Informatique et des Libertés (CNIL).

VIII.2 Assurance

Conformément aux dispositions règlementaires de la Loi 2004-806 du 9 août 2004 (décret 2006-477 du 26 avril 2006), le Promoteur a souscrit une police d'assurance pour couvrir les conséquences pécuniaires des sinistres trouvant leur cause génératrice dans la présente recherche biomédicale (annexe 9 p.58).

VIII.3 Confidentialité

Conformément à l'article R.5120 du Code de la Santé Publique, toutes les personnes appelées à collaborer à l'étude sont tenues au secret professionnel en ce qui concerne notamment la nature des produits utilisés, l'étude, les personnes qui s'y prêtent et les résultats obtenus.

Toute information relative à cette étude, et n'ayant fait l'objet d'aucune publication antérieure, est considérée comme une information confidentielle et privée. Ces informations confidentielles resteront la seule et unique propriété du Promoteur, elles ne sauraient être divulguées à des tiers sans le consentement écrit préalable du Promoteur. Elles ne devront être utilisées que dans le cadre de l'exécution de cette étude.

VIII.4 Audits et inspections

VIII.4.a Audits par le Centre de Coordination

L'étude pourra être auditée par des spécialistes proposés par le Centre de coordination. Au cours de ces audits, l'Investigateur et son équipe se rendent disponibles, leur autorisent l'accès au plateau technique, au matériel de l'étude ainsi qu'aux dossiers des patients (accès direct).

VIII.4.b Inspections par les autorités de santé

Lors d'une éventuelle inspection par les autorités de santé, les points suivants peuvent être contrôlés :

- l'organisation générale de l'étude,
- la qualification du personnel chargé de sa réalisation,
- la qualité des équipements,
- les formulaires de consentement éclairé,
- l'obtention de l'avis du CPP,
- les modalités de dispensation et de stockage des produits,
- la conduite de l'étude (données recueillies, etc.),
- l'archivage des documents relatifs à l'étude.

IX Références bibliographiques

1. Manfredi S, Lepage C, Hatem C, et al.: Epidemiology and management of liver metastases from colorectal cancer. Ann Surg 244:254-259, 2006

2. Bismuth H, Adam R, Levi F, et al.: Resection of nonresectable liver metastases from colorectal cancer after neoadjuvant chemotherapy. Ann Surg 224:509-520, 1996

3. Mulier S: Local recurrence after hepatic radiofrequency coagulation: multivariate meta-analysis and review of contributing factors. 2005

4. Rivoire M: Combination of neoadjuvant chemotherapy with cryotherapy and surgical resection for the treatment of unresectable liver metastases from colorectal carcinoma. 2002

5. Antoniou A, Lovegrove RE, Tilney HS, et al.: Meta-analysis of clinical outcome after first and second liver resection for colorectal metastases. Surgery 141:9-18, 2007

6. Pawlik TM, Schulick RD, Choti MA: Expanding criteria for resectability of colorectal liver metastases. Oncologist 13:51-64, 2008

7. Garcea G, Lloyd TD, Aylott C, et al.: The emergent role of focal liver ablation techniques in the treatment of primary and secondary liver tumours. Eur J Cancer 39:2150-2164, 2003

8. Seifert JK: Indicators of recurrence following cryotherapy for hepatic metastases from colorectal cancer. 1999

9. Coleman DJ, Lizzi FL, Driller J, et al.: Therapeutic ultrasound in the treatment of glaucoma. II. Clinical applications. Ophthalmology 92:347-353, 1985

10. Valtot F, Kopel J, Petit E, et al.: [Treatment of refractory glaucoma with high density focused ultrasonics]. J Fr Ophtalmol 18:3-12, 1995

11. Chapelon JY, Margonari J, Vernier F, et al.: In vivo effects of high-intensity ultrasound on prostatic adenocarcinoma Dunning R3327. Cancer Res 52:6353-6357, 1992

12. Poissonnier L, Chapelon JY, Rouviere O, et al.: Control of prostate cancer by transrectal HIFU in 227 patients. Eur Urol 51:381-387, 2007

13. Kinsey AM, Diederich CJ, Rieke V, et al.: Transurethral ultrasound applicators with dynamic multi-sector control for prostate thermal therapy: in vivo evaluation under MR guidance. Med Phys 35:2081-2093, 2008

14. Madersbacher S, Pedevilla M, Vingers L, et al.: Effect of high-intensity focused ultrasound on human prostate cancer in vivo. Cancer Res 55:3346-3351, 1995

15. Vallancien G, Harouni M, Guillonneau B, et al.: Ablation of superficial bladder tumors with focused extracorporeal pyrotherapy. Urology 47:204-207, 1996

16. Wu F, Wang ZB, Cao YD, et al.: "Wide local ablation" of localized breast cancer using high intensity focused ultrasound. J Surg Oncol 96:130-136, 2007

17. Melodelima D, Prat F, Fritsch J, et al.: Treatment of esophageal tumors using high intensity intraluminal ultrasound: first clinical results. J Transl Med 6:28, 2008

18. Wu F, Wang ZB, Chen WZ, et al.: Extracorporeal high intensity focused ultrasound ablation in the treatment of patients with large hepatocellular carcinoma. Ann Surg Oncol 11:1061-1069, 2004

19. Vaezy S, Fujimoto VY, Walker C, et al.: Treatment of uterine fibroid tumors in a nude mouse model using high-intensity focused ultrasound. Am J Obstet Gynecol 183:6-11, 2000

20. Marquet F, Pernot M, Aubry JF, et al.: Non-invasive transcranial ultrasound therapy based on a 3D CT scan: protocol validation and in vitro results. Phys Med Biol 54:2597-2613, 2009

21. Uchida T, Ohkusa H, Nagata Y, et al.: Treatment of localized prostate cancer using high-intensity focused ultrasound. BJU Int 97:56-61, 2006

22. Melodelima D: Toric HIFU transducer for large thermal ablation. 2007

23. Melodelima D, N'Djin WA, Parmentier H, et al.: Ultrasound surgery with a toric transducer allows the treatment of large volumes over short periods of time. Applied Physics Letters 91:193901, 2007

24. Melodelima D: Thermal ablation by high-intensity-focused ultrasound using a toroid transducer increases the coagulated volume. Results of animal experiments. 2009

25. Parmentier H: High-Intensity Focused Ultrasound Ablation for the Treatment of Colorectal Liver Metastases During an Open Procedure: Study on the Pig. 2009

26. N'Djin WA: Utility of a tumor-mimic model for the evaluation of the accuracy of HIFU treatments. results of in vitro experiments in the liver. 2008

27. Lee YJ, Wesley RA: Statistical contributions to phase II trials in cancer: interpretation, analysis and design. Semin Oncol 8:403-416, 1981

28. Simon R. Optimal two-stage designs for phase II clinical trials. Controlled Clin Trials 1989;10:1-10.

Annexes

**Annexe 1**	Spécifications techniques du repère utilisé	p.48
**Annexe 2**	Etudes ancillaires	p.50
**Annexe 3**	Procédure d'anesthésie	p.50
**Annexe 4**	Matériovigilance – Définitions	p.51
**Annexe 5**	Matériovigilance – Méthodologie d'imputabilité	p.52
**Annexe 6**	Formulaire de déclaration d'Evènement Indésirable Grave	p.53
**Annexe 7**	Avis favorable du CPP	p.55
**Annexe 8**	Autorisation de l'AFSSAPS (ANSM)	p.57
**Annexe 9**	Attestation d'assurance	p.58

Annexe 1 Spécifications techniques du repère utilisé

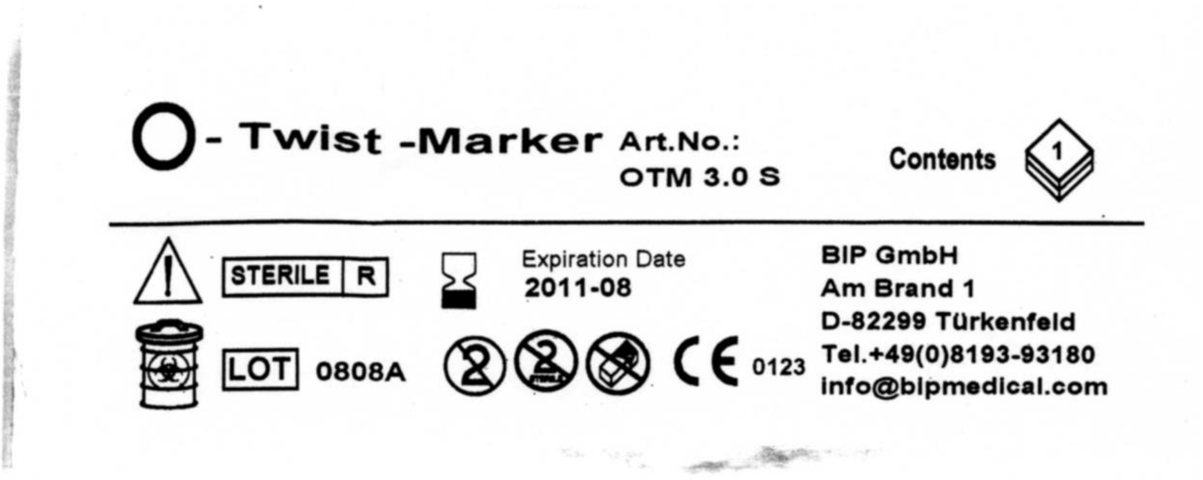

Annexe 2 Etudes ancillaires

Il s'agira d'évaluer, lors de la seconde phase de cette étude, la **corrélation entre l'imagerie per-opératoire** (échographie) **et l'imagerie fonctionnelle** (scanner),

- *Objectivé par :* la confrontation entre les données de l'échographie per opératoire, de l'examen macroscopique post opératoire et d'une imagerie par scanner ou IRM réalisée immédiatement après l'ablation de la pièce et avant sa transmission au laboratoire d'anatomopathologie.

Annexe 3 Procédure d'anesthésie

- *Prémédication*
    - Atarax : 100mg,

Pas de préparation digestive.

- *Induction anesthésique*
    - Propofol : 2 mg/kg,
    - Remifentanil anesthésie intraveineuse à objectif de concentration (AIVOC) model Minto Ce 4,
    - Cis atracurium : 0.15 mg/kg,
    - Kétamine : 0,15 mg/kg,

Remplissage vasculaire Ringer Lactate : 4 mL/kg/h,

Intubation ventilation standardisée : volume courant (VT) : 8 mL/kg, fréquence respiratoire (FR) : 10 resp/min.

- *Entretien*
    - Remifentanil QS (en général Ce 3),
    - Cis atracurium : en fonction du remplissage 4 mL/kg/h,
    - Sévoflurane QS BIS entre 40 et 60.

- *Prévention de la douleur post opératoire*

45 mn avant la fin de la chirurgie :

- Morphine : 0.15 mg/kg, puis PCA en Salle de Surveillance Post Interventionnel (SSPI) et dans le service
- Paracétamol : 1 g,
- Acupan : 20 mg.

Annexe 4 Matériovigilance - Définitions

- **Événement indésirable** : toute manifestation nocive survenant chez une personne qui se prête à une recherche biomédicale, que cette manifestation soit liée ou non à la recherche ou au DM sur lequel porte cette recherche.

- **Effet indésirable de la recherche ou du DM expérimental** : toute réaction nocive survenant et non désirée due à la recherche ou au DM expérimental.

- **Événement ou Effet Indésirable Grave** (EIG) : tout évènement ou tout effet indésirable qui, quelle que soit la dose administrée :
  - entraîne le décès ;
  - met en jeu le pronostic vital ;
  - entraîne une hospitalisation ou une prolongation d'hospitalisation ;
  - provoque une invalidité permanente ou une incapacité temporaire grave ;
  - se traduit par une anomalie ou une malformation congénitale,
  - est jugé médicalement significatif (i.e. a nécessité un acte pour éviter une évolution vers une gravité supérieure).

  NB : la confusion est fréquente entre gravité et événement : l'hospitalisation n'est pas un événement indésirable grave à elle seule mais elle est motivée par un événement indésirable grave.

- **Effet indésirable inattendu** : pour une recherche portant sur un DM, tout effet indésirable du DM dont la nature, la sévérité ou l'évolution ne concorde pas avec les informations figurant dans la documentation technique du DM

- **Fait nouveau** : toute nouvelle donnée de sécurité pouvant conduire à une réévaluation du rapport des bénéfices et des risques de la recherche ou du DM faisant l'objet de la recherche, ou qui pourrait être suffisant pour envisager des modifications dans l'utilisation du DM, dans la conduite de la recherche, ou des documents relatifs à la recherche. Il s'agit notamment de toute augmentation cliniquement significative de la fréquence d'apparition d'un effet indésirable grave inattendu, des suspicions d'effets indésirables graves (EIGI) survenus chez des participants ayant terminé l'essai, tout fait nouveau concernant l'essai clinique ou l'utilisation du DM lorsqu'il est susceptible de porter atteinte à la sécurité des participants.

Annexe 5 Matériovigilance – Méthodologie d'imputabilité

L'imputabilité est appréciée selon les critères suivants (d'après critères Organisation Mondiale de la Santé - OMS) :

- **Certaine :**
  - l'événement survient dans un délai compatible avec l'utilisation du DM ;
  - il ne peut être expliqué par la maladie intercurrente ou par l'utilisation concomitante d'un autre traitement ;
  - l'évolution est favorable à l'arrêt du traitement ;
  - l'événement est expliqué par un mécanisme pharmacologique ou récidive lors de la réadministration ;

- **Probable :**
  - l'événement survient dans un délai raisonnable avec l'utilisation du DM ;
  - il ne peut être expliqué par la maladie intercurrente ou par la prise concomitante d'un autre traitement ;
  - l'évolution est favorable à l'arrêt du traitement ;
  - l'information sur la réadministration manque ou bien elle est peu claire.

- **Possible :**
  - l'événement survient dans un délai raisonnable avec l'utilisation du DM ;
  - il peut être expliqué par la maladie intercurrente ou par la prise concomitante d'un autre traitement ;
  - l'information sur la réadministration manque ou bien elle est peu claire.

- **Improbable :**
  - le délai d'apparition de l'événement après l'utilisation du DM rend la relation de cause à effet improbable ;
  - l'implication de la maladie sous-jacente ou la prise concomitante d'un autre traitement est à l'origine d'une autre explication plausible.

- **Exclue :**
  - l'évènement apparaît dans un délai incompatible par rapport à l'utilisation du DM ;
  - l'implication de la maladie sous-jacente ou la prise concomitante d'un autre traitement est à l'origine d'une autre explication vraisemblable.

Annexe 6 Formulaire de déclaration d'EIG

**FICHE DE DECLARATION D'UN EVENEMENT INDESIRABLE GRAVE**<br>(DISPOSITIF MEDICAL)<br>☞ A faxer dans les 48 heures au 09 81 40 42 80	Cadre réservé au CRPV<br>Date de réception: |___|___|/|___|___|/|___|___| Numéro : FR-CLB-________________________<br>Date de déclaration AC : |___|___|/|___|___|/|___|___|

PROTOCOLE HIFU

N° AFSSAPS (ANSM) : 2009-A00779-48	N° CLB : ET2009-068
☐ **Déclaration initiale**	☐ **Suivi n°** |__|__|

PATIENT (ou utilisateur: en cas d'incident chez une personne manipulant le dispositif, indiquez sa fonction)

N° d'inclusion : |___|___|/|___|___|
Initiales : |___|___|___|

1ère lettre du nom + 1ère lettre du prénom séparées par un tiret *(sauf prénom composé : 1ère lettre de chaque prénom sans tiret)*

Date de naissance : |___|___|/|___|___|/|___|___|
Date d'inclusion : |___|___|/|___|___|/|___|___|

Age : |___|___|
Poids (kg) : |___|___|___|

Sexe : ☐ Masculin ☐ Féminin

Phase de l'étude : ☐ I ☐ IIa1 ☐ IIa2 ☐ IIb

Antécédents ou facteurs de risque :____

____

DISPOSITIF MEDICAL (DM) A L'ETUDE

DM	Date d'utilisation	Localisation des tirs : foie sain, proximité des structures vasculaires, métasases	Nombre de lésions réalisées	Zone du foie traitée	Durée de la procédure HIFU
Dispositif MFOCUS					

Tolérance immédiate de la procédure HIFU, éventuelles difficultés rencontrées : ________________________________

____

Hépatectomie, zone réséquée : ____

Tolérance immédiate de la chirurgie : ____

(🖶 joindre systématiquement une copie du compte-rendu de l'intervention)

MEDICAMENTS ASSOCIES OU TRAITEMENTS HABITUELS

Médicament - Dosage	Voie	Posologie	Date de début	Date d'arrêt	Indication
			|__|__|/|__|__|/|__|__|	|__|__|/|__|__|/|__|__|	
			|__|__|/|__|__|/|__|__|	|__|__|/|__|__|/|__|__|	
			|__|__|/|__|__|/|__|__|	|__|__|/|__|__|/|__|__|	
			|__|__|/|__|__|/|__|__|	|__|__|/|__|__|/|__|__|	

EVENEMENT INDESIRABLE GRAVE

Date de début : |__|__|/|__|__|/|__|__| **Date de fin :** |__|__|/|__|__|/|__|__| **ou Durée** (j) **:** |__|__| **En cours** ☐

Description précise incluant les signes, les symptômes, l'organe concerné et la sévérité **(🖶 joindre systématiquement une copie du compte-rendu de consultation ou d'hospitalisation, des résultats biologiques et/ou d'imagerie si pertinent) :** ______

______

______

______

Diagnostic retenu par le praticien : ______

L'événement a-t-il nécessité l'interruption de la procédure HIFU ? ☐ oui ☐ non

Autre mesure prise : ______

Lieu de survenue (centre investigateur, hôpital, domicile, …) : ______

Evolution :

- ☐ Guérison sans séquelle
- ☐ Guérison avec séquelles, préciser : ______
- ☐ Décès Date : |__|__|/|__|__|/|__|__| Cause : ______
  - Autopsie : ☐ oui (joindre le CR) ☐ non ☐ prévue
- ☐ Evolution en cours, à documenter

Gravité :

- ☐ Décès
- ☐ Mise en jeu du pronostic vital
- ☐ Hospitalisation ou prolongation d'hospitalisation (joindre le compte-rendu)
  - Dates d'hospitalisation : du au
- ☐ Incapacité ou handicap important ou durable
- ☐ Anomalie ou malformation congénital
- ☐ Autre notion de gravité : ______

EVALUATION DU LIEN DE CAUSALITE

Avec le DM ➨ ☐ 0-Aucun ☐ 1-Improbable ☐ 2-Possible ☐ 3-Probable ☐ 4-Certaine

Avec le geste de mise en œuvre du DM ➨ ☐ 0-Aucun ☐ 1-Improbable ☐ 2-Possible ☐ 3-Probable ☐ 4-Certain

Précisez : ______

Avec une autre cause ➨ ☐ Chirurgie ☐ Traitement associé ☐ Pathologie ☐ Autre

Précisez : ______

COMMENTAIRES

______

______

Nom de l'investigateur : ______ **Tél :** |__|__| |__|__| |__|__| |__|__| |__|__|

Adresse : ______ **Fax :** |__|__| |__|__| |__|__| |__|__| |__|__|

Pays : ______ **Mail :** ______

Fait à : ______ Signature de l'investigateur :

le : |__|__|/|__|__|/|__|__|

Annexe 7 Avis favorable du CPP

Comité de Protection des Personnes SUD-EST IV

Centre Léon Bérard – 28 rue Laennec – 69373 LYON CEDEX 08

Président : Dr. Daniel ESPINOUSE - Vice-président : Mme Carine DIVER
Trésorier : Dr. David PEROL

Madame Anne LEFRANC
Centre Léon Bérard
28, rue Laennec
69008 LYON

Lyon, le 24 septembre 2009

N° CPP : 09/070
Réf. de la délibération : A 09-205
N° ID RCB : 2009-A00779-48

Le Comité a été saisi le 10 septembre 2009 par le Centre Régional Léon Bérard d'une demande d'avis sur un projet de recherche intitulé :
"Evaluation, chez des patients nécessitant une chirurgie de résection de métastases hépatiques de cancers colorectaux, de l'utilisation per-opératoire d'ultrasons focalisés de haute intensité (HIFU) : faisabilité, innocuité, et capacité de ciblage des métastases" **- Protocole n° ET2009-068, version 0.0 du 10 août 2009;**
Documents d'information et de consentement, version du 10 août 2009 ;
Brochure pour l'investigateur :

1. **réponses aux exigences essentielles prototype MFOCUS de juillet 2009 ;**
2. **spécifications système MFOCUS de novembre 2008 ;**

Liste des investigateurs, d'août 2009 ;
L'investigateur coordonnateur est le Pr. Michel RIVOIRE, Département de Chirurgie, Centre Léon Bérard à Lyon (69).

Le Comité a examiné les informations relatives à ce projet au cours de sa séance du 22 septembre 2009.

Membres présents à la séance :

- Personnes qualifiées "Recherche Biomédicale" : Mme M. MONTANGE (titulaire), Dr. D. ESPINOUSE (titulaire), Dr. Pascale CONY-MAKHOUL (suppléante), Mme Nicole FALETTE (suppléante), Mme Raymonde MARAVAL-GAGET (suppléante).
- Médecin généraliste : Dr. M. LE GAL (titulaire, qualifiée en matière de biostatistique).
- Infirmiers : Mme Saléa PRADAT (suppléante).
- Personnes qualifiées "Ethiques" : Mme Christiane KAPITZ (titulaire), Mme F. TOURAINE-MOULIN (suppléante).
- Personnes qualifiées "Juridique" : Mme C. DIVER (titulaire).
- Représentants d'associations de malades et d'usagers de la santé : Dr. Denis AZOULAY (titulaire).

.../...

Secrétariat : Odette MARITAZ
☎ 04 78 78 27 61 - 🖷 04 78 78 28 58 – E-mail : maritazo@lyon.fnclcc.fr

…/…

Le Comité a adopté la délibération suivante :

AVIS FAVORABLE

Le seul traitement curatif actuel des métastases hépatiques des cancers colorectaux est la résection hépatique, accessible uniquement à une minorité de patients. Toutes les alternatives à la chirurgie testées jusqu'à maintenant n'ont pas été satisfaisantes. Il est donc indispensable de développer de nouvelles techniques de destruction tumorale, non invasive et complémentaire à la chirurgie.

La présente étude se propose d'utiliser en per-opératoire des ultrasons focalisés de haute densité (HIFU). Les travaux pré-cliniques ont démontré l'intérêt, la faisabilité et la tolérance clinique et biologique d'un tel traitement.
Il s'agit de tester ce dispositif médical dans une étude de phase I-II en trois parties chez des patients devant subir une résection des métastases hépatiques par hépatectomie. Ce procédé n'a encore jamais été utilisé chez l'homme dans cette indication.

La balance bénéfices/risques est positive puisqu'il s'agit d'utiliser une technique couplée à l'imagerie sur une partie du foie qui sera réséquée. Le seul risque pour le patient est une anesthésie générale plus longue, sans excéder 30 minutes.

Dr. Daniel ESPINOUSE,
Président de séance

2/2

Secrétariat : Odette MARITAZ
☎ 04 78 78 27 61 - 🖷 04 78 78 28 58 – E-mail : maritazo@lyon.fnclcc.fr

Annexe 8 — Autorisation de l'AFSSAPS (ANSM)

RÉPUBLIQUE FRANÇAISE

afssaps
Agence française de sécurité sanitaire des produits de santé

Direction de l'évaluation des dispositifs médicaux
Unité veille et évaluations cliniques (UVEC)
Dossier suivi par Annie ROUSSEAU
Tél. +33 (0)1 55 87 36 96
Fax. : +33 (0)1 55 87 37 62
E-mail : dedim.dm@afssaps.sante.fr
N/Réf. : UVEC/AnnR/DA/2009- 230

Saint-Denis, le 23 SEP. 2009

Objet : Evaluation, chez des patients nécessitant une chirurgie de résection de métastases hépatiques de cancers colorectaux, de l'utilisation per-opératoire d'ultrasons focalisés de haute intensité : faisabilité, innocuité, et capacité de ciblage des métastases.
n° d'enregistrement : 2009-A00779-48

Madame,

Vous avez adressé à mes services une demande d'autorisation pour la recherche biomédicale intitulée : « Evaluation, chez des patients nécessitant une chirurgie de résection de métastases hépatiques de cancers colorectaux, de l'utilisation per-opératoire d'ultrasons focalisés de haute intensité : faisabilité, innocuité, et capacité de ciblage des métastases. ».et dont le numéro d'enregistrement est le 2009-A00779-48.

Au vu du dossier fourni, j'autorise cette recherche biomédicale en France. En vertu de l'article L 1121-4 du code de la santé publique, je vous précise toutefois que cette recherche biomédicale doit bénéficier d'un avis favorable du CPP pour pouvoir être mise en place en France.

Je vous rappelle notamment que, pendant le déroulement de la recherche et pour ce qui concerne l'Afssaps, toute modification substantielle du dossier initialement soumis doit faire l'objet d'une demande d'autorisation en vertu des articles L.1123-9 et R.1123-35 du code de la santé publique, les effets/évènements indésirables graves ainsi que les faits nouveaux susceptibles de porter atteinte à la sécurité des personnes sont à déclarer en vertu de l'article L.1123-10 du code de la santé publique, la fin de l'essai est également à déclarer en vertu de l'article L.1123-11 du code de la santé publique.

Je vous prie, d'agréer, Madame, l'expression de mes salutations distinguées.

Centre Léon BERARD
28 rue Laennec
69373 LYON CEDEX 08
France

A l'attention de Madame LEFRANC

Cc : CPP "Sud-Est IV"

Le chef de Département surveillance de marché

Nicolas THEVENET

143/147, bd Anatole France - F-93285 Saint-Denis cedex - tél. +33 (0)1 55 87 30 00 - www.afssaps.sante.fr

Annexe 9 — Attestation d'assurance

SHAM

ATTESTATION D'ASSURANCE

RESPONSABILITÉ CIVILE
PROMOTEUR DE RECHERCHES BIOMÉDICALES

(Loi n° 2004-806 du 9 août 2004 et textes d'application subséquents)

SOCIÉTÉ HOSPITALIÈRE D'ASSURANCES MUTUELLES
18, rue Edouard Rochet - 69372 LYON CEDEX 08

atteste que le CENTRE LEON BERARD
28 RUE LAENNEC
69373 LYON CEDEX 08

ont souscrit sous le n° **101.998** un contrat d'assurance de la Responsabilité Civile Promoteur de Recherche Biomédicale conforme aux dispositions du décret 2006-477 du 26 avril 2006, afin de couvrir les obligations mises à leur charge en application de l'article L.1121-10 du Code de la Santé Publique.

« HIFU_Evaluation, chez des patients nécessitant une chirurgie de résection de métastases hépatiques de cancers colorectaux, de l'utilisation per-opératoire d'ultrasons focalisés de haute intensité : faisabilité, innocuité, et capacité de ciblage des métastases. »
(PR RIVOIRE)

La garantie prend effet au plus tôt le **22 juillet 2009**, et est automatiquement acquise en cas notamment de modifications affectant le nombre de sujets ou la durée de la recherche.

La présente attestation ne constitue toutefois qu'une présomption d'assurance à la charge de la Société avant validation par les autorités compétentes.

Fait et Certifié, à LYON, le 22 juillet 2009

SOCIETE HOSPITALIERE
D'ASSURANCES MUTUELLES
22 JUIL. 2009
18, Rue Edouard Rochet - Lyon 8e

Gaëlle IGNACIO
Service Production

SHAM SOCIÉTÉ HOSPITALIERE D'ASSURANCES MUTUELLES
18 rue Édouard Rochet - 69372 LYON CEDEX 08
Tél : 33 (0)4 72 75 50 25 - Fax : 33 (0)4 72 74 22 32 - www.sham.fr

SOCIÉTÉ D'ASSURANCE MUTUELLE À COTISATIONS FIXES
ENTREPRISE RÉGIE PAR LE CODE DES ASSURANCES
N° TVA : FR 79779860881 - SIREN : 779 860 881 RCS LYON
RIP 20041 01007 0033305Z038 15

CERTIFIÉ ISO 9001
SHAM est certifié ISO 9001 pour ses prestations d'assurance, de formation et d'aide à la gestion des risques délivrées aux personnes morales.
